# Age‐related nitration/dysfunction of myogenic stem cell activator HGF

**DOI:** 10.1111/acel.14041

**Published:** 2023-11-20

**Authors:** Alaa Elgaabari, Nana Imatomi, Hirochika Kido, Takashi Nakashima, Shoko Okuda, Yoshitaka Manabe, Shoko Sawano, Wataru Mizunoya, Ryuki Kaneko, Sakiho Tanaka, Takahiro Maeno, Yuji Matsuyoshi, Miyumi Seki, So Kuwakado, Kahona Zushi, Nasibeh Daneshvar, Mako Nakamura, Takahiro Suzuki, Kenji Sunagawa, Judy E. Anderson, Ronald E. Allen, Ryuichi Tatsumi

**Affiliations:** ^1^ Department of Animal and Marine Bioresource Sciences, Graduate School of Agriculture Kyushu University Fukuoka Japan; ^2^ Department of Physiology, Faculty of Veterinary Medicine Kafrelsheikh University Kafrelsheikh Egypt; ^3^ Department of Bioscience and Biotechnology, Graduate School of Agriculture Kyushu University Fukuoka Japan; ^4^ Department of Orthopaedic Surgery, Faculty of Medical Sciences Kyushu University Fukuoka Japan; ^5^ Department of Biological Sciences, Faculty of Science University of Manitoba Winnipeg Manitoba Canada; ^6^ Department of Cardiovascular Medicine, Graduate School of Medicine Kyushu University Fukuoka Japan; ^7^ The School of Animal and Comparative Biomedical Sciences University of Arizona Tucson Arizona USA; ^8^ Present address: Department of Food and Life Science, School of Life and Environmental Science Azabu University Sagamihara Japan; ^9^ Present address: Department of Animal Science and Biotechnology, School of Veterinary Medicine Azabu University Sagamihara Japan

**Keywords:** age‐related muscle atrophy, fast myofiber, fibrosis, hepatocyte growth factor (HGF), peroxynitrite, regeneration, resident myogenic stem cell, tyrosine nitration

## Abstract

Mechanical perturbation triggers activation of resident myogenic stem cells to enter the cell cycle through a cascade of events including hepatocyte growth factor (HGF) release from its extracellular tethering and the subsequent presentation to signaling‐receptor c‐met. Here, we show that with aging, extracellular HGF undergoes tyrosine‐residue (Y) nitration and loses c‐met binding, thereby disturbing muscle homeostasis. Biochemical studies demonstrated that nitration/dysfunction is specific to HGF among other major growth factors and is characterized by its locations at Y198 and Y250 in c‐met‐binding domains. Direct‐immunofluorescence microscopy of lower hind limb muscles from three age groups of rat, provided direct in vivo evidence for age‐related increases in nitration of ECM‐bound HGF, preferentially stained for anti‐nitrated Y198 and Y250‐HGF mAbs (raised in‐house) in fast IIa and IIx myofibers. Overall, findings highlight inhibitory impacts of HGF nitration on myogenic stem cell dynamics, pioneering a cogent discussion for better understanding age‐related muscle atrophy and impaired regeneration with fibrosis (including sarcopenia and frailty).

## Graphical abstract

Myogenic stem cell activator HGF undergoes nitration of Y198, 250 and loses signaling‐receptor binding. Nitration is specific to HGF among other major growth factors. Immunofluorescence microscopy of rat lower hind limb muscles, provided direct evidence for age‐related increases in nitration of ECM‐bound HGF, preferentially stained for anti‐nitrated Y198, 250 in fast IIa and IIx fibers, pioneering a cogent discussion for better understanding age‐related atrophy and impaired regeneration.

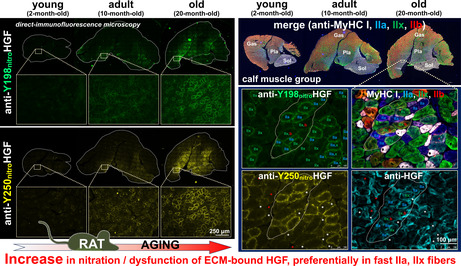

**Abbreviations**
ASA: accessible surface area
BrdU: 5‐bromo‐2'‐deoxyuridine
BSA: bovine serum albumin
ECL: enhanced chemiluminescence
ECM: extracellular matrix
EDL: extensor digitorum longus
FGF: fibroblast growth factor
HF: highly frequent
HGF: hepatocyte growth factor
HRP: horseradish peroxidase
IGF: insulin‐like growth factor
mAb: monoclonal antibody
MyHC: myosin heavy chain
SC: side chain
SDS: sodium dodecylsulfate
TA: tibialis anterior
TGF: transforming growth factor
Y: tyrosine residue
βME: 2‐mercaptoethanol

## 1 INTRODUCTION

Skeletal muscle is a major tissue that early exhibits age‐related reduction in mass and function, manifested as distinct disorders ranging from mild muscle weakness to atrophy and impaired muscle regeneration with fibrosis, intramuscular fat infiltration, and formation of small (thinner and shorter than normal) myofibers after injury. The progression and accumulation of the age‐related phenotypes result from the disruption/dysfunction of molecular mechanisms involved in coordinating normal muscle growth and regeneration (Conboy & Rando, 2005; Dorrens & Rennie, 2003; Janssen, 2004, 2010; Montano, 2014; Sousa‐Victor et al., 2021; Taylor et al., 2023).

Muscle homeostasis and regeneration largely rely on a population of resident myogenic stem satellite cells, which are positioned between the basal lamina and the sarcolemma of postnatal myofibers and normally found in a mitotically and metabolically quiescent or near‐dormant state (protracted G_1_ phase, also referred to G_0_) in adult muscle (Anderson, 2021; Bischoff & Franzini‐Armstrong, 2004). When muscle is injured, overused, or mechanically stretched, satellite cells are activated to enter the cell cycle, proliferate to produce myoblast progeny, differentiate, and fuse with existing muscle fibers or form new fibers. Therefore, satellite cell activation is an initial and crucial step in postnatal myogenesis. Activation also enables self‐renewal of myogenic stem cells, although recent studies mention an “early activation” phase characterized by transition toward more activated stem cell forms (“more responsive state” mediated by a mechano‐sensitive ion channel Piezo1) (Ma et al., 2022) and rapid retraction of cytoplastic projections necessary for stem cell activation (Kann et al., 2022).

Our previous studies demonstrated that satellite cell activation is triggered by mechanical perturbation through a cascade of events including release of the active form of hepatocyte growth factor (HGF; heterodimer of α‐ and β‐chains) from its extracellular tethering in a nitric oxide (NO) radical‐dependent manner, and the subsequent presentation of HGF to the cell‐membrane signaling‐receptor c‐met (Allen et al., 1995; Anderson, 2000; Hara et al., 2012; Tatsumi et al., 1998, 2001, 2002; Tatsumi & Allen, 2004, 2008; Tatsumi, Liu, et al., 2006; Tatsumi, Wuollet, et al., 2009; Tatsumi, Yamada, et al., 2006; Yamada et al., 2006, 2008). Therefore, regenerative functions of quiescent cells may be tightly regulated and affected by the extracellular microenvironment that enables HGF‐c‐met interaction (Pallafacchina et al., 2010; Wang et al., 2020; Yin et al., 2013). The significance of the regulatory role played by the extracellular microenvironment has been clearly demonstrated in experiments with aging skeletal muscle that showed that the youthful environment in young muscle facilitates adequate muscle growth and regeneration (Carlson & Faulkner, 1989). Thus, age‐related alteration/disruption of the extracellular microenvironment may be manifested as muscle atrophy with impaired regeneration, although a niche‐driven molecular mechanism(s) has not been clearly resolved (Boonen & Post, 2008; Jackson et al., 2020; Perandini et al., 2018; Schüler et al., 2021; Wosczyna & Rando, 2018).

Increased generation of NO and superoxide (O_2_^−^) radicals may be a major contributor that initiates and/or stimulates the progression and accumulation of age‐related phenotypes. NO radical is a key intermediate element in physiological and pathophysiological events in muscle and a variety of other tissues (Anderson, 2000; Anderson & Pilipowicz, 2002; Di Meo et al., 2016; Ischiropoulos, 1998; Powers et al., 2016; Singh et al., 2013; Tatsumi, 2010; Tatsumi et al., 2002; Tidball et al., 1998; Wozniak & Anderson, 2009). NO cytotoxicity is attributed mainly to the formation of peroxynitrite (ONOO^−^) (Bartesaghi & Radi, 2018; Bohle et al., 2004; Pacher et al., 2007; Radi, 2004, 2013; Szabó et al., 2007; Tidball et al., 1998). Peroxynitrite is known to induce protein nitration, a post‐translational chemical‐modification process for the non‐enzymatic introduction of a nitro group (‐NO_2_) into the side chain of tyrosine residues (and rarely, tryptophans); tyrosine nitration results in alterations in protein activity and function. Protein tyrosine nitration is a highly selective event and can be evaluated for its potential impact by tracking its accumulation as a peroxynitrite “footprint” in tissues (Ischiropoulos, 2009; Viner et al., 1996). Accumulation of specific‐protein nitration has been observed in a variety of disorders including aging (Fugere et al., 2006; Pandya et al., 2019) and Alzheimer's and Parkinson's diseases (Good et al., 1998; Horiguchi et al., 2003; Reyes et al., 2008), although a direct causal relationship remains to be proven.

These findings provide a justification for exploring the possibility that the myogenic stem cell activator HGF undergoes nitration and dysfunction (loss of functions) progressively during aging. Direct biochemical evidence was obtained in the present study by exposing recombinant whole HGF to short‐lived or steady fluxes of peroxynitrite under physiological conditions and by evaluating nitroY198/250‐containing synthetic peptides for the c‐met binding activity. Results demonstrated that the nitration takes place at Y198 and Y250 characterized by their locations at the c‐met binding sites of the K1 and K2 domains of HGF α‐chain, respectively, and the nitration‐caused dysfunction is specific to HGF among the other major growth factors examined (FGF2, IGF1, and TGF‐β3). The physiological significance of these emerging findings was further encouraged by visualizing the nitration of extracellular matrix (ECM)‐bound HGF (Y198 and Y250) in rat lower hind limb muscles. Nitrated HGF was observed predominantly on fast IIa and IIx myofibers and increased with aging, highlighting that accumulation of nitrated HGF may be a key microenvironmental event to impact age‐related muscle phenotypes including intramuscular fibrosis and impaired regeneration. The study presents conceptual advances of unusual significance regarding a major discussion topic in age‐related disorders of muscle homeostasis including muscle atrophy and impaired regeneration along with fibrosis (sarcopenia and frailty).

## 2 METHODOLOGY

All information can be found online in the Supporting Information section at the end of this article.

## 3 RESULTS

### 3.1 An inhibitory element of satellite cell activation

**FIGURE 1:**
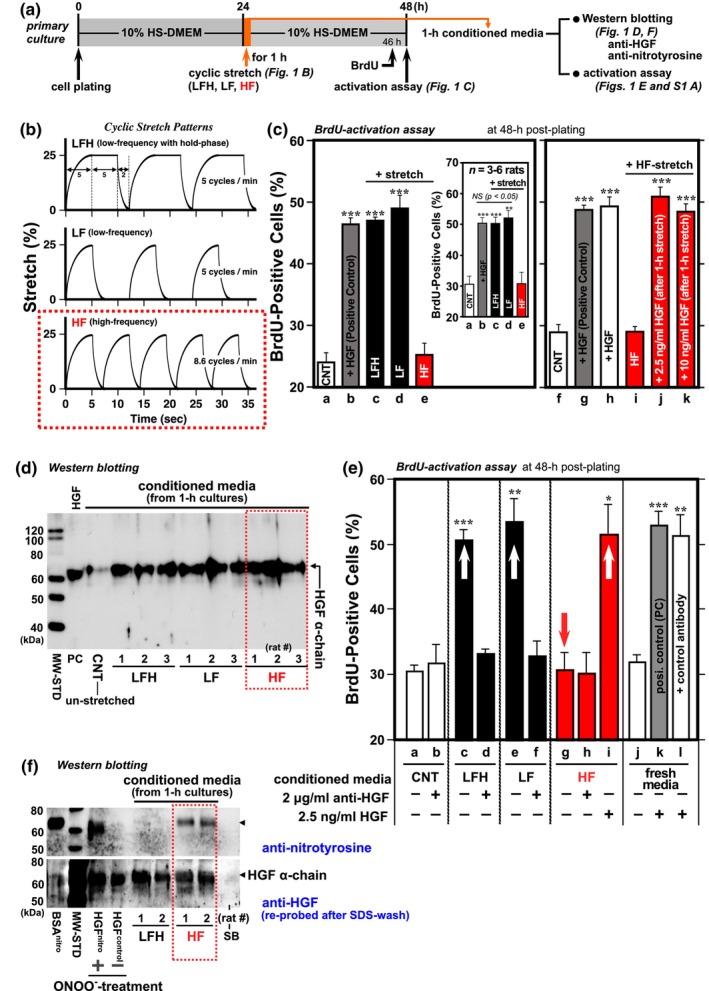
High‐frequency (HF) stretch does not activate satellite cells. (a) The experimental scheme for stretch cultures. Primary cultures of rat satellite cells received one of three patterns of 25% cyclic stretch for 1 h beginning at 24‐h post‐plating followed by pulse labeling with BrdU for 2 h just prior to the 48‐h, time‐point of the BrdU‐incorporation assay for activation. Conditioned media from 1‐h stretch cultures were also evaluated by western blotting and BrdU‐incorporation. (b) Three cyclic‐stretch patterns, LF, LFH, and HF, differed in intensity, as determined by stretch frequency (5.0 or 8.6 cycles/min) with/without 5‐s stretch‐retention, at 5‐s intervals. (c) Cell activation response to three cyclic‐stretch patterns. Satellite cell cultures were assayed for cell activation (BrdU‐positive cell percentage) at 48‐h post‐plating. CNT, negative control unstretched culture in DMEM‐10% HS (*open bars a* and *f*); positive controls, cultures with 2.5 ng/mL recombinant HGF for 24 h beginning 24‐h post‐plating (*gray bars b* and *g*) and for 23 h beginning 25‐h (*open bar h*) before fixation. Stretch cultures: 1‐h LFH, LF, and HF stretch (*black bars c and d*, and *red bar e*, respectively, in the left column). The experiments were repeated using 3–6 rats independently to confirm the reproducibility of the results (panel c inset). *Red bar i*, HF‐stretch culture again (under the same experimental condition as *bar e*); *red bars j* and *k*, 1‐h HF stretch followed by administration of 2.5 ng/mL and 10 ng/mL HGF for 23 h after stretch, respectively. (d) Immunoblotting analysis of HGF in conditioned media from 1‐h control and stretch cultures (visualized with anti‐HGF polyclonal antibody). MW‐STD, molecular weight standards (MagicMark Western Protein Standard); PC, recombinant HGF (with BSA of a carrier protein). CNT, control conditioned media from unstretched cells; LFH, LF, and HF, media from stretch cultures (*n* = 3 rats each stretch‐pattern group), normalized at constant number of cells after stretch. (e) Activation activity of conditioned media from 1‐h stretch cultures. Conditioned media were collected from control unstretched (CNT) and stretch cultures (LFH, LF, and HF), incubated with 2 μg/mL anti‐HGF neutralizing antibody (AB‐294‐NA from R&D Systems) (*bars b, d, f*, and *h*) or control IgG (*bar l*) for 30 min at 4°C followed by feeding to unstretched cultures for 24 h beginning 24‐h post‐plating. Experiments included a control set of cultures that were maintained in freshly prepared media (DMEM‐10% HS) with/without 2.5 ng/mL HGF (*bars j–l*). Note that there was no activation activity shown by conditioned medium from HF stretch (*red bar g*) as the BrdU‐incorporation level was comparable to control cultures (*bars a* and *j*), and was rescued by addition of recombinant HGF to the medium (*red bar i*). Bars represent mean ± SEM, and significant differences from the negative control (CNT) at *p <* 0.01 are indicated by double asterisks (**) (panels c and e). (f) Visualization of nitrated HGF in HF‐stretch conditioned media by western blotting. Blots were treated with HRP‐labeled anti‐nitrotyrosine mAb (upper row), followed by stripping with SDS‐βME solution and re‐probing with HRP‐labeled anti‐HGF α‐chain mAb (lower row; immuno‐positive α‐chain was indicated by arrowhead). Lanes 1–9 (from left to right): nitrated BSA (positive control), MW‐STD (MagicMark molecular weight standards), recombinant HGF incubated with and without peroxynitrite (ONOO^−^), conditioned media from 1‐h LFH‐ and HF‐stretch cultures (2 rats each stretch group), and SDS‐βME sample buffer (SB).

The present question about aging muscle originated when reviewing unpublished results from a former research project that revealed mechanical stretch‐triggered HGF/NO‐dependent activation of quiescent myogenic stem satellite cells in vitro and in vivo. Over two decades ago, we encountered an un‐explainable data set beginning with the finding that highly‐frequent (HF) stretch does not activate satellite cells, while regular low‐frequent stretch cultures (LFH and LF) normally activate cells to a level equivalent to a positive control culture (2.5 ng/mL HGF) as shown in Figure 1 (panels b and c, *bars a–e*). The results are highly reproducible as demonstrated by repeating experiments independently using 3–6 rats (Figure 1c inset). As shown in Figure 1c right column, HF stretch does not abolish cell viability including physiological activity of cell‐membrane receptor c‐met as revealed by post‐stretch experiments showing that HF‐stretched cells were normally activated to a level equivalent to the control cultures (with 2.5 ng/mL HGF for 24 h from 24 to 48 h post‐plating and for 23 h from 25 to 48 h post‐plating mimicking the HF‐stretch culture without stretch; *bars g and h*, respectively) in response to exogenous HGF after 1‐h HF stretch (*bars j and k*, 2.5 and 10 ng/mL HGF in media, respectively). Importantly, conditioned media from 1‐h cyclic‐stretch cultures (LFH, LF, and HF) contained a comparable amount of HGF when standardized with cell counts as shown by ECL‐western blotting; this indicates that there is no detectable difference in the release of HGF from the extracellular matrix (ECM), a key event that allows binding to c‐met, between three stretch patterns (Figure 1d; *n* = 3 media from independent cultures per stretch‐pattern). Contrary to this result, HF‐stretch conditioned medium did not show satellite cell activation activity (Figure 1e, *bar g*; in contrast to *bars c and e* of LFH and LF‐stretch media, respectively), but that activity was rescued by adding recombinant HGF (2.5 ng/mL) to the HF‐stretch medium (*bar i*) and in part, by mixing with equal volume of LFH‐stretch medium (Figure S1, *bar d*).

To provide a rational explanation and physiological significance for these apparently contradictory results, we re‐opened the experiments and proposed a possible hypothesis that a myogenic stem cell activator, HGF, receives chemical modification to abrogate its biological activity in response to HF stretch. Conditioned media from 1‐h HF‐ and LFH‐stretch cultures were evaluated for nitration of tyrosine residues by western blotting; a 60‐kDa band (the same chain weight as HGF α‐chain) was detected by HRP‐labeled anti‐nitrotyrosine mAb exclusively in HF‐stretch media (Figure 1f). This preliminary result supports the chemical modification hypothesis, HGF nitration‐driven inhibition of satellite cell activation.

### 3.2 Nitration and dysfunction of HGF by peroxynitrite

**FIGURE 2:**
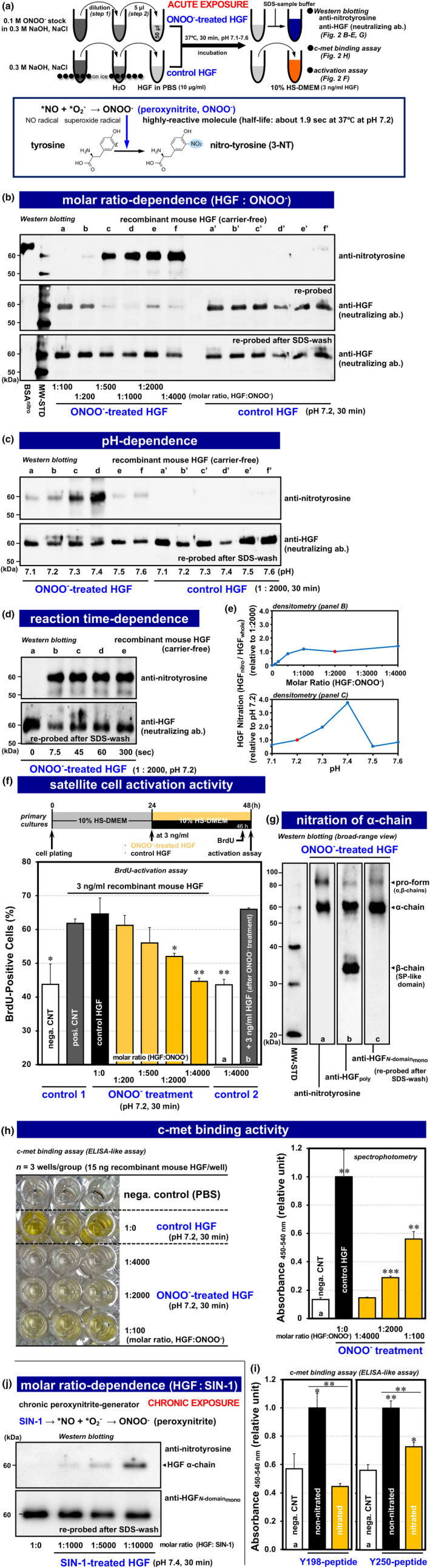
Peroxynitrite induces nitration/dysfunction of HGF. (a) In vitro experimental design for acute exposure of recombinant HGF to peroxynitrite (panels a–h). Two‐step dilution protocol for the peroxynitrite stock (0.1 M ONOO^−^ on ice) was optimized for the exposure of recombinant HGF (carrier protein‐free) to active peroxynitrite in order to examine whether HGF undergoes nitration and dysfunction under physiological conditions. Lower column is a supplemental sketch of the tyrosine‐residue nitration, peroxynitrite‐induced nitrotyrosine formation by introduction of a nitro group (‐NO_2_) into a Cε atom in aromatic ring of the side chain, of which surface exposure indexed by the ASA value (see Table S1) contributes to high nitration‐selectivity of tyrosine residues. (b) Visualization of HGF nitration status by ECL‐western blotting (peroxynitrite molar ratio dependence). Recombinant mouse HGF (disulfide‐linked heterodimer of 60‐kDa α‐chain and 30‐kDa β‐chain as the major form in the product) was exposed to peroxynitrite in a wide range of molar ratios relative to HGF (1:0–1:4000) at pH 7.2, 37°C for 30 min. Blot was first treated with HRP‐conjugated anti‐nitrotyrosine mAb (upper row), followed by direct re‐probing with anti‐HGF polyclonal antibody (mid row; neutralizing antibody from R&D Systems) and then by re‐probing with the same brand of anti‐HGF polyclonal antibody after stripping with SDS‐βME solution (lower row). Short 50–70 kDa range that covers 60‐kDa HGF α‐chain (including the NK2 segment responsible for c‐met binding) was displayed; panel g shows that the remaining 30‐kDa β‐chain was negative for nitration of tyrosine residues. BSA^nitro^, nitrated BSA (a positive control for immunodetection of nitrotyrosine); MW‐STD, MagicMark molecular weight standards; *lanes a–f*, peroxynitrite‐treated HGF (1:100–1:4000), and *lanes a′–f′*, control HGF (1:0) incubated in treatment buffer at pH 7.2 without peroxynitrite for 30 min. (c) pH dependence of HGF nitration by peroxynitrite treatment (pH 7.1–7.6, at 1:2000, 37°C for 30 min). Blot was treated with HRP‐labeled anti‐nitrotyrosine antibody, chemically stripped with SDS‐βME, and re‐probed by anti‐HGF antibody as in panel b, lower row. *Lanes a–f*, peroxynitrite‐treated HGF (pH 7.1–7.6), *lanes a′–f′*, the corresponding control HGF. (d) Reaction time dependence of HGF nitration upon peroxynitrite treatment (in a range of 0–300 s; at 1:2000, pH 7.2, 37°C). Blot was treated with HRP‐labeled anti‐nitrotyrosine and anti‐HGF antibodies in the same way as panel c (*lanes a–e*). (e) Densitometry for blots shown in panels b and c. Nitration levels of HGF were normalized by whole HGF (detected by re‐probing with anti‐HGF polyclonal antibody after the stripping step) and expressed as relative to the values of 1:2000 molar ratio (upper row) and pH 7.2 groups (lower row) indicated by red circles. (f) Dysfunction of peroxynitrite‐treated HGF. Satellite cell cultures were maintained in DMEM‐10% HS with peroxynitrite‐treated HGF (3 ng/mL at the final concentration; treated at 1:200–1:4000 of molar ratios, pH 7.2, for 30 min) for 24‐h period beginning 24‐h post‐plating, and pulse‐labeled with BrdU for 2 h to measure BrdU‐positive cell percentages as described in Figure 1c,e (*orange bars*; see upper column for the experimental scheme). Control HGF, non‐nitrated HGF (1:0; *black bar*) in treatment buffer without peroxynitrite treatment. The experiment included two control groups: control set #1 included regular negative‐control culture in DMEM‐10% HS (nega. CNT; *far left open bar*) and positive‐control culture with 3.0 ng/mL HGF (posi. CNT; *gray bar*); control set #2 is an important set of second control cultures incubated only with a medium for the 1:4000 peroxynitrite treatment (without HGF; *open bar a*) and with 3 ng/mL HGF added back to the post‐peroxynitrite treatment (same condition as the 1:4000 treatment; *gray bar b*). (g) Immunolocalization of nitration sites in HGF α‐chain. Broad‐range view (20–100 kDa) of western blotting of HGF treated with peroxynitrite (at 1:4000, pH 7.4, 37°C, for 30 min). MW‐STD, MagicMark molecular weight standards; *lane a*, a blot treated with HRP‐labeled anti‐nitrotyrosine mAb; *lane b*, re‐probed with anti‐HGF polyclonal antibody after stripping with SDS‐β‐ME; *lane c*, re‐probed with HRP‐labeled anti‐HGF α‐chain (*N*‐domain) mAb after the second round of chemical stripping. (h) Receptor c‐met binding activities of peroxynitrite‐treated HGF. Evaluated by sandwich ELISA‐like assay on solid‐phase of recombinant c‐met‐Fc chimera (see left photo of the triplicate assay). Optical absorbance_450–540 nm_ was presented as a relative unit to the control HGF (right column; *black bar*, 1:0 peroxynitrite treatment served as a positive control). *Open bar a*, negative control without HGF (nega. CNT); *orange bars*, HGF treated with peroxynitrite at 1:4000, 1:2000, and 1:100 at pH 7.2 for 30 min (from left to right). (i) c‐Met binding activities of Y198‐peptide (left column; 7.5 μM FTSNPEVR_nitro/non‐nitro_Y_198_EV) and Y250‐peptide of HGF (right column; 0.15 μM KFLPER_nitro/non‐nitro_Y_250_PDKGFD), evaluated by the sandwich ELISA‐like assay (triplicate assay). Optical absorbance was presented as a relative unit to each non‐nitrated peptide (*black bars*, served as positive controls). *Open bars a*, negative controls without peptides (nega. CNT); *orange bars*, nitroY198‐ and Y250‐peptides. Bars represent mean ± SEM and significant differences from the control HGF (*black bar*) in panel f and the negative control (*bar a*) in panels h and i, at *p <* 0.05, *p <* 0.01, and *p <* 0.001 are indicated by (*), (**), and (***), respectively. (j) Nitration status of HGF α‐chain in response to SIN‐1. Visualized by western blotting of HGF treated with SIN‐1, a chronic generator of peroxynitrite, at 1:0, 1:1000, 1:5000, and 1:10,000 of molar ratios to HGF, pH 7.4, 37°C for 30 min. Blots were treated with HRP‐labeled anti‐nitrotyrosine antibody followed by stripping with SDS‐βME and re‐probing with HRP‐labeled anti‐HGF α‐chain mAb.

To test the hypothesis mentioned above, experiments were designed to first examine whether HGF undergoes nitration and dysfunction in response to peroxynitrite (ONOO^−^) (Figure 2). Recombinant HGF was incubated with peroxynitrite at various molar ratios relative to HGF (1:100–1:4000 of HGF:peroxynitrite) for 30 min at pH 7.2, 37°C and evaluated for its nitration level by ECL‐western blotting with HRP‐labeled anti‐nitrated tyrosine (nitrotyrosine, 3‐NT) mAb followed by densitometry analyses normalized by total HGF (Figure 2b,e, upper columns). Results illustrate that nitrated HGF α‐chain (60 kDa) was clearly recognized by probing with the anti‐nitrotyrosine antibody in a peroxynitrite dose‐dependent manner (*lanes a–f*), in contrast to undetectable immuno‐reactivity of untreated control HGF (*lanes a′–f′*). Loading HGF amounts on the blot were comparable between the peroxynitrite‐treated and untreated control lanes, as revealed by stripping with SDS‐βME solution and re‐probing the membrane with regular anti‐HGF polyclonal antibody (Figure 2b, lower column). Interestingly, when the SDS‐βME wash step was omitted, the immuno‐reactivity of the anti‐HGF polyclonal neutralizing antibody (R&D Systems; heavily used in our previous studies) (Elgaabari et al., 2022; Tatsumi et al., 1998, 2001, 2002; Tatsumi, Liu, et al., 2006; Tatsumi, Sankoda, et al., 2009) drastically decreased with nitration of HGF (Figure 2b, mid column). The suppression may be explained by steric hindrance resulting from the close proximity (or partial overlap) of the binding sites of neutralizing anti‐HGF and anti‐nitrotyrosine antibodies, indicating that the amino acid sequence forming the c‐met binding site of HGF contains a tyrosine residue that undergoes nitration. Indeed, a tyrosine residue 198 has been identified to be essential for the c‐met binding site in a K1 domain of HGF α‐chain (Lokker et al., 1994; Watanabe et al., 2001).

The subsequent biochemical study revealed that HGF nitration is characterized by a defined pH dependence with a peak at pH 7.4 (Figure 2c,e, lower columns) and by a rapid reaction that completes within seconds (at least 7.5 s, the earliest able time‐point) in consistent with a short half‐life of peroxynitrite (about 1.9 s at pH 7.2, 37°C) (Figure 2d). To further define and characterize the susceptibility of HGF for nitration, an additional experiment was conducted using SIN‐1, which is a doner of both NO and O_2_^−^ radicals with a slow decomposition rate (half‐life of 14–26 min) that enables chronic generation and exposure of a steady flux of peroxynitrite in an in vivo‐mimicking manner (Martin‐Romero et al., 2004). As shown in Figure 2j, SIN‐1 treatment (at pH 7.4 for 30 min) promoted nitration of HGF tyrosine residues in a dose‐dependent manner. These results indicate that HGF undergoes nitration of tyrosine residues upon exposure to peroxynitrite under physiological conditions in vitro.

The physiological impact of this post‐translational event was examined by evaluating myogenic stem cell activation activity of nitrated HGF (Figure 2f). Primary cultures of rat satellite cells were incubated with peroxynitrite‐treated HGF (at pH 7.2 for 30 min) for 24 h beginning at 24‐h post‐plating followed by measurement of 5‐bromo‐2′‐deoxyuridine (BrdU)‐positive cell percentages. The activation index was decreased further at higher peroxynitrite molar ratios, down to a level equivalent to a negative‐control culture at the ratio of 1:4000 (*orange bars*). The experiment included an important second set of control cultures: a negative control (*bar a*) just with a medium for the peroxynitrite treatment at 1:4000 and a HGF‐treated culture (*bar b*) showing that the activation index was rescued to a level comparable to the control HGF‐treated culture (1:0; *solid black bar*) by 3 ng/mL HGF added back to a post‐peroxynitrite treatment (1:4000), indicating that a metabolite of peroxynitrite does not abolish satellite cell activation but peroxynitrite‐treated HGF loses its activation activity.

The functional consequence of HGF nitration was supported by localization of nitrotyrosine residues in the active form of HGF (heterodimer of α‐ and β‐chains) (Figure 2g). Western blotting of peroxynitrite‐treated HGF demonstrated that HGF α‐chain, the subunit responsible for c‐met binding, is essentially the recipient of nitration activity targeting tyrosine residues by showing that both the 60‐kDa α‐chain and the 90‐kDa pro‐form (not yet cleaved into α‐ and β‐chains) were immuno‐positive for anti‐nitrotyrosine and anti‐*N*‐terminal PAN domain (also called *N*‐domain) mAbs (*blots a and c*); neither antibody showed significant immuno‐reactivity to the 30‐kDa β‐chain of HGF (*blots a and c*), which was visualized after chemical stripping of the blot (SDS‐βME wash) and re‐probing with anti‐HGF polyclonal antibody (*blot b*). The result is supported by our pilot study reporting that a recombinant *N*‐terminal NK1 segment (PAN domain and the subsequent K1 domain that contains a c‐met binding site of HGF α‐chain) undergoes nitration of a tyrosine residue(s) and dysfunction of c‐met binding upon peroxynitrite treatment (Elgaabari et al., 2022). This issue was further examined in experiments in which nitrated HGF was evaluated directly for its c‐met‐binding activity by a sandwich ELISA‐like assay (Figure 2h). Binding affinity was reduced to a baseline level (*bar a*, a negative control) depending on nitration status, shown as dose dependent to the molar ratio of peroxynitrite to HGF (in 1:100, 1:2000, and 1:4000 molar ratios relative to HGF; *orange bars*). These results highlight a possible mechanism for dysfunction of nitrated HGF: In its central configuration, the nitrated HGF α‐chain cannot bind to the signaling‐receptor c‐met and this abolishes the function of HGF to promote myogenic stem cell activation and subsequent proliferation.

**FIGURE 3:**
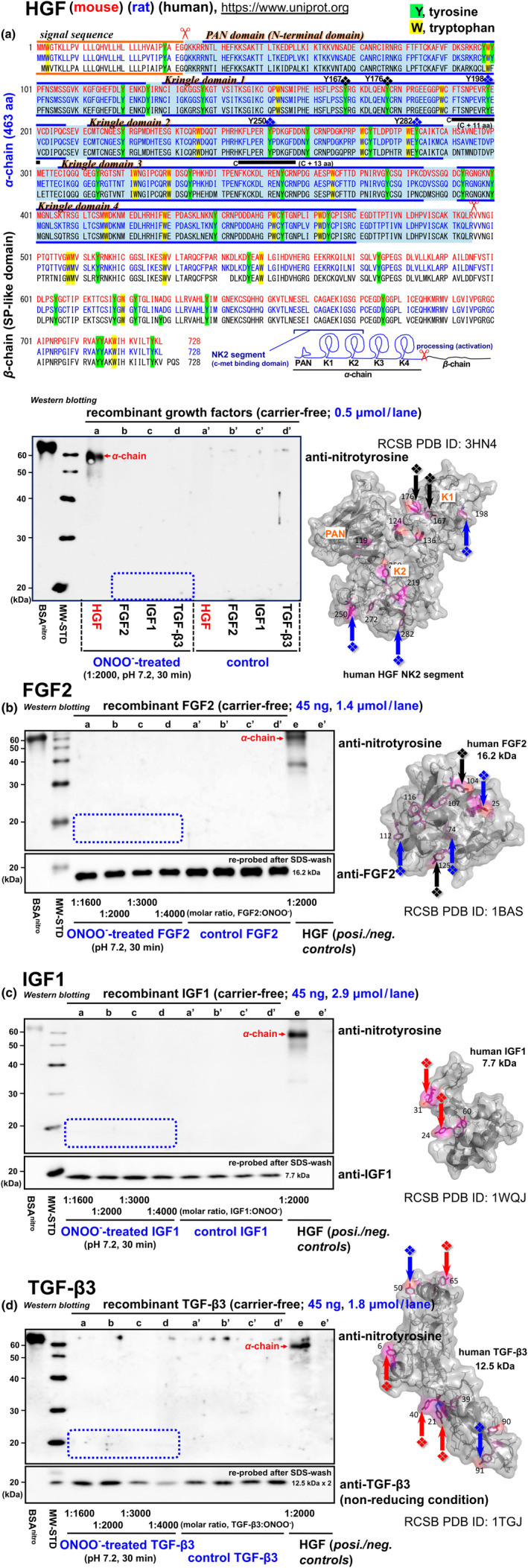
HGF nitration is a unique event among major growth factors. (a) Comparative display of entire primary structures of the multi‐domain sequence for mouse (red), rat (blue), and human HGF (black). Original data are from UniProt (https://www.uniprot.org). Tyrosine (Y) and tryptophan (W) residues in α‐chain (composed of *N*‐terminal and the subsequent K1–K4 domains with baby‐blue background) and β‐chain (also called SP‐like domain) are highlighted by green and yellow, respectively. Tyrosine residues having SC‐ASA values 30–50 Å^2^, 50–100 Å^2^, and over 100 Å^2^ are indicated by black, blue, and red rhomboids, respectively, in the NK2 segment of HGF α‐chain (*N*‐terminal (PAN) + K1 + K2 domains; see Table S1 for more details); two conserved sequences that include Y198 and Y250, FTSNPEVRY_198_EV and KFLPERY_250_PDKGFD underlined in black were custom synthesized in the forms containing nitroY198 and Y250 (also see Figure S2A,B). The high‐resolution crystal structure of the human NK2 segment (Recacha et al., 2012) was drawn to focus on the side chain of all tyrosine residues (pink‐highlighted) by the program PyMOL Molecular Graphics System as same as for other human growth factors shown in panels b–d right columns. Lower row, recombinant growth factors, FGF2, IGF1, and TGF‐β3, were evaluated for the nitration potential by peroxynitrite treatment (at 1:2000, pH 7.2, for 30 min) and western blotting at the same molar ratios as for HGF, using HRP‐labeled anti‐nitrotyrosine antibody (0.5 μmol per well, *lanes b–d*). BSA^nitro^, nitrated BSA (*lane 1*) served as a positive control along with nitrated HGF (*lane c*); MW‐STD, MagicMark molecular weight standards (*lane 2*); non‐nitrated controls (1:0; *lanes a′–d′*). Boxed area, a region where immuno‐response is expected. (b–d) FGF2 (Zhu et al., 1991) (RCSB PDB ID: 1BAS), IGF (Siwanowicz et al., 2005) (PDB ID: 1WQJ), and TGF‐β3 (Mittl et al., 1996) (PDB ID: 1TGJ) were evaluated for their nitration activities in response to peroxynitrite (at 1:1600–1:4000, pH 7.2, for 30 min), by western blotting at the same protein‐mount basis as HGF (45 ng/lane: 1.4 μmol FGF2, 2.9 μmol IGF1, 1.8 μmol TGF‐β3, 0.5 μmol HGF per lane; *lanes a–e*). The blots were treated with HRP‐labeled anti‐nitrotyrosine antibody followed by stripping with SDS‐βME and re‐probing with individual primary antibodies. *Lanes a′–e′*, controls (1:0) incubated in treatment buffer at pH 7.2 without peroxynitrite for 30 min.

Notably, peroxynitrite‐induced nitration/dysfunction is a unique event for HGF among major growth factors that are all essential for successful muscle growth and regeneration, as revealed by additional western blotting analyses of recombinant human proteins (Figure 3). In brief, there was no immuno‐response to HRP‐labeled anti‐nitrotyrosine antibody on the blots of recombinant FGF2, IGF1, and TGF‐β3 that were treated with peroxynitrite in a molar‐ratio range (1:1600, 1:2000, 1:3000, and 1:4000) under the same physiological conditions (at pH 7.2, 37°C for 30 min) and evaluated at the same molar basis as HGF (0.5 μmol protein/lane; Figure 3a) and the same weight basis (45 ng protein/lane: 0.5 μmol HGF, 1.4 μmol FGF2, 2.9 μmol IGF1, 1.8 μmol TGF‐β3 per lane; Figure 3b–d). These results further define the unique physiological significance of nitration/dysfunction of the myogenic stem cell activator HGF, and a strict regulation that ensures high nitration‐selectivity for tyrosine residues along the whole HGF molecule (34 residues that are highly conserved in mammals; see green‐highlighted Ys in Figure 3a upper column, and Figure S2A).

An initial regulatory element required for the nitration sites of HGF α‐chain may be the surface exposure of the tyrosine side chain, which was indexed by the accessible surface area (ASA) values for side‐chain (SC) atoms and the Cε atoms (% Cε_1,2_/SC) of tyrosine residues in the HGF NK2‐segment (NK1 segment plus the subsequent K2 domain that includes a c‐met binding site separate from the K1 domain) along with the references of FGF2, IGF1, and TGF‐β3 (see Table S1; calculated by AREAIMOL based on the primary and crystal structures obtained from UniProt and RCSB PDB, respectively). As depicted in Figure 3a, lower right column, five tyrosine residues (Ys) have SC‐ASA values greater than 30 Å^2^ (indicated by arrows); the two largest, Y198 and Y250, were selected as nitration‐target candidates for K1 and K2 domains, respectively. Reference proteins FGF2, IGF1, and TGF‐β3 had five, two, and six tyrosine residues with higher SC‐ASA values than HGF, respectively (see yellow‐highlighted in Table S1 and indicated by arrows in Figure 3b–d, right columns), but none of these tyrosine residues have nitration activity detectable by western blotting as described previously (Figure 3b–d, left‐columns), indicating that extremely strict regulatory mechanisms other than surface exposure of the tyrosine side chain are at work.

According to the SC‐ASA analysis for HGF‐NK2 segment, two antigen sequences that include nitrated Y198 and Y250 were designed to produce rat mAbs specific to nitrated Y198‐HGF and nitrated Y250‐HGF (designated 3A11C6 and 6B82C3, respectively, with same immunoglobulin isotype of rat IgG2a(κ), raised in‐house), which are sole tools that enable visualizing nitration of HGF K1 and K2 domains separately in tissue samples under non‐reducing and reducing conditions (Figure S5). Importantly, these mAbs recognized peroxynitrite‐treated HGF, as shown by ELISA and western blotting (Figure S5C–E), hence providing the first evidence that Y198 and Y250 receive nitration in response to peroxynitrite under physiological conditions and thus that when the c‐met binding sites (containing Y198 and Y250) of HGF undergo nitration, the c‐met binding function is lost. In particular, synthetic peptides containing nitrated Y198 and Y250, FTSNPEVR_nitro_Y_198_EV, and KFLPER_nitro_Y_250_PDKGFD (the same sequences as the immunogens; not treated with peroxynitrite but synthesized in a nitrated form with nitroY198 and Y250) did not show significant c‐met binding activity (Figure 2i, *orange bars*) as revealed by low levels comparable to negative controls (*bars a*) in the sandwich ELISA‐like assay, while the non‐nitrated peptides significantly did (*black bars*). In this c‐met binding assay, the non‐nitrated Y198‐peptide required a higher molar concentration than the Y250‐peptide (7.5 μM vs. 0.15 μM), indicating that the affinity for c‐met is much lower than for nitrated Y250, possibly due to an insufficient number of amino acids on the C‐terminal side of the nitrated tyrosine residue. Nevertheless, the result provides direct evidence to show that nitration of Y198 and Y250 diminishes the c‐met binding of the K1 and K2 domains of the HGF α‐chain.

### 3.3 In vivo demonstration: extracellular‐HGF nitration with aging

**FIGURE 4:**
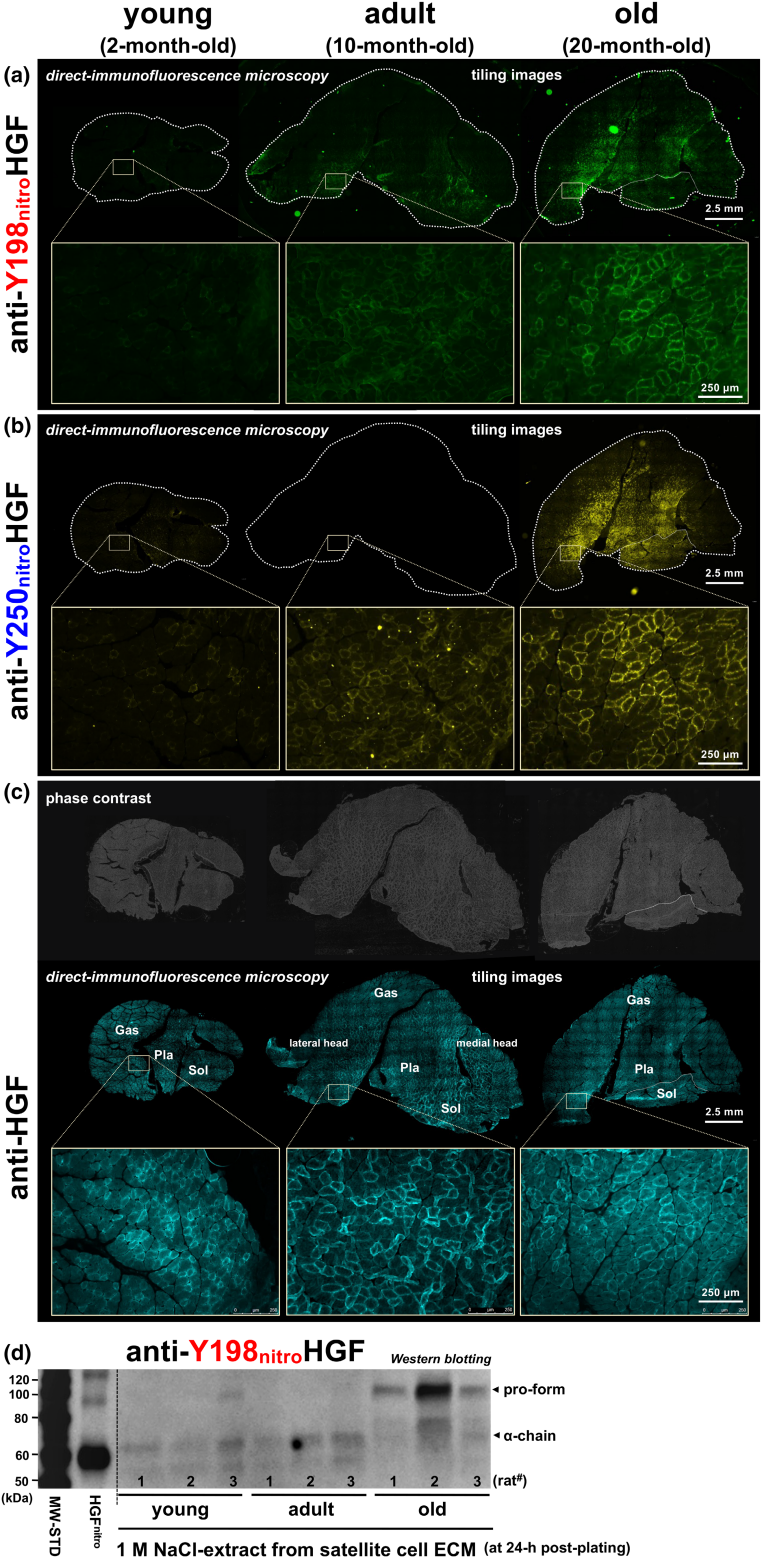
Extracellular HGF undergoes Y198/Y250 nitration during aging. (a–c) Direct‐immunofluorescence microscopy evidence of in vivo nitration. Lower hind limb muscles were collected from male S.D. rats of three age groups, young (2‐month‐old), adult (10‐month‐old), and old (20‐month‐old) (*n* = 3 each group). Serial cryo‐sections of calf muscle (mid‐belly portion, 13‐μm thickness) were blocked with sterile donkey serum/BSA/gelatin solution prior to incubation with Fluorescein‐labeled anti‐nitrated Y198‐HGF (clone 3A11C6; fluorescent false‐green) (a), HiLyte Fluor 647‐labeled anti‐nitrated Y250‐HGF (clone 6B82C3; fluorescent false‐yellow) (b), or Alexa Fluor 594‐labeled anti‐HGF α‐chain antibody (clone H‐10 from Santa Cruz Biotechnology; fluorescent false‐baby‐blue) (c). Sections were mounted in VECTASHIELD Antifade Mounting Medium and observed under a fluorescence microscope equipped with digital cameras and the Tile‐Scan program. Representative images of three rats per age group are shown here (see Figure S6 for other specimens). Gas, gastrocnemius; Pla, plantaris; Sol, soleus muscle. Lower row of each panel, high‐magnification views of boxed areas in an anterior‐side region of Gas lateral head (upper row, tiling images). (d) ECM‐bound HGF nitration monitored by western blotting. Extraction of ECM‐associated HGF was conducted by 1.0 M NaCl wash of isolated satellite cells at 24‐h post‐plating (originally developed by Naldini et al., 1992) from the three age groups of rats (*n* = 3 rats per group) and subjected to western blotting using anti‐nitrated Y198‐HGF mAb to monitor the nitration levels of extracellular HGF normalized to cell counts on the plates. The α‐chain and pro‐form of HGF are indicated by arrowheads. MW‐STD, MagicMark molecular weight standards; HGF^nitro^, nitrated recombinant HGF.

Conceptual extension of emerging in vitro findings was performed by testing the potential role of peroxynitrite‐induced HGF nitration (at Y198 and Y250 in K1 and K2 domains, respectively) and dysfunction in the age‐associated decline in skeletal muscle homeostasis. Age‐related nitration was examined by in vivo aging experiments, in which lower hind limb muscles were collected from three age groups of S.D. rats, young (2‐month‐old), adult (10‐month‐old), and old (20‐month‐old), and evaluated for nitration levels of ECM‐bound HGF by direct‐immunofluorescence microscopy in serial cross‐sections of specimens (*n* = 3 rats per group) (Figure 4a–c). Figure 4 panel (a) displays a representative set of calf muscles (gastrocnemius, plantaris, and soleus muscles) stained in a single run with Fluorescein‐labeled anti‐nitrated Y198‐HGF mAb (clone 3A11C6 raised in‐house; fluorescent false‐green). Nitrated HGF was detected at the periphery of myofibers in adult muscle (10‐month‐old) and a remarkable increase (accumulation) observed in old age (20‐month‐old) in plantaris, soleus, and gastrocnemius (anterior‐side region of medial and lateral heads and the mid‐body portion). Similar age‐linked HGF nitration pattern was seen by immunostaining of serial cryo‐sections of calf muscles with HiLyte Fluor 647‐labeled anti‐nitrated Y250‐HGF antibody (clone 6B82C3; Figure 4b, fluorescent false‐yellow) and shank muscles, extensor digitorum longus (EDL) and tibialis anterior (TA) muscles, with the anti‐nitrated Y198 and Y250‐HGF antibodies (Figure 6a). Importantly, staining against HGF_total_ (labeling both nitrated and non‐nitrated HGFs) looked comparable in its amount among the three age groups, as visualized by companion staining of calf muscles with Alexa Fluor 594‐labeled anti‐HGF α‐chain antibody (clone H‐10) (Figure 4c, fluorescent false‐baby‐blue); results were reproducible in all three rats per age group, as validated in Figure S6. Overall, considering that HGF has been found to be present in an ECM‐bound state (Tatsumi et al., 1998, 2001, 2002; Tatsumi & Allen, 2004; Tatsumi, Liu, et al., 2006), these in vivo observations indicate that extracellular HGF undergoes nitration of tyrosine residues (Y198 and Y250) with aging.

This emerging finding was supported by western blotting analyses of an ECM‐bound protein fraction, namely that is extracted by a 1 M NaCl wash from primary satellite cell cultures (prepared from thigh and back muscles followed by maintaining for 24 h after plating) and may possess extracellular materials associated with their niche along myofibers as in vivo “memory” at the early phase after plating and before proliferation. As shown in Figure 4d (*n* = 3 rats per age group), a faint band of nitrated HGF α‐chain (60 kDa) was detected in NaCl extracts from cultures from adults (10‐month‐old) by anti‐nitrated Y198‐HGF mAb at slightly a higher level than in cultures from young (2‐month‐old) rats, normalized to cell count. Interestingly, in cultures from old rats (20‐month‐old), 90‐kDa HGF (the inactive form, yet to be cleaved into α‐ and β‐chains) was exclusively detected by anti‐nitrated Y198‐HGF antibody while the 60‐kDa α‐chain was not significantly seen, indicating that extracellular HGF may be nitrated in its inactive form in muscle from old‐age rats.

Physiological signatures of aging in the rats used here are displayed in supplemental materials (Figure S8); muscle weight for the group of calf (gastrocnemius, plantaris, and soleus muscles) showed a significant decrease between adult and old age (panel a). Fiber‐type composition analyses by quadruple‐immunofluorescence microscopy for cross‐sections of calf muscles showed a significant increase in proportion of type I (slow, fluorescent false‐white/gray) and a decrease in type IIa (fast, fluorescent false‐blue) fibers in old muscle with a marked decrease in total myofiber numbers in plantaris and soleus muscles (panels b and c). As shown in panel (d), modified Masson‐trichrome staining visualized the progression of age‐related fibrosis (indexed by increase in intramuscular collagen stained in blue) in wide areas of shank (EDL and TA muscles) and calf muscles (in particular, in soleus and the anterior aspect of the mid‐body portion of gastrocnemius between medial and lateral heads). These results are consistent with the major features of age‐related phenotypes of skeletal muscles in human (decrease in muscle weight as an index of muscle atrophy, decrease in the fast‐fiber ratio, and up‐regulation of fibrogenesis), thus assuring that the experimental design including the rat ages (in vivo assay time‐points) was appropriate to the conclusion that extracellular HGF undergoes tyrosine‐residue nitration (Y198 and Y250) to lose c‐met binding with aging.

### 3.4 Extracellular HGF nitration preferentially in fast IIa and IIx myofibers

Notably, further examination of immunofluorescence micrographs of the old‐age muscles (20‐month‐old) (Figures 4a,b and S6A) showed again that the nitration level of extracellular HGF is heterogeneous, even within individual muscles examined, especially in gastrocnemius muscle with the accumulation of nitrated HGF preferentially at the anterior aspect where slow‐twitch (type I) and fast‐twitch fibers (types IIa and IIx) are abundant in contrast to other regions (posterior peripheral belly) that are extremely rich in fast IIb fibers (Tatsumi et al., 2017). This important observation is not an artifact due to technical problems including the freeze‐cut angle relative to the muscle periphery, as revealed by the phase‐contrast image showing normal round‐shaped cross‐sectional images of myofibers (Figure 4c, upper row). At higher magnification views (see lower rows of Figure 4a,b), the heterogeneity is further defined by noting that some fibers are remarkably immuno‐positive for both anti‐nitrated Y198‐HGF and Y250‐HGF antibodies while others show only weak or negative (non‐significant) staining, suggesting a possible relationship between susceptibility to nitration and myofiber types.

**FIGURE 5:**
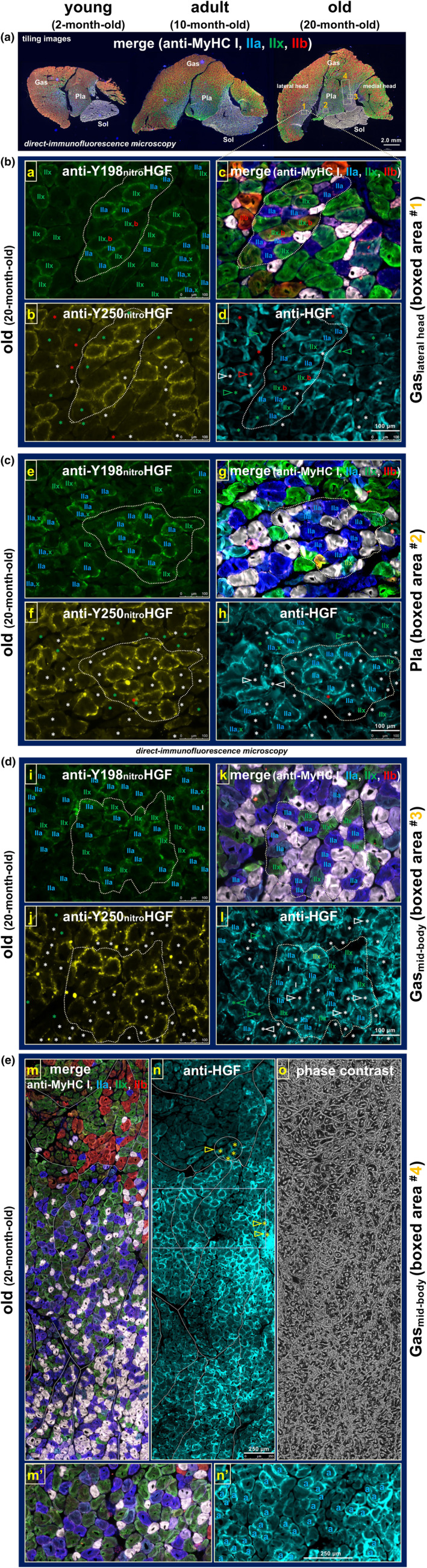
Relationship between HGF nitration and fiber types (calf muscle). (a) Visualization of myofiber types of calf muscles from three age groups, young (2‐month‐old), adult (10‐month‐old), and old (20‐month‐old). Cryo‐sections of calf muscle (mid‐belly portion) were quadruple‐immunostained with direct‐labeling fluorescent probes‐conjugated to anti‐MyHC isoform mAbs (slow (type I): fluorescent false‐white/gray, fast IIa: false‐blue, fast IIx: false‐green, and fast IIb: false‐red). Representative merged micrographs from imaging of muscle from three rats (one from each age group are presented; tiling images). Gas, gastrocnemius; Pla, plantaris; Sol, soleus muscle are indicated. (b–d) Direct‐immunofluorescence staining of calf muscle in the old group (20‐month‐old) gives in vivo evidence for preferential nitration of extracellular HGF in fast IIa and IIx myofibers. Nitrated HGF‐rich areas in gastrocnemius muscle lateral head (b; boxed area #1 depicted in panel a) and mid‐body (d; boxed area #3) and in plantaris muscle (c, boxed area ^#^2) were evaluated at high magnification for the relationship between fiber types and nitration levels of ECM‐bound HGF by direct‐immunofluorescence staining of serial cryo‐sections with the same set of quadruple anti‐MyHC isoform mAbs as in panel a (*images c, g, and k*) and anti‐nitrated Y198‐HGF (*images a, e, and i*) and anti‐nitrated Y250‐HGF mAbs (*images b, f, and j*). Note the notable proportions of nitrated HGF‐positive fast IIa and IIx fibers (indicated by “IIa” and “IIb” in upper left *images a, e, and i*) along with negative/faint fast IIb and IIx, and slow (i) fibers (indicated by red, green, and white asterisks in the lower rows of panels b–d, respectively). Another serial section was stained with Alexa Fluor 594‐labeled anti‐HGF α‐chain mAb to visualize total HGF (nitrated and non‐nitrated HGF, false‐baby‐blue; *images d, h, and l*); extracellular HGF exists predominantly in fast IIa, IIx fibers with some exceptions in HGF‐positive type I and IIb fibers (indicated by white and red arrowheads, respectively). Green arrowheads, HGF‐positive fast IIx fibers with negative/faint nitration levels. Dotted lines were drawn on the micrographs to adjust the alignment of the four images in each panel. (e) Direct‐immunofluorescence of fiber types and total HGF in boxed area #4 in a mid‐body region of gastrocnemius muscle (see panel a, right micrograph from old rat). Note that, consistent with boxed areas # 1–3, HGF‐rich fibers are fast IIa (indicated by “a” in *image n’*, higher magnification view of a boxed area in *image n*) and fast IIx, while slow (type I) and fast IIb fibers are negative/faint with some exceptions for fast IIb fibers (indicated by yellow asterisks and arrowheads in *image n*).

**FIGURE 6:**
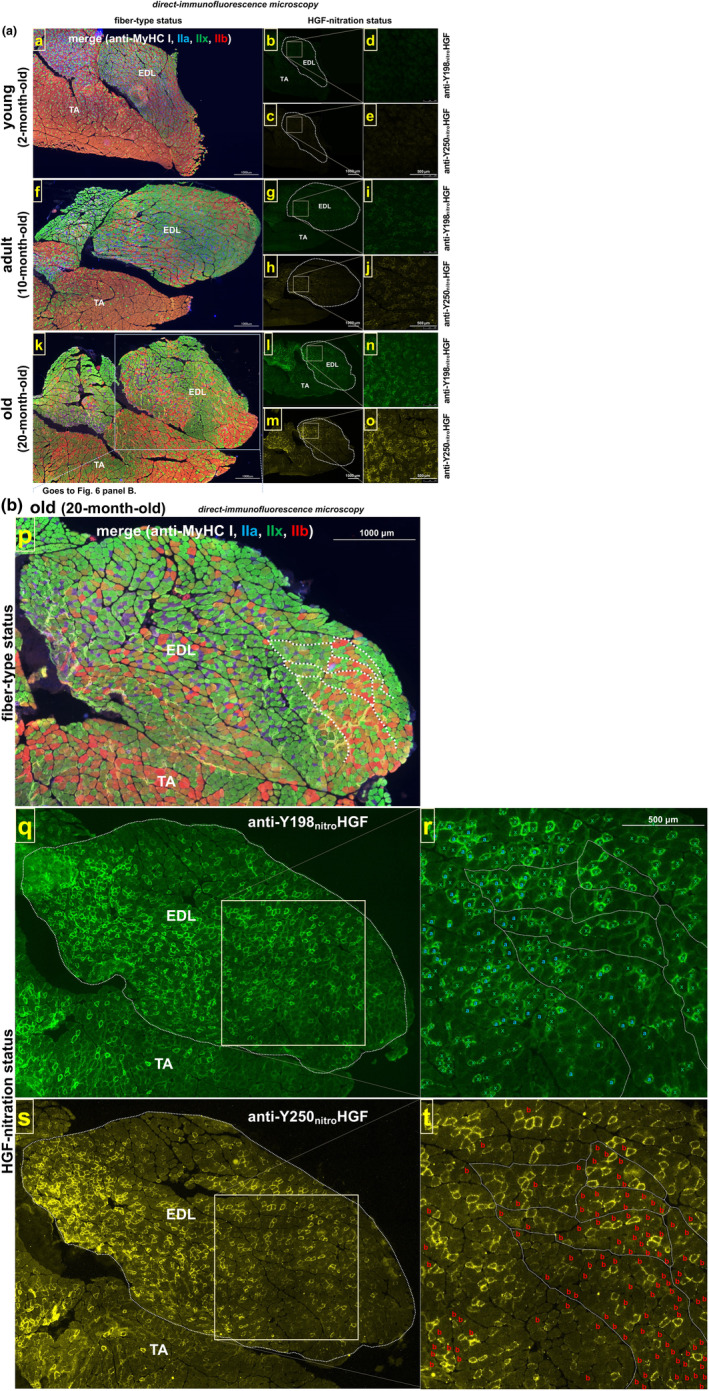
Relationship between HGF nitration and fiber types (shank muscle). (a) Direct‐immunofluorescence staining of shank muscle group (TA and EDL muscles) from three age groups (*n* = 3 rats per group), young (2‐month‐old), adult (10‐month‐old), and old (20‐month‐old): supportive in vivo evidence for nitration of extracellular HGF (Y198 and Y250) during aging. Serial cryo‐sections of TA and EDL muscle group (mid‐belly portion) were stained with a set of quadruple anti‐MyHC isoform mAb (merged micrographs; slow (type I): fluorescent false‐white/gray, fast IIa: false‐blue, fast IIx: false‐green, and fast IIb: false‐red), and Fluorescein‐labeled anti‐nitrated Y198‐HGF (fluorescent false‐green) and HiLyte Fluor 647‐labeled anti‐nitrated Y250‐HGF mAbs (fluorescent false‐yellow), and observed under the fluorescence microscope, as described in Figure 4 (same settings to standardize photographic‐exposure time). Higher magnification views of framed areas (corresponding to a nitrated HGF‐rich region in old rats; see *images l and m* in the mid column) are shown in the right column. (b) Supportive evidence for preferential nitration of ECM‐bound HGF in fast IIa and IIx myofibers. Another set of serial cryo‐sections of TA and EDL muscle group in old rat muscle (20‐month‐old) was stained with anti‐MyHC isoform mAbs (*image p*) and anti‐nitrated Y198‐HGF (mid row) and Y250‐HGF (lower row) mAbs, as described for panel a, and analyzed for the relationship between HGF nitration level and fiber type. Higher magnification views of a boxed area are shown in the right column; note that nitration‐positive fibers are fast IIa and IIx (indicated by “a” and “x” in *image r*, respectively). Few slow (type I) fibers were observed, and fast IIb fibers were negative/faint for extracellular HGF nitration (indicated by “b” in *image t*). Dotted lines were drawn on the micrographs (*images p, r, and t*) to adjust the alignment of the images.

To examine this issue directly, a single specimen of the calf muscle group of an old rat (20‐month‐old) was cut in serial cryo‐section for either quadruple‐immunostaining with mAbs for the different MyHC isoforms that had each been pre‐labeled with a different fluorescent probe (Figure 5a, far‐right micrograph with low magnification; slow (type I): fluorescent false‐white/gray, fast IIa: false‐blue, fast IIx: false‐green, and fast IIb: false‐red), or immunostained for nitrated HGF at Y198 or Y250 residues. Areas rich in nitrated HGF were selected from the lateral head and mid‐body of the gastrocnemius muscle (Figure 5b,d; boxed areas ^#^1 and ^#^3, respectively) and the plantaris muscle (boxed area ^#^2, panel c) and evaluated at high magnification for the linkage between fiber types (*images c, g, and k*) and the level of ECM‐bound HGF nitration visualized separately by immunostaining of serial cryo‐sections with anti‐nitrated Y198‐HGF (*images a, e, and i*) and anti‐nitrated Y250‐HGF mAbs (*images b, f, and j*). Boxed area ^#^1 displaying all fiber types showed noticeable proportions of fibers positive for nitrated HGF in fast IIa and IIx fibers (and rare fast IIx/IIb chimera) along with negative/faint nitration of extracellular HGF in fast IIb and slow (I) fibers (panel b; indicated by red and white asterisks in *image b*, respectively). The same manner of fiber‐type‐dependent HGF nitration was seen in area #2 (panel c, plantaris muscle) and area #3 (panel d, a mid‐body region of gastrocnemius muscle), and also in EDL muscle in which the proportion of fast IIb fibers was higher than in areas #1–3 without significant HGF nitration (Figure 6b). It may be important to note that not all fast IIx fibers were always positive for HGF nitration even in muscle from old rats (20‐month‐old; indicated by green asterisks in *images b, f, and j*); these negative IIx fibers might either become nitrated during further aging or be highly resistant to HGF nitration through an unknown mechanism, and thereby still contributing to the maintenance of requisite (declining) muscle functions. By comparison, fast IIa fibers all appeared positive for HGF nitration at the same age (20‐month‐old), indicating that fast IIa fibers may be more susceptible to HGF nitration/dysfunction than IIx fibers during aging. Overall, the in vivo aging experiments (Figures 4, 5, 6, S6, and S8) demonstrated that ECM‐bound HGF undergoes Y198/Y250 nitration and dysfunction preferentially on fast IIa and IIx myofibers with aging while slow (type I) and fast IIb fibers are negative/faint for the nitration of extracellular HGF in rat lower hind limb muscles in old age (20‐month‐old).

Considering the paucity of fast IIb myofibers in humans, ECM‐bound HGF nitration of fast IIa and IIx fibers may be a key element in a mechanism of fast fiber‐preferential atrophy and impaired regeneration during aging in humans. This fiber‐type‐dependent HGF nitration may result from in part the predominant presence of extracellular HGF in fast IIa and IIx fibers as revealed by immunostaining of total HGF (nitrated and non‐nitrated moieties) to show that extracellular HGF exists predominantly in fast IIa and IIx fibers with some exceptions of HGF‐positive type I and IIb fibers (indicated by white and red arrowheads, respectively; Figure 5b–e, *images d, h, l, n, and n′*). Similar results were seen in Figure 5e (boxed area #4 in gastrocnemius muscle, a mid‐body region having all fiber types), in which a significant level of HGF was immuno‐detected in fast IIa and IIx fibers and less prominent in type I and IIb fibers (with a few exceptions for fast type IIb, indicated by yellow asterisks and arrowheads in *image n*).

## 4 DISCUSSION

Resident myogenic stem satellite cells play pivotal roles in skeletal muscle maintenance and regeneration (Anderson, 2006, 2021; Bischoff, 1990; Chargé & Rudnicki, 2004; Collins et al., 2005; McCormick & Schultz, 1994; Schultz & McCormick, 1994; Wozniak et al., 2005). The activation of quiescent satellite cells to enter the cell cycle is an initial and crucial step for these homeostatic events (Anderson, 2006; Bischoff & Franzini‐Armstrong, 2004; McCormick & Schultz, 1994), and HGF is a dominant myogenic stem cell activator (Allen et al., 1995; Tatsumi et al., 1998; Tatsumi & Allen, 2004; reviewed in Anderson, 2006, 2021, Tatsumi & Allen, 2008, and Tatsumi, 2010). It is well established that mechanical stretch impacts satellite cell activation through a cascade of biochemical events centered on NO radical‐dependent activation of matrix metalloproteinase‐2 (MMP2) and/or MMP9, liberation of extracellular HGF (ECM‐bound HGF, ECM stocked with the active form in muscles of adult animals), and the subsequent binding of 60‐kDa α‐chain of HGF to the cell‐membrane signaling‐receptor c‐met (Anderson, 2000; Tatsumi et al., 1998, 2001, 2002; Tatsumi & Allen, 2004; Tatsumi, Liu, et al., 2006; Tatsumi, Wuollet, et al., 2009; Tatsumi, Yamada, et al., 2006; Yamada et al., 2006, 2008). A significant point of discussion remains focused on the kinetics of satellite cell activation in response to various patterns and intensity of stretch, including the LFH, LF, and HF stretch protocols examined in this study. The HF stretch, which is characterized by a cyclic‐stretch frequency just 1.72‐times higher than LF and LFH‐stretch modalities that both normally activate satellite cells in culture, fails to trigger satellite cell activation despite the lack of a significant effect on cell viability, including the physiological activity of the receptor c‐met. Similarly, HF‐stretch medium did not show satellite cell activation activity (in contrast to LFH/LF‐stretch media) and was rescued by adding recombinant HGF to the HF medium. Nevertheless, release of HGF from extracellular tethering occurs normally after HF at a level comparable to that in cultures after LF and LFH stretch (Figure 1). These unpublished results from HF‐stretch experiments opened a prologue toward verification of a possible inhibitory mechanism that is centered on a post‐translational modification of released HGF and/or ECM‐bound HGF and differs from high‐HGF concentration‐induced expression of myostatin that re‐establishes satellite cell quiescence (Yamada et al., 2010). Indeed, anti‐nitrotyrosine antibody clearly detected a 60‐kDa band by western blotting of conditioned media from HF‐stretch cultures and not from LFH‐stretch cultures (Figure 1f), indicating that HGF α‐chain (60 kDa), which is directly responsible for c‐met binding, may undergo nitration of tyrosine residues by the HF stretch.

This opening result was followed by biochemical experiments, which provided direct evidence for the nitration/dysfunction of HGF by showing that recombinant HGF undergoes nitration of Y198 and Y250 residues in K1 and K2 domains, respectively, in a c‐met binding site in the α‐chain, in response to acute and chronic exposure to peroxynitrite under physiological conditions with an optimal pH 7.4 and that this chemical modification disrupts the c‐met binding activity and results in the loss of activation functionality of HGF (Figures 2a–i and S5). The finding was achieved by use of anti‐nitrated Y198, Y250‐HGF mAbs raised in‐house (see Figures S4 and S5), which detected nitration of Y198 and Y250 by ELISA and western blotting of peroxynitrite‐treated recombinant whole HGF (Figure S5, panels c–e) and the NK1 segment (Elgaabari et al., 2022). It is evident that the cause of HGF dysfunction is not the c‐met receptor, because satellite cells normally responded to HGF added to a post‐peroxynitrite treatment (Figure 2f *bar b*). Importantly, based on the cryo‐EM structure of a complex of HGF and the receptor c‐met dimer (RCSB PDB ID: 7MO7) (Uchikawa et al., 2021), both Y198 and Y250 localize in the c‐met binding sites (see Figure S3C, redrawn to focus on tyrosine residues), supporting the inhibitory impact of Y198/Y250 nitration on c‐met binding of the NK2 region, possibly due to conformational alteration of the binding sites by nitration. In fact, as shown in Figure 2i, two synthetic nitroY198 and nitroY250‐peptides (FTSNPEVR_nitro_Y_198_EV and KFLPER_nitro_Y_250_PDKGFD, not treated with peroxynitrite) lack c‐met binding activity, supporting the conclusion that nitration of Y198 and Y250 abolishes the c‐met binding function of the HGF molecule (both the inactive and active forms). The biochemical potential for nitration of tryptophan residues (shown just in SOD1 as far as we know, also called Cu, Zn‐superoxide dismutase) (Yamakura et al., 2001, 2005), and oxidation of the thiol group of the cysteine residue side chain and a methionine residue (Met155) by peroxynitrite (Daiber et al., 2013; Peixoto et al., 2018; Perrin & Koppenol, 2000) does not appear to participate in this inhibitory mechanism, as discussed previously (Elgaabari et al., 2022).

Tyrosine nitration does not occur at random (not all proteins undergo nitration); rather, it shows a differential sensitivity/susceptibility toward tyrosine nitration under the same physiological conditions that may result from the structural attributes of a target protein (Ischiropoulos, 2003; Souza et al., 1999). Exposure of a tyrosine aromatic ring from the protein surface facilitates the initial attack of peroxynitrite compared to a buried tyrosine (Batthyány et al., 2005) and can be indexed by ASA values of SC atoms and the Cε atoms of tyrosine residues (Table S1). As well, residues in the vicinity of tyrosine are extremely critical for susceptibility of tyrosine nitration; a high nitration tendency is observed for tyrosine residues proximate to glutamic acid (E) (Ng et al., 2013), in contrast to neighboring cysteine (C) that acts as an intramolecular scavenger for peroxynitrite, therefore trapping it away from tyrosine residue (Abello, 2009; Petruk et al., 2012). Also, a glycine residue (G) is rarely found in the vicinity of nitrated tyrosine (Ng et al., 2013). When re‐examining the amino acid sequences (Figures 3a and S2A,B) and SC‐ASAs calculated for tyrosine residues in a 3D‐model of the HGF‐NK2 segment (Table S1), strict nitration‐regional selectivity ensures that Y198 and Y250 are the most preferential nitration targets in the NK2 segment and that other all tyrosine residues having high‐ASA indices, Y167, Y176, and Y282, may be outside of nitration sites. The same thing was true for the other growth factors examined, FGF2, IGF1, and TGF‐β3, which were negative for western blotting detection of tyrosine nitration upon acute exposure to peroxynitrite (Figure 3). Overall, the present in vitro study provides a robust background platform for understanding the unique physiological significance of nitration/dysfunction of the myogenic stem cell activator HGF.

This exciting question was addressed in the present study; results may contribute to understanding the possibility that the generation and accumulation of specific nitrated proteins in certain locations in particular tissues and organs have the potential to be a biomarker for the onset and progression of several disorders and diseases including cardiovascular damage (Mihm, 2001; Shishehbor, 2003), chronic obstructive pulmonary disease (COPD) (Barreiro et al., 2003), colon and lung cancer (Gochman et al., 2011; Markowitz et al., 2017; Zhan et al., 2018), two neurodegenerative disorders, Alzheimer's and Parkinson's diseases (Castegna et al., 2003; Good et al., 1998; He et al., 2018; Horiguchi et al., 2003; Reyes et al., 2008), and aging (Chakravarti & Chakravarti, 2017; Fugere et al., 2006; Viner et al., 1996), although the direct causal relationship remains unclear, awaiting further studies. During bio‐physiological aging, even under basal conditions, a variety of tissues and organs ineffectively maintains metabolic “quality management” homeostasis through a metabolic shift and redox deviation toward highly oxidative and nitrosative circumstances accompanied by reduced stress‐tolerance (Aoi & Sakuma, 2011; Gomes et al., 2017; Mecocci et al., 1999; Sousa‐Victor & Muñoz‐Cánoves, 2016). This age‐associated metabolic remodeling in many tissues, together with the increasing biochemical evidence for nitration‐induced HGF dysfunction with peroxynitrite‐concentration and pH dependencies, encourages consideration that possibly unifying mechanism, centered on the generation and accumulation of nitrated HGF, may be responsible for the progressive decline in homeostasis in several tissues and organs during aging.

Here, we defined this aging scenario in muscle tissues by demonstrating the remarkable increase in nitration levels of ECM‐bound HGF (Y198 and Y250) in rat lower hind limb muscle tissues (gastrocnemius, plantaris, soleus, EDL, and TA muscles) particularly in IIa/IIx fiber types. The finding was achieved by the successful generation of anti‐nitrated Y198, Y250‐HGF mAbs, which allowed the comparative direct‐immunofluorescence microscopy for serial cross‐sectional specimens from three age groups, young (2‐month‐old), adult (10‐month‐old), and old rats (20‐month‐old) (Figures 4, 6, and S6). A remarkable generation/accumulation of nitrated HGF seems to begin in adults (10‐month‐old), at which the footprint clusters of peroxynitrite formation (NO and O_2_^−^ radical generation) were observed in a dot‐like pattern around myofibers with particular prominence near nuclei between or beside fibers (indicated by yellow arrows; see Figure S7). Considering the previously documented satellite cell activation mechanism of the HGF liberation‐c‐met, the progression of nitration/dysfunction of extracellular HGF results in decreasing satellite cell functionality that includes cell activation and the subsequent sequential events (proliferation, differentiation/fusion, and self‐renewal of myogenic stem cells) and hence may have strong link to the notable decline in skeletal muscle regeneration efficiency (Domingues‐Faria et al., 2016; Jang et al., 2011; Yamakawa et al., 2020), although awaiting direct evidence to conclusively show that nitrated HGF is having a role in age‐related muscle atrophy and impaired regeneration. In this scenario, HGF synthesized/secreted from activated satellite cells, proliferating myoblasts (Sheehan et al., 2000; Tatsumi & Allen, 2004), and M2 macrophages (Sakaguchi et al., 2014) is out of focus. It is possible that newly secreted HGF may also undergo nitration and then accumulate preferentially in the ECM of fast IIx and IIa fibers by an unknown mechanism. Interestingly, nitration of 90‐kDa HGF (the inactive form) was exclusively detected in the satellite cell ECM of the old rats by western blotting using the anti‐nitrated Y198‐HGF antibody (Figure 4d), suggesting that activation of the HGF molecule itself (enzymatic cleavage of the 90‐kDa form by an HGF activator to form a heterodimer of α‐ and β‐chains) is suppressed, possibly by down‐regulation of HGF‐activator expression and/or activity; this could be an additional factor causing dysfunction of extracellular HGF and decreasing satellite cell functionality. In contrast, as shown in Figure S9 (*bars b and d*), satellite cells from old rats are activated by active HGF to a level comparable to those from adult in culture, indicating that satellite cells may maintain normal HGF‐c‐met signaling potential (including c‐met expression) in aging. These results imply that disorder of the satellite cell activation mechanism is an essential factor involved in age‐related muscle atrophy with impaired regeneration, and that a sufficient number of quiescent satellite cells (myogenic stem cell pool) is required to receive the appropriate activation signal to proliferate and maintain muscle homeostasis. Therefore, a key question is whether and how much the number of satellite cells that remains quiescent is able to increase due to the progression of HGF nitration and dysfunction for binding to the signaling‐receptor c‐met (thus blocking the activation signal) during aging. These ideas suggest the possibility that the known shift in the satellite cell compartment toward committed myogenic satellite cells and away from self‐renewing stem cells with aging is a functional counterpart of progressive HGF nitration and dysfunctional satellite cell activation in old age.

With particular emphasis on reported findings, extracellular HGF nitration/dysfunction takes place preferentially in fast IIa and IIx myofibers with aging, while slow and fast IIb fibers are faint or negative for this event in rat lower hind limb muscles examined even at 20 months old (Figures 5 and 6). This fiber type‐associated HGF nitration may result, in part, from the quantitative predominance of extracellular HGF in fast IIa and IIx fibers, with some exceptions for slow and fast IIb fibers (indicated by arrowheads in the Figs.) and rare IIb/IIx chimeric fibers generated by possible conversion from IIb to IIx in aged muscle (Larsson et al., 1993). Considering a paucity of fast IIb fibers in human muscle, ECM‐bound HGF nitration of fast IIa and IIx fibers may account for a generalized manifestation of age‐related muscle atrophy in humans, which is characterized by fast fiber‐preferential atrophy (slow fibers are much less affected) and impaired regeneration accompanied by up‐regulation of intramuscular fibrogenesis (fibrosis) and adipogenesis (fat infiltration) (Alway et al., 2014; Brack et al., 2007; Conboy & Rando, 2005; Evans & Lexell, 1995; Korhonen et al., 2006; Sousa‐Victor et al., 2021; Taylor et al., 2023). This important issue awaits human studies to further understand the relationship between muscle atrophy and HGF nitration/dysfunction in aging. Nevertheless, the present study in rats revealed that nitrated HGF accumulation (Y198 and Y250 residues in the c‐met binding region) in the ECM of the muscle fiber niche may be a hallmark of muscle aging, highlighting a possible significance of HGF nitration/dysfunction in age‐related muscle atrophy with impaired regeneration. This scenario was encouraged by preliminary immunohistochemistry for calf muscle of 20‐month‐old rats to show that protein expression of E3 ubiquitin ligases, atrogin‐1 and MuRF1 (well‐known markers of muscle atrophy), are positive predominantly in nitrated ECM‐bound HGF‐positive fibers (i.e., as anticipated in fast IIa and IIx fibers; see Figure S10). The finding potentially supports a hypothesis that diminished activity of satellite cells, or skeletal muscle stem cells, with age is a cause of sarcopenia (García‐Prat et al., 2013; Gopinath & Rando, 2008) along with the related experiments using Pax7^CreER^‐DTA mice to show that recruitment of satellite cells is obligatory for overload hypertrophy of myofibers in plantaris and EDL muscles (Egner et al., 2016). This hypothesis itself seems to be still controversial; Pax7^CreER^‐DTA genetic mouse model to deplete young adult muscle of satellite cells to a level sufficient to impair regeneration throughout the life of the animal revealed that, despite reduced regenerative capacity along with fibrosis, the life‐long reduction of satellite cells did not accelerate nor exacerbate sarcopenia as monitored by muscle size or fiber‐type composition in TA muscle (Fry et al., 2014) and satellite cells do not appear to be necessary for overload hypertrophy of fibers in plantaris muscle (McCarthy et al., 2011). The same research group also reported that Pax7‐positive satellite cell depletion does not affect diaphragm adaptations to voluntary wheel running in young or aged mice as revealed by diaphragm mean fiber cross‐sectional area and fiber‐type distribution (Murach et al., 2017). The discrepancy may, in part, arise from differences in the fiber‐type composition of muscles examined. Nevertheless, there are no reports that refute the idea that diminished activity of satellite cells leads to impaired muscle regeneration and fibrosis in aging. The present work provides a possible explanation for these age‐related phenotypes by showing that there is a progressive accumulation of extracellular HGF nitration that leads to dysfunctional activation of satellite cells, and occurs preferentially in fast IIa and IIx myofibers with aging.

One of the most distinctive features of aging in skeletal muscle is the reduced capability for regeneration. Increasing evidence suggests that age‐deficient regeneration is directly linked to the exhaustion of the satellite cell pool and progressive reduction in regenerative potential of satellite cells as well as the negative impact of age‐associated dys‐coordination of extrinsic factors on satellite cell regeneration activity (Brack & Rando, 2007; Brzeszczyńska et al., 2018; Gomes et al., 2017; Sousa‐Victor & Muñoz‐Cánoves, 2016), although there are conflicting reports on whether the number of satellite cells decreases with age or whether the decreased number contributes to fiber atrophy (Brack et al., 2005; Collins et al., 2007; Hikida, 2011). In muscle regeneration in young adults, satellite cells are exposed to a sequence of extrinsic factors from the immediate niche, while the orchestrated dynamic process is disturbed in muscle of old adults, resulting in impaired satellite cell activation and muscle regeneration (Alway et al., 2014; Bjornson et al., 2012; Conboy et al., 2003; Gopinath & Rando, 2008). Evidence of extrinsic factors influencing the regenerative defect of aged muscle came from historical, classical experiments showing that transplantation of aged muscle into young rat recipients leads to restoration of aged muscle mass, contractile strength, and regenerative capacity, although engrafting young muscle into aged rats regenerated no better than old muscle (Carlson & Faulkner, 1989). Notably, the age‐related nitration/dysfunction of ECM‐bound HGF found in this study, directly abolishes satellite cell activation and the subsequent sequential events, and thus may be a potent extrinsic element responsible for the age‐related disruption of the muscle homeostatic machinery. Intrinsic processes are also involved in skeletal muscle aging and might be altered in response to extrinsic stimuli including the peroxynitrite‐induced events and other oxidative stresses. In relation to this insight, loss of proteostasis and protein‐quality control are features of skeletal muscle aging that are tightly affected by redox disruption and accumulation of abnormal proteins (Douglas & Dillin, 2010; Obin et al., 1998; Powers et al., 2007). Exposure to mild oxidative stress was reported to increase protein turnover, while exposure to severe oxidative stress extensively aggregates abnormal proteins making them more resistant to proteolysis (Grune et al., 1995, 2003). The regulation of muscle proteolytic capacity during aging is still controversial; some studies demonstrated an age‐related increase (Altun et al., 2010; Clavel et al., 2006) while others reported a decrease (Strucksberg et al., 2010). This discrepancy appears to arise from differences in muscle fiber types (Fernando et al., 2019, 2020), which provides a potential background to account for the fast fiber‐preferential atrophy resulting from selective proteolytic degradation of fast‐fiber proteins (myofibrillar, sarcoplasmic, and stromal proteins) in human aging.

Finally, it is worth noting that the HGF/c‐met axis counteracts myocardial fibrosis (Gallo et al., 2015; Nakamura et al., 2005; Taniyama et al., 2000); thus, an additional negative effect of HGF nitration/dysfunction is the loss of its protective role as a potent antifibrotic factor; hence, HGF nitration/dysfunction is also expected to explain the progression of age‐related intramuscular fibrosis (see Figure S8D). This phenotype is one of the most distinctive features of aging in skeletal muscle and may be further up‐regulated by a potent fibrotic factor, FGF2, that is resistant to nitration by peroxynitrite under physiological conditions (Figure 3b). A similar scenario can be described for another important growth factor, TGF‐β. Satellite cells maintained in a quiescent (or dormant) state survive oxidative stress better than activated and proliferating cells (Yin et al., 2013). This may explain the excessive expression of TGF‐β in aged muscles (Carlson et al., 2009; Carlson & Conboy, 2007; Greene & Allen, 1991) that favors satellite cell quiescence to protect cells from oxidative stress (Rathbone et al., 2011). Recent studies demonstrated that the up‐regulation of TGF‐β activity in aged muscle is induced by a low plasma level of circulating α‐Klotho, an anti‐aging factor (Ohsawa et al., 2023) and that the age‐related decline in circulating α‐Klotho also disrupts the mitochondrial ultrastructure of satellite cells to increase accumulation of reactive oxygen species (ROS) in the organelles, thus contributing to survival of the cell from oxidative stress (Iijima et al., 2023). Notably, given the resistance of TGF‐β3 to nitration under the same conditions that induced HGF nitration (Figure 3d), satellite cells may be further directed to senescence by staying at the quiescence state (inhibition of activation) to resist age‐related oxidative stress, supporting an important discussion centered on progressive down‐regulation of the satellite cell activation during aging (and the subsequent proliferation, as a result) by the remarkable increase in nitration/dysfunction levels of ECM‐bound HGF.

Overall, findings extend our understanding of myogenic stem cell biology responsible for skeletal muscle homeostasis and thus, susceptibility to muscle atrophy with impaired regeneration in aging, supporting a hypothesis that diminished activity of satellite cells, or skeletal muscle stem cells, with age is a cause of sarcopenia (García‐Prat et al., 2013; Gopinath & Rando, 2008). The present work highlights the first comprehensive molecular implication of the extracellular HGF nitration/dysfunction in an age‐associated muscular disorder. The mechanism could, therefore, be applied to develop novel pharmaceutical strategies for preventing the HGF nitration (Y198 and Y250) in human/pet/animal health sciences to: combat age‐related muscle atrophy with impaired regeneration (including sarcopenia and frailty), guide the extension of healthy life expectancy, and importantly in improving animal welfare and sustaining food security through meat‐animal production. Again, it is important to note that muscle homeostasis requires HGF‐c‐met signaling being active just at the cell activation and proliferation (not differentiation) phases, as defined by c‐met expression restricted at quiescent and activation/proliferation phases of cells and also by the following excellent studies. First, the specific expression of an oncogenic version of c‐met in terminally differentiated skeletal muscle during fetal development and in young adult mice caused severe muscle wasting, with decreased fiber size and loss of MyHC protein. Concomitantly, the mRNA of ubiquitin ligases atrogin‐1/MAFbx‐1, MuRF1, and of the lysosomal protease cathepsin L (markers of skeletal muscle atrophy) was significantly increased (Crepaldi et al., 2007). Second, the oncogenic c‐met induction in differentiated myotube cultures showed the aberrant phenotypes including a reduced protein level of MyHC, increased phosphorylation of Erk1,2 MAPK, the aggregated nuclei (myosacs), and the collapse of elongated myotubes (Sala et al., 2015). Third, c‐met appears to be an early‐immediate gene (not inhibited by cycloheximide), the expression of which quite rapidly increases upon stretching muscle fibers (Wozniak & Anderson, 2007). Therefore, the cell stage‐preferential HGF‐c‐met signaling is an important element in the construction of the pharmaceutical strategy described above.

Several studies have demonstrated that HGF displays pleiotropic functions for regeneration and repair of a variety of tissues/organs (Desole et al., 2021; Honda et al., 1995; Kawaida et al., 1994; Nakamura, 1994; Ohmichi et al., 1996; Yun et al., 2012; Zhang et al., 2020; Zhao et al., 2022), by promoting cell migration, proliferation, survival (from apoptosis and necrosis), angiogenesis, neuritogenesis, myofiber‐type commitment, and anti‐fibrosis (Anderson et al., 2017; Matsumoto et al., 2014; Tatsumi et al., 2017; Tatsumi, Sankoda, et al., 2009; Wang et al., 2018). Thus, therapeutic applications of HGF have been tested in various diseases including liver cirrhosis, chronic renal failure, lung fibrosis, myocardial infarction, arteriosclerosis obliterans, amyotrophic lateral sclerosis (ALS, Lou Gehrig's disease), and acute spinal cord injury (Matsuda, 1997; Matsumoto, 2002; Matsumoto & Nakamura, 1998; Morishita et al., 2004; Powell et al., 2010; Sala & Crepaldi, 2011; Sanada et al., 2020; Takano et al., 2017; Ueki et al., 1999). Prevention of HGF nitration/dysfunction may be an important perspective to improve the therapeutic effects of investigational HGF‐drugs as well as their tissue‐specific delivery.

## AUTHOR CONTRIBUTIONS

R.T. conceived the project. A.E., N.I., H.K., T.N., S.O., Y.M., S.T., M.S., S.K., K.Z., and R.T. performed the in vitro experiments, and A.E., N.I., S.K., R.K., T.M., Y.M., N.D., and R.T. did the in vivo experiments. T.N. handled the accessible surface area calculation for tyrosine residues and their graphical presentation. S.S., W.M., N.D., T.S., and J.E.A. assisted with the immunofluorescence of muscle sections. A.E., N.I., H.K., T.N., Y.M., S.S., R.K., S.T., M.S., S.K., N.D., and R.T. analyzed the data. S.S., W.M., and R.T. generated anti‐nitroY198/Y250‐HGF mAbs, and A.E., N.I., S.S., W.M., R.K., T.M., Y.M., N.D., T.S., J.E.A., R.E.A., and R.T. discussed and interpreted in vivo results. M.N., T.S., K.S., J.E.A., and R.E.A. contributed reagents/materials/analysis tools and provided advice on project planning and technical help. S.S., W.M., M.N., J.E.A., R.E.A., and R.T. directed and supervised the project, and A.E., J.E.A., R.E.A., and R.T. wrote the manuscript.

## FUNDING INFORMATION

This work was supported by Grants‐in‐Aid for Challenging Exploratory Research, Scientific Research (B), and International Research Fellow from the Japan Society for the Promotion of Science (JSPS KAKENHI Grant Numbers JP26660218, JP21H02347, and JP22F22389, respectively) (all to R. Tatsumi). The research was also supported in part by grant funds from the SEI Group CSR Foundation, the Ito Foundation, and the Uehara Memorial Foundation (all to R. Tatsumi). A. Elgaabari was supported by the Egypt‐Japan Education Partnership (EJEP), a full Ph.D. scholarship from the Egyptian Ministry of Higher Education and JSPS Postdoctoral Fellowship for Research in Japan (Standard FY 2022; P22389) during the course of this research. The funders had no role in study design, data collection and analysis, decision to publish, or preparation of the manuscript, and did not provide support in the form of salaries for any authors.

## CONFLICT OF INTEREST STATEMENT

The authors declare that the research was conducted in the absence of any commercial or financial relationships that could be construed as a potential conflict of interest.

## Supporting information


Figure S1

Figure S2

Figure S3

Figure S4

Figure S5

Figure S6

Figure S7

Figure S8

Figure S9

Figure S10

Table S1

Data S1

Data S2

Data S3

Data S4

## Data Availability

Data generated and analyzed in this study are included in the paper and the Supporting Information (Figures [Link], [Link], [Link], [Link], [Link], [Link], [Link], [Link], [Link], [Link], Table S1, and legends), and original Data files (for Figures 1, 2, S1, S8, and S9) are also available to interested person from the corresponding author upon reasonable request. Un‐cropped WB‐images (Figures 2g and 4d) are provided in the online version.

## References

[acel14041-bib-0001] Abello, N. J.‐R. (2009). *Chemical labeling for the analysis of proteins, peptides and metabolites by mass spectrometry*. s.n., Ph.D. Thesis, University Groningen/UMCG research database. https://pure.rug.nl/ws/portalfiles/portal/2682693/00titlecon.pdf

[acel14041-bib-0002] Allen, R. E. , Sheehan, S. M. , Taylor, R. G. , Kendall, T. L. , & Rice, G. M. (1995). Hepatocyte growth factor activates quiescent skeletal muscle satellite cells in vitro. Journal of Cellular Physiology, 165(2), 307–312. 10.1002/jcp.1041650211 7593208

[acel14041-bib-0003] Allen, R. E. , Temm‐Grove, C. J. , Sheehan, S. M. , & Rice, G. (1997). Skeletal muscle satellite cell cultures. In Methods in cell biology (Vol. 52, Chap. 8, pp. 155–176). Elsevier. 10.1016/s0091-679x(08)60378-7 9379949

[acel14041-bib-0004] Altun, M. , Besche, H. C. , Overkleeft, H. S. , Piccirillo, R. , Edelmann, M. J. , Kessler, B. M. , Goldberg, A. L. , & Ulfhake, B. (2010). Muscle wasting in aged, sarcopenic rats is associated with enhanced activity of the ubiquitin proteasome pathway. Journal of Biological Chemistry, 285(51), 39597–39608. 10.1074/jbc.m110.129718 20940294 PMC3000941

[acel14041-bib-0005] Alway, S. E. , Myers, M. J. , & Mohamed, J. S. (2014). Regulation of satellite cell function in sarcopenia. Frontiers in Aging Neuroscience, 6, 246. 10.3389/fnagi.2014.00246 25295003 PMC4170136

[acel14041-bib-0006] Anderson, J. E. (2000). A role for nitric oxide in muscle repair: Nitric oxide‐mediated activation of muscle satellite cells. Molecular Biology of the Cell, 11(5), 1859–1874. 10.1091/mbc.11.5.1859 10793157 PMC14889

[acel14041-bib-0007] Anderson, J. E. (2006). The satellite cell as a companion in skeletal muscle plasticity: Currency, conveyance, clue, connector and colander. Journal of Experimental Biology, 209(12), 2276–2292. 10.1242/jeb.02088 16731804

[acel14041-bib-0008] Anderson, J. E. (2021). Key concepts in muscle regeneration: Muscle “cellular ecology” integrates a gestalt of cellular cross‐talk, motility, and activity to remodel structure and restore function. European Journal of Applied Physiology, 122(2), 273–300. 10.1007/s00421-021-04865-4 34928395 PMC8685813

[acel14041-bib-0009] Anderson, J. E. , Do, M.‐K. Q. , Daneshvar, N. , Suzuki, T. , Dort, J. , Mizunoya, W. , & Tatsumi, R. (2017). The role of semaphorin3A in myogenic regeneration and the formation of functional neuromuscular junctions on new fibres. Biological Reviews, 92(3), 1389–1405. 10.1111/brv.12286 27296513

[acel14041-bib-0010] Anderson, J. E. , & Pilipowicz, O. (2002). Activation of muscle satellite cells in single‐fiber cultures. Nitric Oxide, 7(1), 36–41. 10.1016/S1089-8603(02)00011-3 12175818

[acel14041-bib-0011] Aoi, W. , & Sakuma, K. (2011). Oxidative stress and skeletal muscle dysfunction with aging. Current Aging Science, 4(2), 101–109. 10.2174/1874609811104020101 21235498

[acel14041-bib-0012] Barreiro, E. , Gea, J. , Corominas, J. M. , & Hussain, S. N. A. (2003). Nitric oxide synthases and protein oxidation in the quadriceps femoris of patients with chronic obstructive pulmonary disease. American Journal of Respiratory Cell and Molecular Biology, 29(6), 771–778. 10.1165/rcmb.2003-0138oc 12816735

[acel14041-bib-0013] Bartesaghi, S. , & Radi, R. (2018). Fundamentals on the biochemistry of peroxynitrite and protein tyrosine nitration. Redox Biology, 14, 618–625. 10.1016/j.redox.2017.09.009 29154193 PMC5694970

[acel14041-bib-0014] Batthyány, C. , Souza, J. M. , Durán, R. , Cassina, A. , Cerveñansky, C. , & Radi, R. (2005). Time course and site(s) of cytochrome c tyrosine nitration by peroxynitrite. Biochemistry, 44(22), 8038–8046. 10.1021/bi0474620 15924423

[acel14041-bib-0015] Bischoff, R. (1990). Interaction between satellite cells and skeletal muscle fibers. Development, 109(4), 943–952. 10.1242/dev.109.4.943 2226207

[acel14041-bib-0016] Bischoff, R. , & Franzini‐Armstrong, C. (2004). Satellite and stem cells in muscle regeneration. In A. G. Engel & C. Franzini‐Armstrong (Eds.), Myology (Vol. 1, 3rd ed., pp. 66–86). McGraw‐Hill.

[acel14041-bib-0017] Bjornson, C. R. R. , Cheung, T. H. , Liu, L. , Tripathi, P. V. , Steeper, K. M. , & Rando, T. A. (2012). Notch signaling is necessary to maintain quiescence in adult muscle stem cells. Stem Cells, 30(2), 232–242. 10.1002/stem.773 22045613 PMC3384696

[acel14041-bib-0018] Bohle, D. , Sagan, E. , Koppenol, W. , & Kissner, R. (2004). Tetramethylammonium salts of superoxide and peroxynitrite. In J. R. Shapley (Ed.), (Ed.‐in‐Chief), Inorganic syntheses (Vol. 34, pp. 36–42). Wiley‐Interscience, John Wiley & Sons.

[acel14041-bib-0019] Boonen, K. J. M. , & Post, M. J. (2008). The muscle stem cell niche: Regulation of satellite cells during regeneration. Tissue Engineering Part B: Reviews, 14(4), 419–431. 10.1089/ten.teb.2008.0045 18817477

[acel14041-bib-0020] Brack, A. S. , Bildsoe, H. , & Hughes, S. M. (2005). Evidence that satellite cell decrement contributes to preferential decline in nuclear number from large fibres during murine age‐related muscle atrophy. Journal of Cell Science, 118(20), 4813–4821. 10.1242/jcs.02602 16219688

[acel14041-bib-0021] Brack, A. S. , Conboy, M. J. , Roy, S. , Lee, M. , Kuo, C. J. , Keller, C. , & Rando, T. A. (2007). Increased Wnt signaling during aging alters muscle stem cell fate and increases fibrosis. Science, 317(5839), 807–810. 10.1126/science.1144090 17690295

[acel14041-bib-0022] Brack, A. S. , & Rando, T. A. (2007). Intrinsic changes and extrinsic influences of myogenic stem cell function during aging. Stem Cell Reviews, 3(3), 226–237. 10.1007/s12015-007-9000-2 17917136

[acel14041-bib-0023] Brzeszczyńska, J. , Meyer, A. , McGregor, R. , Schilb, A. , Degen, S. , Tadini, V. , Johns, N. , Langen, R. , Schols, A. , Glass, D. J. , Roubenoff, R. , Ross, J. A. , Fearon, K. C. H. , Greig, C. A. , & Jacobi, C. (2018). Alterations in the in vitro and in vivo regulation of muscle regeneration in healthy ageing and the influence of sarcopenia. Journal of Cachexia, Sarcopenia and Muscle, 9(1), 93–105. 10.1002/jcsm.12252 29214748 PMC5803613

[acel14041-bib-0024] Carlson, B. M. , & Faulkner, J. A. (1989). Muscle transplantation between young and old rats: Age of host determines recovery. American Journal of Physiology‐Cell Physiology, 256(6), C1262–C1266. 10.1152/ajpcell.1989.256.6.c1262 2735398

[acel14041-bib-0025] Carlson, M. , & Conboy, I. (2007). Regulating the notch pathway in embryonic, adult and old stem cells. Current Opinion in Pharmacology, 7(3), 303–309. 10.1016/j.coph.2007.02.004 17475556

[acel14041-bib-0026] Carlson, M. E. , Conboy, M. J. , Hsu, M. , Barchas, L. , Jeong, J. , Agrawal, A. , Schaffer, D. V. , & Conboy, I. M. (2009). Relative roles of TGF‐β1 and Wnt in the systemic regulation and aging of satellite cell responses. Aging Cell, 8(6), 676–689. 10.1111/j.1474-9726.2009.00517.x 19732043 PMC2783265

[acel14041-bib-0027] Castegna, A. , Thongboonkerd, V. , Klein, J. B. , Lynn, B. , Markesbery, W. R. , & Butterfield, D. A. (2003). Proteomic identification of nitrated proteins in Alzheimer's disease brain. Journal of Neurochemistry, 85(6), 1394–1401. 10.1046/j.1471-4159.2003.01786.x 12787059

[acel14041-bib-0028] Chakravarti, B. , & Chakravarti, D. N. (2017). Protein tyrosine nitration: Role in aging. Current Aging Science, 10(4), 246–262. 10.2174/1874609810666170315112634 28302048

[acel14041-bib-0029] Chargé, S. B. P. , & Rudnicki, M. A. (2004). Cellular and molecular regulation of muscle regeneration. Physiological Reviews, 84(1), 209–238. 10.1152/physrev.00019.2003 14715915

[acel14041-bib-0030] Clavel, S. , Coldefy, A.‐S. , Kurkdjian, E. , Salles, J. , Margaritis, I. , & Derijard, B. (2006). Atrophy‐related ubiquitin ligases, atrogin‐1 and MuRF1 are up‐regulated in aged rat Tibialis Anterior muscle. Mechanisms of Ageing and Development, 127(10), 794–801. 10.1016/j.mad.2006.07.005 16949134

[acel14041-bib-0031] Collaborative Computational Project, Number 4 . (1994). The CCP4 suite: Programs for protein crystallography. Acta Crystallographica. Section D, Biological Crystallography, 50(5), 760–763. 10.1107/s0907444994003112 15299374

[acel14041-bib-0032] Collins, C. A. , Olsen, I. , Zammit, P. S. , Heslop, L. , Petrie, A. , Partridge, T. A. , & Morgan, J. E. (2005). Stem cell function, self‐renewal, and behavioral heterogeneity of cells from the adult muscle satellite cell niche. Cell, 122(2), 289–301. 10.1016/j.cell.2005.05.010 16051152

[acel14041-bib-0033] Collins, C. A. , Zammit, P. S. , Ruiz, A. P. , Morgan, J. E. , & Partridge, T. A. (2007). A population of myogenic stem cells that survives skeletal muscle aging. Stem Cells, 25(4), 885–894. 10.1634/stemcells.2006-0372 17218401

[acel14041-bib-0034] Conboy, I. M. , Conboy, M. J. , Smythe, G. M. , & Rando, T. A. (2003). Notch‐mediated restoration of regenerative potential to aged muscle. Science, 302(5650), 1575–1577. 10.1126/science.1087573 14645852

[acel14041-bib-0035] Conboy, I. M. , & Rando, T. A. (2005). Aging, stem cells and tissue regeneration: Lessons from muscle. Cell Cycle, 4(3), 407–410. 10.4161/cc.4.3.1518 15725724

[acel14041-bib-0036] Crepaldi, T. , Bersani, F. , Scuoppo, C. , Accornero, P. , Prunotto, C. , Taulli, R. , Forni, P. E. , Leo, C. , Chiarle, R. , & Griffiths, J. (2007). Conditional activation of MET in differentiated skeletal muscle induces atrophy. Journal of Biological Chemistry, 282(9), 6812–6822. 10.1074/jbc.M610916200 17194700

[acel14041-bib-0037] Daiber, A. , Daub, S. , Bachschmid, M. , Schildknecht, S. , Oelze, M. , Steven, S. , Schmidt, P. , Megner, A. , Wada, M. , Tanabe, T. , Münzel, T. , Bottari, S. , & Ullrich, V. (2013). Protein tyrosine nitration and thiol oxidation by peroxynitrite—Strategies to prevent these oxidative modifications. International Journal of Molecular Sciences, 14(4), 7542–7570. 10.3390/ijms14047542 23567270 PMC3645702

[acel14041-bib-0038] Desole, C. , Gallo, S. , Vitacolonna, A. , Montarolo, F. , Bertolotto, A. , Vivien, D. , Comoglio, P. , & Crepaldi, T. (2021). HGF and MET: From brain development to neurological disorders. Frontiers in Cell and Developmental Biology, 9, 683609. 10.3389/fcell.2021.683609 34179015 PMC8220160

[acel14041-bib-0039] Di Meo, S. , Reed, T. T. , Venditti, P. , & Victor, V. M. (2016). Role of ROS and RNS sources in physiological and pathological conditions. Oxidative Medicine and Cellular Longevity, 2016, 1–44. 10.1155/2016/1245049 PMC4960346 27478531

[acel14041-bib-0040] Domingues‐Faria, C. , Vasson, M.‐P. , Goncalves‐Mendes, N. , Boirie, Y. , & Walrand, S. (2016). Skeletal muscle regeneration and impact of aging and nutrition. Ageing Research Reviews, 26, 22–36. 10.1016/j.arr.2015.12.004 26690801

[acel14041-bib-0041] Dorrens, J. , & Rennie, M. J. (2003). Effects of ageing and human whole body and muscle protein turnover. Scandinavian Journal of Medicine & Science in Sports, 13(1), 26–33. 10.1034/j.1600-0838.2003.00306.x 12535314

[acel14041-bib-0042] Douglas, P. M. , & Dillin, A. (2010). Protein homeostasis and aging in neurodegeneration. Journal of Cell Biology, 190(5), 719–729. 10.1083/jcb.201005144 20819932 PMC2935559

[acel14041-bib-0044] Egner, I. M. , Bruusgaard, J. C. , & Gundersen, K. (2016). Satellite cell depletion prevents fiber hypertrophy in skeletal muscle. Development, 143(16), 2898–2906. 10.1242/dev.134411 27531949

[acel14041-bib-0045] Elgaabari, A. , Imatomi, N. , Kido, H. , Seki, M. , Tanaka, S. , Matsuyoshi, Y. , Nakashima, T. , Sawano, S. , Mizunoya, Y. , Suzuki, T. , Nakamura, M. , Anderson, J. E. , & Tatsumi, R. (2022). A pilot study on nitration/dysfunction of NK1 segment of myogenic stem cell activator HGF. Biochemistry and Biophysics Reports, 31, 101295. 10.1016/j.bbrep.2022.101295 35721345 PMC9198319

[acel14041-bib-0046] Evans, W. J. , & Lexell, J. (1995). Human aging, muscle mass, and fiber type composition. Journals of Gerontology Series A: Biological Sciences and Medical Sciences, 50A(Special issue 11), 11–16. 10.1093/gerona/50a.special_issue.11 7493202

[acel14041-bib-0047] Fernando, R. , Castro, J. P. , Flore, T. , Deubel, S. , Grune, T. , & Ott, C. (2020). Age‐related maintenance of the autophagy‐lysosomal system is dependent on skeletal muscle type. Oxidative Medicine and Cellular Longevity, 2020, 1–8. 10.1155/2020/4908162 PMC7396090 32774673

[acel14041-bib-0048] Fernando, R. , Drescher, C. , Deubel, S. , Jung, T. , Ost, M. , Klaus, S. , Grune, T. , & Castro, J. P. (2019). Low proteasomal activity in fast skeletal muscle fibers is not associated with increased age‐related oxidative damage. Experimental Gerontology, 117(3), 45–52. 10.1016/j.exger.2018.10.018 30367978

[acel14041-bib-0049] Fry, C. S. , Lee, J. D. , Mula, J. , Kirby, T. J. , Jackson, J. R. , Liu, F. , Yang, L. , Mendias, C. L. , Dupont‐Versteegden, E. E. , McCarthy, J. J. , & Peterson, C. A. (2014). Inducible depletion of satellite cells in adult, sedentary mice impairs muscle regenerative capacity without affecting sarcopenia. Nature Medicine, 21(1), 76–80. 10.1038/nm.3710 PMC4289085 25501907

[acel14041-bib-0050] Fugere, N. A. , Ferrington, D. A. , & Thompson, L. V. (2006). Protein nitration with aging in the rat semimembranosus and soleus muscles. Journals of Gerontology Series A: Biological Sciences and Medical Sciences, 61(8), 806–812. 10.1093/gerona/61.8.806 16912096 PMC2692712

[acel14041-bib-0051] Gallo, S. , Sala, V. , Gatti, S. , & Crepaldi, T. (2015). Cellular and molecular mechanisms of HGF/Met in the cardiovascular system. Clinical Science, 129(12), 1173–1193. 10.1042/cs20150502 26561593

[acel14041-bib-0052] García‐Prat, L. , Sousa‐Victor, P. , & Muñoz‐Cánoves, P. (2013). Functional dysregulation of stem cells during aging: A focus on skeletal muscle stem cells. FEBS Journal, 280(17), 4051–4062. 10.1111/febs.12221 23452120

[acel14041-bib-0053] Gochman, E. , Mahajna, J. , & Reznick, A. Z. (2011). NF‐κB activation by peroxynitrite through IκBα‐dependent phosphorylation versus nitration in colon cancer cells. Anticancer Research, 31(5), 1607–1617. https://ar.iiarjournals.org/content/31/5/1607 21617217

[acel14041-bib-0054] Gomes, M. J. , Martinez, P. F. , Pagan, L. U. , Damatto, R. L. , Mariano Cezar, M. D. , Ruiz Lima, A. R. , Okoshi, K. , & Okoshi, M. P. (2017). Skeletal muscle aging: Influence of oxidative stress and physical exercise. Oncotarget, 8(12), 20428–20440. 10.18632/oncotarget.14670 28099900 PMC5386774

[acel14041-bib-0055] Good, P. F. , Hsu, A. , Werner, P. , Perl, D. P. , & Olanow, C. W. (1998). Protein nitration in Parkinson's disease. Journal of Neuropathology & Experimental Neurology, 57(4), 338–342. 10.1097/00005072-199804000-00006 9600227

[acel14041-bib-0056] Gopinath, S. D. , & Rando, T. A. (2008). Stem cell review series: Aging of the skeletal muscle stem cell niche. Aging Cell, 7(4), 590–598. 10.1111/j.1474-9726.2008.00399.x 18462272

[acel14041-bib-0057] Greene, E. A. , & Allen, R. E. (1991). Growth factor regulation of bovine satellite cell growth in vitro. Journal of Animal Science, 69(1), 146–152. 10.2527/1991.691146x 2005007

[acel14041-bib-0058] Grune, T. , Merker, K. , Sandig, G. , & Davies, K. J. A. (2003). Selective degradation of oxidatively modified protein substrates by the proteasome. Biochemical and Biophysical Research Communications, 305(3), 709–718. 10.1016/s0006-291x(03)00809-x 12763051

[acel14041-bib-0059] Grune, T. , Reinheckel, T. , Joshi, M. , & Davies, K. J. A. (1995). Proteolysis in cultured liver epithelial cells during oxidative stress. Journal of Biological Chemistry, 270(5), 2344–2351. 10.1074/jbc.270.5.2344 7836468

[acel14041-bib-0060] Hara, M. , Tabata, K. , Suzuki, T. , Do, M.‐K. Q. , Mizunoya, W. , Nakamura, M. , Nishimura, S. , Tabata, S. , Ikeuchi, Y. , Sunagawa, K. , Anderson, J. E. , Allen, R. E. , & Tatsumi, R. (2012). Calcium influx through a possible coupling of cation channels impacts skeletal muscle satellite cell activation in response to mechanical stretch. American Journal of Physiology‐Cell Physiology, 302(12), C1741–C1750. 10.1152/ajpcell.00068.2012 22460715

[acel14041-bib-0061] He, Y. , Yu, Z. , & Chen, S. (2018). Alpha‐synuclein nitration and its implications in Parkinson's disease. ACS Chemical Neuroscience, 10(2), 777–782. 10.1021/acschemneuro.8b00288 30183251

[acel14041-bib-0062] Hikida, R. S. (2011). Aging changes in satellite cells and their functions. Current Aging Science, 4(3), 279–297. 10.2174/1874609811104030279 21529324

[acel14041-bib-0063] Honda, S. , Kagoshima, M. , Wanaka, A. , Tohyama, M. , Matsumoto, K. , & Nakamura, T. (1995). Localization and functional coupling of HGF and c‐Met/HGF receptor in rat brain: Implication as neurotrophic factor. Molecular Brain Research, 32(2), 197–210. 10.1016/0169-328x(95)00075-4 7500831

[acel14041-bib-0064] Horiguchi, T. , Uryu, K. , Giasson, B. I. , Ischiropoulos, H. , LightFoot, R. , Bellmann, C. , Richter‐Landsberg, C. , Lee, V. M. Y. , & Trojanowski, J. Q. (2003). Nitration of tau protein is linked to neurodegeneration in tauopathies. American Journal of Pathology, 163(3), 1021–1031. 10.1016/s0002-9440(10)63462-1 12937143 PMC1868254

[acel14041-bib-0065] Iijima, H. , Gilmer, G. , Wang, K. , Bean, A. C. , He, Y. , Lin, H. , Tang, W.‐Y. , Lamont, D. , Tai, C. , Ito, A. , Jones, J. J. , Evans, C. , & Ambrosio, F. (2023). Age‐related matrix stiffening epigenetically regulates α‐Klotho expression and compromises chondrocyte integrity. Nature Communications, 14(1), 18. 10.1038/s41467-022-35359-2 PMC9832042 36627269

[acel14041-bib-0066] Ischiropoulos, H. (1998). Biological tyrosine nitration: A pathophysiological function of nitric oxide and reactive oxygen species. Archives of Biochemistry and Biophysics, 356(1), 1–11. 10.1006/abbi.1998.0755 9681984

[acel14041-bib-0067] Ischiropoulos, H. (2003). Biological selectivity and functional aspects of protein tyrosine nitration. Biochemical and Biophysical Research Communications, 305(3), 776–783. 10.1016/s0006-291x(03)00814-3 12763060

[acel14041-bib-0068] Ischiropoulos, H. (2009). Protein tyrosine nitration—An update. Archives of Biochemistry and Biophysics, 484(2), 117–121. 10.1016/j.abb.2008.10.034 19007743

[acel14041-bib-0069] Jackson, M. J. , Pollock, N. , Staunton, C. A. , Stretton, C. , Vasilaki, A. , & McArdle, A. (2020). Oxidative stress in skeletal muscle: Unraveling the potential beneficial and deleterious roles of reactive oxygen species. In Oxidative Stress (pp. 713–733). Elsevier. 10.1016/b978-0-12-818606-0.00034-1

[acel14041-bib-0070] Jang, Y. C. , Sinha, M. , Cerletti, M. , Dall'Osso, C. , & Wagers, A. J. (2011). Skeletal muscle stem cells: Effects of aging and metabolism on muscle regenerative function. Cold Spring Harbor Symposia on Quantitative Biology, 2011(76), 101–111. 10.1101/sqb.2011.76.010652 21960527

[acel14041-bib-0071] Janssen, I. (2004). Skeletal muscle cutpoints associated with elevated physical disability risk in older men and women. American Journal of Epidemiology, 159(4), 413–421. 10.1093/aje/kwh058 14769646

[acel14041-bib-0072] Janssen, I. (2010). Evolution of sarcopenia research. Applied Physiology, Nutrition, and Metabolism, 35(5), 707–712. 10.1139/h10-067 20962927

[acel14041-bib-0073] Kann, A. P. , Hung, M. , Wang, W. , Nguyen, J. , Gilbert, P. M. , Wu, Z. , & Krauss, R. S. (2022). An injury‐responsive Rac‐to‐Rho GTPase switch drives activation of muscle stem cells through rapid cytoskeletal remodeling. Cell Stem Cell, 29(6), 933–947.e936. 10.1016/j.stem.2022.04.016 35597234 PMC9177759

[acel14041-bib-0074] Kawaida, K. , Matsumoto, K. , Shimazu, H. , & Nakamura, T. (1994). Hepatocyte growth factor prevents acute renal failure and accelerates renal regeneration in mice. Proceedings of the National Academy of Sciences of USA, 91(10), 4357–4361. 10.1073/pnas.91.10.4357 PMC43784 8183913

[acel14041-bib-0075] Korhonen, M. T. , Cristea, A. , Alén, M. , Häkkinen, K. , Sipilä, S. , Mero, A. , Viitasalo, J. T. , Larsson, L. , & Suominen, H. (2006). Aging, muscle fiber type, and contractile function in sprint‐trained athletes. Journal of Applied Physiology, 101(3), 906–917. 10.1152/japplphysiol.00299.2006 16690791

[acel14041-bib-0076] Larsson, L. , Biral, D. , Campione, M. , & Schiaffino, S. (1993). An age‐related type IIB to IIX myosin heavy chain switching in rat skeletal muscle. Acta Physiologica Scandinavica, 147(2), 227–234. 10.1111/j.1748-1716.1993.tb09493.x 8475750

[acel14041-bib-0077] Lokker, N. A. , Presta, L. G. , & Godowski, P. J. (1994). Mutational analysis and molecular modeling of the N‐terminal kringle‐containing domain of hepatocyte growth factor identifies amino acid side chains important for interaction with the c‐Met receptor. Protein Engineering, Design and Selection, 7(7), 895–903. 10.1093/protein/7.7.895 7971951

[acel14041-bib-0078] Ma, N. , Chen, D. , Lee, J.‐H. , Kuri, P. , Hernandez, E. B. , Kocan, J. , Mahmood, H. , Tichy, E. D. , Rompolas, P. , & Mourkioti, F. (2022). Piezo1 regulates the regenerative capacity of skeletal muscles via orchestration of stem cell morphological states. Science Advances, 8(11), eabn0485. 10.1126/sciadv.abn0485 35302846 PMC8932657

[acel14041-bib-0079] Markowitz, J. , Wang, J. , Vangundy, Z. , You, J. , Yildiz, V. , Yu, L. , Foote, I. P. , Branson, O. E. , Stiff, A. R. , Brooks, T. R. , Biesiadecki, B. , Olencki, T. , Tridandapani, S. , Freitas, M. A. , Papenfuss, T. , Phelps, M. A. , & Carson, W. E. (2017). Nitric oxide mediated inhibition of antigen presentation from DCs to CD4^+^ T cells in cancer and measurement of STAT1 nitration. Scientific Reports, 7, 15424. 10.1038/s41598-017-14970-0 29133913 PMC5684213

[acel14041-bib-0080] Martin‐Romero, F. J. , Gutiérrez‐Martin, Y. , Henao, F. , & Gutiérrez‐Merino, C. (2004). Fluorescence measurements of steady state peroxynitrite production upon SIN‐1 decomposition: NADH versus dihydrodichlorofluorescein and dihydrorhodamine 123. Journal of Fluorescence, 14(1), 17–23. 10.1023/b:jofl.0000014655.89256.bd 15622856

[acel14041-bib-0081] Matsuda, Y. (1997). Preventive and therapeutic effects in rats of hepatocyte growth factor infusion on liver fibrosis/cirrhosis. Hepatology, 26(1), 81–89. 10.1053/jhep.1997.v26.pm0009214455 9214455

[acel14041-bib-0082] Matsumoto, K. (2002). Renotropic role and therapeutic potential of HGF in the kidney. Nephrology Dialysis Transplantation, 17(suppl. 9), 59–61. 10.1093/ndt/17.suppl_9.59 12386291

[acel14041-bib-0083] Matsumoto, K. , Funakoshi, H. , Takahashi, H. , & Sakai, K. (2014). HGF‐Met pathway in regeneration and drug discovery. Biomedicine, 2(4), 275–300. 10.3390/biomedicines2040275 PMC5344275 28548072

[acel14041-bib-0084] Matsumoto, K. , & Nakamura, T. (1998). Plasminogen‐related growth factors. In Ciba foundation symposium, 212 (pp. 198–214). Wiley Online Library, John Wiley & Sons.

[acel14041-bib-0085] McCarthy, J. J. , Mula, J. , Miyazaki, M. , Erfani, R. , Garrison, K. , Farooqui, A. B. , Srikuea, R. , Lawson, B. A. , Grimes, B. , Keller, C. , Van Zant, G. , Campbell, K. S. , Esser, K. A. , Dupont‐Versteegden, E. E. , & Peterson, C. A. (2011). Effective fiber hypertrophy in satellite cell‐depleted skeletal muscle. Development, 138(17), 3657–3666. 10.1242/dev.068858 21828094 PMC3152923

[acel14041-bib-0086] McCormick, K. M. , & Schultz, E. (1994). Role of satellite cells in altering myosin expression during avian skeletal muscle hypertrophy. Developmental Dynamics, 199(1), 52–63. 10.1002/aja.1001990106 8167379

[acel14041-bib-0087] Mecocci, P. , Fanó, G. , Fulle, S. , MacGarvey, U. , Shinobu, L. , Polidori, M. C. , Cherubini, A. , Vecchiet, J. , Senin, U. , & Beal, M. F. (1999). Age‐dependent increases in oxidative damage to DNA, lipids, and proteins in human skeletal muscle. Free Radical Biology and Medicine, 26(3–4), 303–308. 10.1016/s0891-5849(98)00208-1 9895220

[acel14041-bib-0088] Mihm, M. (2001). Peroxynitrite induced nitration and inactivation of myofibrillar creatine kinase in experimental heart failure. Cardiovascular Research, 49(4), 798–807. 10.1016/s0008-6363(00)00307-2 11230979

[acel14041-bib-0089] Mittl, P. R. E. , Priestle, J. P. , Cox, D. A. , McMaster, G. , Cerletti, N. , & Grütter, M. G. (1996). The crystal structure of TGF‐β3 and comparison to TGF‐β2: Implications for receptor binding. Protein Science, 5(7), 1261–1271. 10.1002/pro.5560050705 8819159 PMC2143453

[acel14041-bib-0090] Montano, M. (2014). Translational biology in medicine: Models from aging, muscle regeneration and infection (1st ed.). Elsevier (Woodhead Publishing Series in *Biomedicine*, Number 71).

[acel14041-bib-0091] Morishita, R. , Aoki, M. , Hashiya, N. , Yamasaki, K. , Kurinami, H. , Shimizu, S. , Makino, H. , Takesya, Y. , Azuma, J. , & Ogihara, T. (2004). Therapeutic angiogenesis using hepatocyte growth factor (HGF). Current Gene Therapy, 4(2), 199–206. 10.2174/1566523043346453 15180586

[acel14041-bib-0092] Murach, K. A. , Confides, A. L. , Ho, A. , Jackson, J. R. , Ghazala, L. S. , Peterson, C. A. , & Dupont‐Versteegden, E. E. (2017). Depletion of Pax7^+^ satellite cells does not affect diaphragm adaptations to running in young or aged mice. Journal of Physiology, 595(19), 6299–6311. 10.1113/jp274611 28736900 PMC5621498

[acel14041-bib-0093] Nakamura, T. (1994). Hepatocyte growth factor as mitogen, motogen and morphogen, and its roles in organ regeneration. In Princess Takamatsu Symp. (pp. 195–213). University of Tokyo Press. 8983076

[acel14041-bib-0094] Nakamura, T. , Matsumoto, K. , Mizuno, S. , Sawa, Y. , Matsuda, H. , & Nakamura, T. (2005). Hepatocyte growth factor prevents tissue fibrosis, remodeling, and dysfunction in cardiomyopathic hamster hearts. American Journal of Physiology‐Heart and Circulatory Physiology, 288(5), H2131–H2139. 10.1152/ajpheart.01239.2003 15840903

[acel14041-bib-0095] Naldini, L. , Tamagnone, L. , Vigna, E. , Sachs, M. , Hartmann, G. , Birchmeier, W. , Daikuhara, Y. , Tsubouchi, H. , Blasi, F. , & Comoglio, P. M. (1992). Extracellular proteolytic cleavage by urokinase is required for activation of hepatocyte growth factor/scatter factor. EMBO Journal, 11(13), 4825–4833. 10.1002/j.1460-2075.1992.tb05588.x 1334458 PMC556958

[acel14041-bib-0096] Ng, J. Y. , Boelen, L. , & Wong, J. W. H. (2013). Bioinformatics analysis reveals biophysical and evolutionary insights into the 3‐nitrotyrosine post‐translational modification in the human proteome. Open Biology, 3(2), 120148. 10.1098/rsob.120148 23389939 PMC3603447

[acel14041-bib-0097] Obin, M. , Shang, F. , Gong, X. , Handelman, G. , Blumberg, J. , & Taylor, A. (1998). Redox regulation of ubiquitin‐conjugating enzymes: Mechanistic insights using the thiol‐specific oxidant diamide. FASEB Journal, 12(7), 561–569. 10.1096/fasebj.12.7.561 9576483

[acel14041-bib-0098] Ohmichi, H. , Matsumoto, K. , & Nakamura, T. (1996). In vivo mitogenic action of HGF on lung epithelial cells: Pulmotrophic role in lung regeneration. American Journal of Physiology‐Lung Cellular and Molecular Physiology, 270(6), L1031–L1039. 10.1152/ajplung.1996.270.6.l1031 8764230

[acel14041-bib-0099] Ohsawa, Y. , Ohtsubo, H. , Munekane, A. , Ohkubo, K. , Murakami, T. , Fujino, M. , Nishimatsu, S.‐i. , Hagiwara, H. , Nishimura, H. , Kaneko, R. , Suzuki, T. , Tatsumi, R. , Mizunoya, W. , Hinohara, A. , Fukunaga, M. , & Sunada, Y. (2023). Circulating α‐klotho counteracts TGF‐β‐induced sarcopenia. American Journal of Pathology, 193(5), 591–607. 10.1016/j.ajpath.2023.01.009 36773783

[acel14041-bib-0100] Pacher, P. , Beckman, J. S. , & Liaudet, L. (2007). Nitric oxide and peroxynitrite in health and disease. Physiological Reviews, 87(1), 315–424. 10.1152/physrev.00029.2006 17237348 PMC2248324

[acel14041-bib-0101] Pallafacchina, G. , François, S. , Regnault, B. , Czarny, B. , Dive, V. , Cumano, A. , Montarras, D. , & Buckingham, M. (2010). An adult tissue‐specific stem cell in its niche: A gene profiling analysis of in vivo quiescent and activated muscle satellite cells. Stem Cell Research, 4(2), 77–91. 10.1016/j.scr.2009.10.003 19962952

[acel14041-bib-0102] Pandya, C. D. , Lee, B. , Toque, H. A. , Mendhe, B. , Bragg, R. T. , Pandya, B. , Atawia, R. T. , Isales, C. , Hamrick, M. , Caldwell, R. W. , & Fulzele, S. (2019). Age‐dependent oxidative stress elevates arginase 1 and uncoupled nitric oxide synthesis in skeletal muscle of aged mice. Oxidative Medicine and Cellular Longevity, 2019, 1704650. 10.1155/2019/1704650 31205583 PMC6530149

[acel14041-bib-0103] Peixoto, Á. S. , Geyer, R. R. , Iqbal, A. , Truzzi, D. R. , Soares Moretti, A. I. , Laurindo, F. R. M. , & Augusto, O. (2018). Peroxynitrite preferentially oxidizes the dithiol redox motifs of protein‐disulfide isomerase. Journal of Biological Chemistry, 293(4), 1450–1465. 10.1074/jbc.m117.807016 29191937 PMC5787819

[acel14041-bib-0104] Perandini, L. A. , Chimin, P. , Lutkemeyer, D. d. S. , & Câmara, N. O. S. (2018). Chronic inflammation in skeletal muscle impairs satellite cells function during regeneration: Can physical exercise restore the satellite cell niche? FEBS Journal, 285(11), 1973–1984. 10.1111/febs.14417 29473995

[acel14041-bib-0105] Perrin, D. , & Koppenol, W. H. (2000). The quantitative oxidation of methionine to methionine sulfoxide by peroxynitrite. Archives of Biochemistry and Biophysics, 377(2), 266–272. 10.1006/abbi.2000.1787 10845703

[acel14041-bib-0106] Petruk, A. A. , Bartesaghi, S. , Trujillo, M. , Estrin, D. A. , Murgida, D. , Kalyanaraman, B. , Marti, M. A. , & Radi, R. (2012). Molecular basis of intramolecular electron transfer in proteins during radical‐mediated oxidations: Computer simulation studies in model tyrosine‐cysteine peptides in solution. Archives of Biochemistry and Biophysics, 525(1), 82–91. 10.1016/j.abb.2012.05.012 22640642 PMC3414218

[acel14041-bib-0107] Powell, R. J. , Goodney, P. , Mendelsohn, F. O. , Moen, E. K. , & Annex, B. H. (2010). Safety and efficacy of patient specific intramuscular injection of HGF plasmid gene therapy on limb perfusion and wound healing in patients with ischemic lower extremity ulceration: Results of the HGF‐0205 trial. Journal of Vascular Surgery, 52(6), 1525–1530. 10.1016/j.jvs.2010.07.044 21146749 PMC5292269

[acel14041-bib-0108] Powers, S. K. , Kavazis, A. N. , & McClung, J. M. (2007). Oxidative stress and disuse muscle atrophy. Journal of Applied Physiology, 102(6), 2389–2397. 10.1152/japplphysiol.01202.2006 17289908

[acel14041-bib-0109] Powers, S. K. , Morton, A. B. , Ahn, B. , & Smuder, A. J. (2016). Redox control of skeletal muscle atrophy. Free Radical Biology and Medicine, 98, 208–217. 10.1016/j.freeradbiomed.2016.02.021 26912035 PMC5006677

[acel14041-bib-0110] Radi, R. (2004). Nitric oxide, oxidants, and protein tyrosine nitration. Proceedings of the National Academy of Sciences of USA, 101(12), 4003–4008. 10.1073/pnas.0307446101 PMC384685 15020765

[acel14041-bib-0111] Radi, R. (2013). Peroxynitrite, a stealthy biological oxidant. Journal of Biological Chemistry, 288(37), 26464–26472. 10.1074/jbc.r113.472936 23861390 PMC3772193

[acel14041-bib-0112] Rathbone, C. R. , Yamanouchi, K. , Chen, X. K. , Nevoret‐Bell, C. J. , Rhoads, R. P. , & Allen, R. E. (2011). Effects of transforming growth factor‐beta (TGF‐β1) on satellite cell activation and survival during oxidative stress. Journal of Muscle Research and Cell Motility, 32(2), 99–109. 10.1007/s10974-011-9255-8 21823037

[acel14041-bib-0113] Recacha, R. , Mulloy, B. , & Gherardi, E. (2012). *Crystal structure of NK2 in complex with fractionated Heparin DP10*. RCSB PDB, 3SP8. 10.2210/pdb3sp8/pdb

[acel14041-bib-0114] Reyes, J. F. , Reynolds, M. R. , Horowitz, P. M. , Fu, Y. , Guillozet‐Bongaarts, A. L. , Berry, R. , & Binder, L. I. (2008). A possible link between astrocyte activation and tau nitration in Alzheimer's disease. Neurobiology of Disease, 31(2), 198–208. 10.1016/j.nbd.2008.04.005 18562203 PMC2766349

[acel14041-bib-0115] Sado, Y. , Kagawa, M. , Kishiro, Y. , Sugihara, K. , Naito, I. , Seyer, J. M. , Sugimoto, M. , Oohashi, T. , & Ninomiya, Y. (1995). Establishment by the rat lymph node method of epitope‐defined monoclonal antibodies recognizing the six different α chains of human type IV collagen. Histochemistry and Cell Biology, 104(4), 267–275. 10.1007/bf01464322 8548560

[acel14041-bib-0116] Sakaguchi, S. , Shono, J.‐I. , Suzuki, T. , Sawano, S. , Anderson, J. E. , Do, M.‐K. Q. , Ohtsubo, H. , Mizunoya, W. , Sato, Y. , Nakamura, M. , Furuse, M. , Yamada, K. , Ikeuchi, Y. , & Tatsumi, R. (2014). Implication of anti‐inflammatory macrophages in regenerative moto‐neuritogenesis: Promotion of myoblast migration and neural chemorepellent semaphorin 3A expression in injured muscle. International Journal of Biochemistry and Cell Biology, 54, 272–285. 10.1016/j.biocel.2014.05.032 24886696

[acel14041-bib-0117] Sala, V. , & Crepaldi, T. (2011). Novel therapy for myocardial infarction: Can HGF/Met be beneficial? Cellular and Molecular Life Sciences, 68(10), 1703–1717. 10.1007/s00018-011-0633-6 21327916 PMC11114731

[acel14041-bib-0118] Sala, V. , Gallo, S. , Gatti, S. , Vigna, E. , Ponzetto, A. , & Crepaldi, T. (2015). Anti‐differentiation effect of oncogenic met receptor in terminally‐differentiated myotubes. Biomedicine, 3(1), 124–137. 10.3390/biomedicines3010124 PMC5344230 28536403

[acel14041-bib-0119] Sanada, F. , Fujikawa, T. , Shibata, K. , Taniyama, Y. , Rakugi, H. , & Morishita, R. (2020). Therapeutic angiogenesis using HGF plasmid. Annals of Vascular Diseases, 13(2), 109–115. 10.3400/avd.ra.20-00035 32595785 PMC7315247

[acel14041-bib-0120] Sawano, S. , Komiya, Y. , Ichitsubo, R. , Ohkawa, Y. , Nakamura, M. , Tatsumi, R. , Ikeuchi, Y. , & Mizunoya, W. (2016). A one‐step immunostaining method to visualize rodent muscle fiber type within a single specimen. PLoS One, 11(11), e0166080. 10.1371/journal.pone.0166080 27814384 PMC5096669

[acel14041-bib-0121] Schüler, S. C. , Kirkpatrick, J. M. , Schmidt, M. , Santinha, D. , Koch, P. , Di Sanzo, S. , Cirri, E. , Hemberg, M. , Ori, A. , & von Maltzahn, J. (2021). Extensive remodeling of the extracellular matrix during aging contributes to age‐dependent impairments of muscle stem cell functionality. Cell Reports, 35(10), 109223. 10.1016/j.celrep.2021.109223 34107247

[acel14041-bib-0122] Schultz, E. , & McCormick, K. M. (1994). Skeletal muscle satellite cells. In Reviews of physiology, biochemistry and pharmacology (Vol. 123, pp. 213–257). Springer. 10.1007/bfb0030904 8209136

[acel14041-bib-0123] Sheehan, S. M. , Tatsumi, R. , Temm‐Grove, C. J. , & Allen, R. E. (2000). HGF is an autocrine growth factor for skeletal muscle satellite cells in vitro. Muscle & Nerve, 23(2), 239–245. 10639617 10.1002/(sici)1097-4598(200002)23:2<239::aid-mus15>3.0.co;2-u

[acel14041-bib-0124] Shishehbor, M. H. (2003). Association of nitrotyrosine levels with cardiovascular disease and modulation by statin therapy. JAMA, 289(13), 1675. 10.1001/jama.289.13.1675 12672736

[acel14041-bib-0125] Singh, S. , Canseco, D. C. , Manda, S. M. , Shelton, J. M. , Chirumamilla, R. R. , Goetsch, S. C. , Ye, Q. , Gerard, R. D. , Schneider, J. W. , Richardson, J. A. , Rothermel, B. A. , & Mammen, P. P. A. (2013). Cytoglobin modulates myogenic progenitor cell viability and muscle regeneration. Proceedings of the National Academy of Sciences of USA, 111(1), E129–E138. 10.1073/pnas.1314962111 PMC3890830 24367119

[acel14041-bib-0126] Siwanowicz, I. , Popowicz, G. M. , Wisniewska, M. , Huber, R. , Kuenkele, K.‐P. , Lang, K. , Engh, R. A. , & Holak, T. A. (2005). Structural basis for the regulation of insulin‐like growth factors by IGF binding proteins. Structure, 13(1), 155–167. 10.1016/j.str.2004.11.009 15642270

[acel14041-bib-0127] Sousa‐Victor, P. , García‐Prat, L. , & Muñoz‐Cánoves, P. (2021). Control of satellite cell function in muscle regeneration and its disruption in ageing. Nature Reviews Molecular Cell Biology, 23(3), 204–226. 10.1038/s41580-021-00421-2 34663964

[acel14041-bib-0128] Sousa‐Victor, P. , & Muñoz‐Cánoves, P. (2016). Regenerative decline of stem cells in sarcopenia. Molecular Aspects of Medicine, 50, 109–117. 10.1016/j.mam.2016.02.002 26921790

[acel14041-bib-0129] Souza, J. M. , Daikhin, E. , Yudkoff, M. , Raman, C. S. , & Ischiropoulos, H. (1999). Factors determining the selectivity of protein tyrosine nitration. Archives of Biochemistry and Biophysics, 371(2), 169–178. 10.1006/abbi.1999.1480 10545203

[acel14041-bib-0130] Strucksberg, K.‐H. , Tangavelou, K. , Schröder, R. , & Clemen, C. S. (2010). Proteasomal activity in skeletal muscle: A matter of assay design, muscle type, and age. Analytical Biochemistry, 399(2), 225–229. 10.1016/j.ab.2009.12.026 20034461

[acel14041-bib-0131] Szabó, C. , Ischiropoulos, H. , & Radi, R. (2007). Peroxynitrite: Biochemistry, pathophysiology and development of therapeutics. Nature Reviews Drug Discovery, 6(8), 662–680. 10.1038/nrd2222 17667957

[acel14041-bib-0132] Takano, M. , Kawabata, S. , Shibata, S. , Yasuda, A. , Nori, S. , Tsuji, O. , Nagoshi, N. , Iwanami, A. , Ebise, H. , Horiuchi, K. , Okano, H. , & Nakamura, M. (2017). Enhanced functional recovery from spinal cord injury in aged mice after stem cell transplantation through HGF induction. Stem Cell Reports, 8(3), 509–518. 10.1016/j.stemcr.2017.01.013 28216143 PMC5355635

[acel14041-bib-0133] Taniyama, Y. , Morishita, R. , Nakagami, H. , Moriguchi, A. , Sakonjo, H. , Shokei, K. , Matsumoto, K. , Nakamura, T. , Higaki, J. , & Ogihara, T. (2000). Potential contribution of a novel antifibrotic factor, hepatocyte growth factor, to prevention of myocardial fibrosis by angiotensin II blockade in cardiomyopathic hamsters. Circulation, 102(2), 246–252. 10.1161/01.cir.102.2.246 10889138

[acel14041-bib-0134] Tatsumi, R. (2010). Mechano‐biology of skeletal muscle hypertrophy and regeneration: Possible mechanism of stretch‐induced activation of resident myogenic stem cells. Animal Science Journal, 81(1), 11–20. 10.1111/j.1740-0929.2009.00712.x 20163667

[acel14041-bib-0135] Tatsumi, R. , & Allen, R. E. (2004). Active hepatocyte growth factor is present in skeletal muscle extracellular matrix. Muscle & Nerve, 30(5), 654–658. 10.1002/mus.20114 15389661

[acel14041-bib-0136] Tatsumi, R. , & Allen, R. E. (2008). Mechano‐biology of resident myogenic stem cells: Molecular mechanism of stretch‐induced activation of satellite cells. Animal Science Journal, 79(3), 279–290. 10.1111/j.1740-0929.2009.00712.x 20163667

[acel14041-bib-0137] Tatsumi, R. , Anderson, J. E. , Nevoret, C. J. , Halevy, O. , & Allen, R. E. (1998). HGF/SF is present in normal adult skeletal muscle and is capable of activating satellite cells. Developmental Biology, 194(1), 114–128. 10.1006/dbio.1997.8803 9473336

[acel14041-bib-0138] Tatsumi, R. , Hattori, A. , Ikeuchi, Y. , Anderson, J. E. , & Allen, R. E. (2002). Release of hepatocyte growth factor from mechanically stretched skeletal muscle satellite cells and role of pH and nitric oxide. Molecular Biology of the Cell, 13(8), 2909–2918. 10.1091/mbc.e02-01-0062 12181355 PMC117951

[acel14041-bib-0139] Tatsumi, R. , Liu, X. , Pulido, A. , Morales, M. , Sakata, T. , Dial, S. , Hattori, A. , Ikeuchi, Y. , & Allen, R. E. (2006). Satellite cell activation in stretched skeletal muscle and the role of nitric oxide and hepatocyte growth factor. American Journal of Physiology‐Cell Physiology, 290(6), C1487–C1494. 10.1152/ajpcell.00513.2005 16684931

[acel14041-bib-0140] Tatsumi, R. , Sankoda, Y. , Anderson, J. E. , Sato, Y. , Mizunoya, W. , Shimizu, N. , Suzuki, T. , Yamada, M. , Rhoads, R. P. , Ikeuchi, Y. , & Allen, R. E. (2009). Possible implication of satellite cells in regenerative motoneuritogenesis: HGF upregulates neural chemorepellent Sema3A during myogenic differentiation. American Journal of Physiology‐Cell Physiology, 297(2), C238–C252. 10.1152/ajpcell.00161.2009 19515904

[acel14041-bib-0141] Tatsumi, R. , Sheehan, S. M. , Iwasaki, H. , Hattori, A. , & Allen, R. E. (2001). Mechanical stretch induces activation of skeletal muscle satellite cells in vitro. Experimental Cell Research, 267(1), 107–114. 10.1006/excr.2001.5252 11412043

[acel14041-bib-0142] Tatsumi, R. , Suzuki, T. , Do, M.‐K. Q. , Ohya, Y. , Anderson, J. E. , Shibata, A. , Kawaguchi, M. , Ohya, S. , Ohtsubo, H. , Mizunoya, W. , Sawano, S. , Komiya, Y. , Ichitsubo, R. , Ojima, K. , Nishimatsu, S.‐I. , Nohno, T. , Ohsawa, Y. , Sunada, Y. , Nakamura, M. , … Allen, R. E. (2017). Slow‐myofiber commitment by semaphorin 3A secreted from myogenic stem cells. Stem Cells, 35(7), 1815–1834. 10.1002/stem.2639 28480592

[acel14041-bib-0143] Tatsumi, R. , Wuollet, A. L. , Tabata, K. , Nishimura, S. , Tabata, S. , Mizunoya, W. , Ikeuchi, Y. , & Allen, R. E. (2009). A role for calcium‐calmodulin in regulating nitric oxide production during skeletal muscle satellite cell activation. American Journal of Physiology‐Cell Physiology, 296(4), C922–C929. 10.1152/ajpcell.00471.2008 19158401

[acel14041-bib-0144] Tatsumi, R. , Yamada, M. , Katsuki, Y. , Okamoto, S. , Ishizaki, J. , Mizunoya, W. , Ikeuchi, Y. , Hattori, A. , Shimokawa, H. , & Allen, R. E. (2006). Low‐pH preparation of skeletal muscle satellite cells can be used to study activation in vitro. International Journal of Biochemistry & Cell Biology, 38(10), 1678–1685. 10.1016/j.biocel.2006.04.003 16750930

[acel14041-bib-0145] Taylor, J. A. , Greenhaff, P. L. , Bartlett, D. B. , Jackson, T. A. , Duggal, N. A. , & Lord, J. M. (2023). Multisystem physiological perspective of human frailty and its modulation by physical activity. Physiological Reviews, 103(2), 1137–1191. 10.1152/physrev.00037.2021 36239451 PMC9886361

[acel14041-bib-0146] Tidball, J. G. , Lavergne, E. , Lau, K. S. , Spencer, M. J. , Stull, J. T. , & Wehling, M. (1998). Mechanical loading regulates NOS expression and activity in developing and adult skeletal muscle. American Journal of Physiology‐Cell Physiology, 275(1), C260–C266. 10.1152/ajpcell.1998.275.1.c260 9688857

[acel14041-bib-0147] Tolbert, W. D. , Daugherty‐Holtrop, J. , Gherardi, E. , Vande Woude, G. , & Xu, H. E. (2010). Structural basis for agonism and antagonism of hepatocyte growth factor. Proceedings of the National Academy of Sciences of USA, 107(30), 13264–13269. 10.1073/pnas.1005183107 PMC2922134 20624990

[acel14041-bib-0148] Uchikawa, E. , Chen, Z. , Xiao, G.‐Y. , Zhang, X. , & Bai, X.‐c. (2021). Structural basis of the activation of c‐MET receptor. Nature Communications, 12, 1–14. 10.1038/s41467-021-24367-3 PMC8249616 34210960

[acel14041-bib-0149] Ueki, T. , Kaneda, Y. , Tsutsui, H. , Nakanishi, K. , Sawa, Y. , Morishita, R. , Matsumoto, K. , Nakamura, T. , Takahashi, H. , Okamoto, E. , & Fujimoto, J. (1999). Hepatocyte growth factor gene therapy of liver cirrhosis in rats. Nature Medicine, 5(2), 226–230. 10.1038/5593 9930873

[acel14041-bib-0150] Viner, R. I. , Ferrington, D. A. , Hühmer, A. F. R. , Bigelow, D. J. , & Schöneich, C. (1996). Accumulation of nitrotyrosine on the SERCA2a isoform of SR Ca‐ATPase of rat skeletal muscle during aging: A peroxynitrite‐mediated process? FEBS Letters, 379(3), 286–290. 10.1016/0014-5793(95)01530-2 8603707

[acel14041-bib-0151] Wang, Y. , Song, J. , Liu, X. , Liu, J. , Zhang, Q. , Yan, X. , Yuan, X. , & Ren, D. (2020). Multiple effects of mechanical stretch on myogenic progenitor cells. Stem Cells and Development, 29(6), 336–352. 10.1089/scd.2019.0286 31950873

[acel14041-bib-0152] Wang, Z. , Fei, S. , Suo, C. , Han, Z. , Tao, J. , Xu, Z. , Zhao, C. , Tan, R. , & Gu, M. (2018). Antifibrotic effects of hepatocyte growth factor on endothelial‐to‐mesenchymal transition via transforming growth factor‐beta1 (TGF‐β1)/Smad and Akt/mTOR/P70S6K signaling pathways. Annals of Transplantation, 23, 1–10. 10.12659/aot.906700 29292365 PMC6248046

[acel14041-bib-0153] Watanabe, K. , Chirgadze, D. Y. , Lietha, D. , Gherardi, E. , & Blundell, T. L. (2001). A new crystal form of the NK1 splice variant of HGF/SF demonstrates extensive hinge movement and suggests that the NK1 dimer originates by domain swapping. Journal of Molecular Biology, 319, 283–288. 10.1016/S0022-2836(02)00199-7 12051906

[acel14041-bib-0154] Wosczyna, M. N. , & Rando, T. A. (2018). A muscle stem cell support group: Coordinated cellular responses in muscle regeneration. Developmental Cell, 46(2), 135–143. 10.1016/j.devcel.2018.06.018 30016618 PMC6075730

[acel14041-bib-0155] Wozniak, A. C. , & Anderson, J. E. (2007). Nitric oxide‐dependence of satellite stem cell activation and quiescence on normal skeletal muscle fibers. Developmental Dynamics, 236(1), 240–250. 10.1002/dvdy.21012 17117435

[acel14041-bib-0156] Wozniak, A. C. , & Anderson, J. E. (2009). The dynamics of the nitric oxide release‐transient from stretched muscle cells. International Journal of Biochemistry & Cell Biology, 41(3), 625–631. 10.1016/j.biocel.2008.07.005 18694846

[acel14041-bib-0157] Wozniak, A. C. , Kong, J. , Bock, E. , Pilipowicz, O. , & Anderson, J. E. (2005). Signaling satellite‐cell activation in skeletal muscle: Markers, models, stretch, and potential alternate pathways. Muscle & Nerve, 31(3), 283–300. 10.1002/mus.20263 15627266

[acel14041-bib-0158] Yamada, M. , Sankoda, Y. , Tatsumi, R. , Mizunoya, W. , Ikeuchi, Y. , Sunagawa, K. , & Allen, R. E. (2008). Matrix metalloproteinase‐2 mediates stretch‐induced activation of skeletal muscle satellite cells in a nitric oxide‐dependent manner. International Journal of Biochemistry & Cell Biology, 40(10), 2183–2191. 10.1016/j.biocel.2008.02.017 18403250

[acel14041-bib-0159] Yamada, M. , Tatsumi, R. , Kikuiri, T. , Okamoto, S. , Nonoshita, S. , Mizunoya, W. , Ikeuchi, Y. , Shimokawa, H. , Sunagawa, K. , & Allen, R. E. (2006). Matrix metalloproteinases are involved in mechanical stretch‐induced activation of skeletal muscle satellite cells. Muscle & Nerve, 34(3), 313–319. 10.1002/mus.20601 16810685

[acel14041-bib-0160] Yamada, M. , Tatsumi, R. , Yamanouchi, K. , Hosoyama, T. , Shiratsuchi, S. , Sato, A. , Mizunoya, W. , Ikeuchi, Y. , Furuse, M. , & Allen, R. E. (2010). High concentrations of HGF inhibit skeletal muscle satellite cell proliferation in vitro by inducing expression of myostatin: A possible mechanism for reestablishing satellite cell quiescence in vivo. American Journal of Physiology‐Cell Physiology, 298(3), C465–C476. 10.1152/ajpcell.00449.2009 20007454 PMC2838568

[acel14041-bib-0161] Yamakawa, H. , Kusumoto, D. , Hashimoto, H. , & Yuasa, S. (2020). Stem cell aging in skeletal muscle regeneration and disease. International Journal of Molecular Sciences, 21(5), 1830. 10.3390/ijms21051830 32155842 PMC7084237

[acel14041-bib-0162] Yamakura, F. , Matsumoto, T. , Fujimura, T. , Taka, H. , Murayama, K. , Imai, T. , & Uchida, K. (2001). Modification of a single tryptophan residue in human Cu, Zn‐superoxide dismutase by peroxynitrite in the presence of bicarbonate. Biochimica et Biophysica Acta‐Protein Structure and Molecular Enzymology, 1548(1), 38–46. 10.1016/s0167-4838(01)00212-6 11451436

[acel14041-bib-0163] Yamakura, F. , Matsumoto, T. , Ikeda, K. , Taka, H. , Fujimura, T. , Murayama, K. , Watanabe, E. , Tamaki, M. , Imai, T. , & Takamori, K. (2005). Nitrated and oxidized products of a single tryptophan residue in human Cu, Zn‐superoxide dismutase treated with either peroxynitrite‐carbon dioxide or myeloperoxidase‐hydrogen peroxide‐nitrite. Journal of Biochemistry, 138(1), 57–69. 10.1093/jb/mvi095 16046449

[acel14041-bib-0164] Yin, H. , Price, F. , & Rudnicki, M. A. (2013). Satellite cells and the muscle stem cell niche. Physiological Reviews, 93(1), 23–67. 10.1152/physrev.00043.2011 23303905 PMC4073943

[acel14041-bib-0165] Yun, Y.‐R. , Jang, J. H. , Jeon, E. , Kang, W. , Lee, S. , Won, J.‐E. , Kim, H. W. , & Wall, I. (2012). Administration of growth factors for bone regeneration. Regenerative Medicine, 7(3), 369–385. 10.2217/rme.12.1 22594329

[acel14041-bib-0166] Zhan, X. , Huang, Y. , & Qian, S. (2018). Protein tyrosine nitration in lung cancer: Current research status and future perspectives. Current Medicinal Chemistry, 25(29), 3435–3454. 10.2174/0929867325666180221140745 29473494

[acel14041-bib-0167] Zhang, X.‐j. , Olsavszky, V. , Yin, Y. , Wang, B. , Engleitner, T. , Öllinger, R. , Schledzewski, K. , Koch, P.‐S. , Rad, R. , Schmid, R. M. , Friess, H. , Goerdt, S. , Hüser, N. , Géraud, C. , von Figura, G. , & Hartmann, D. (2020). Angiocrine hepatocyte growth factor signaling controls physiological organ and body size and dynamic hepatocyte proliferation to prevent liver damage during regeneration. American Journal of Pathology, 190(2), 358–371. 10.1016/j.ajpath.2019.10.009 31783007

[acel14041-bib-0168] Zhao, Y. , Ye, W. , Wang, Y.‐D. , & Chen, W.‐D. (2022). HGF/c‐met: A key promoter in liver regeneration. Frontiers in Pharmacology, 13, 808855. 10.3389/fphar.2022.808855 35370682 PMC8968572

[acel14041-bib-0169] Zhu, X. , Komiya, H. , Chirino, A. , Faham, S. , Fox, G. M. , Arakawa, T. , Hsu, B. T. , & Rees, D. C. (1991). Three‐dimensional structures of acidic and basic fibroblast growth factors. Science, 251(4989), 90–93. 10.1126/science.1702556 1702556

