# Supplementary figures and images for: Age‐related nitration/dysfunction of myogenic stem cell activator HGF
Source: Aging Cell. 2023 Nov 20;23(2):e14041. doi: 10.1111/acel.14041 (PMC10861216; doi:10.1111/acel.14041)

## Supplemental Materials

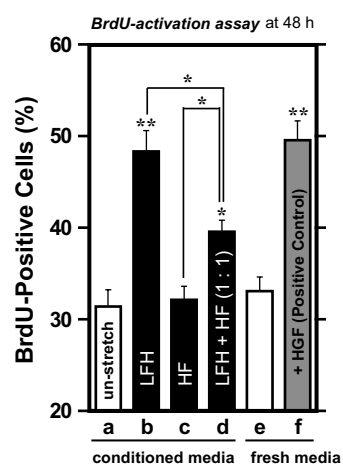

**Fig. S1, Elgaabari *et al.***  
(Supplemental to Fig. 1 D-F)

Supplement: Supplementary file 1 — Figure S1 [file ACEL-23-e14041-s007.pdf]

## Supplemental Materials

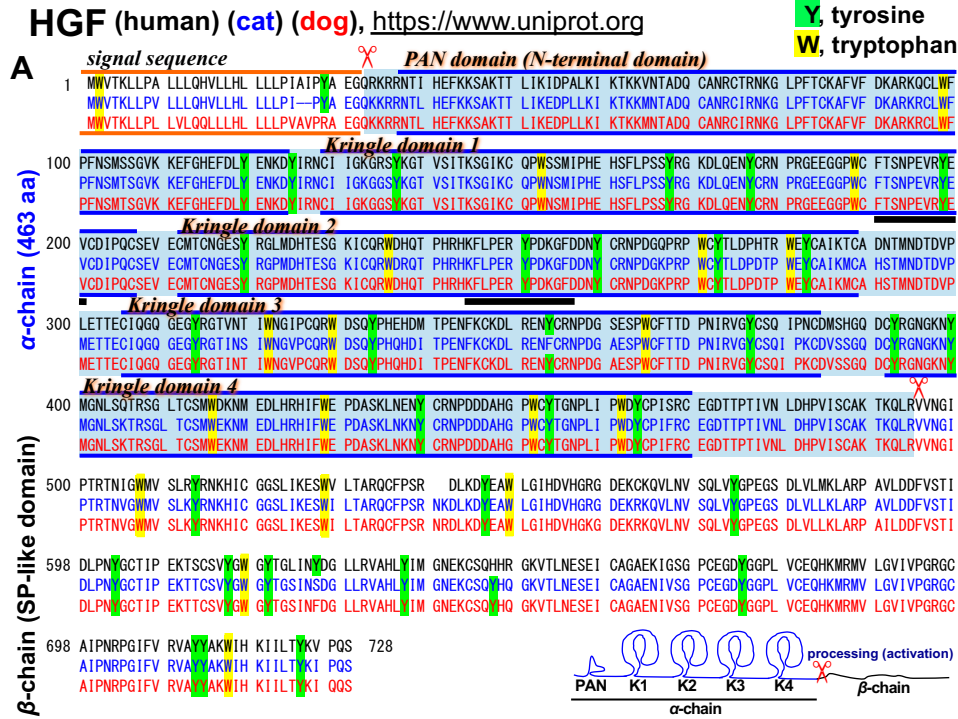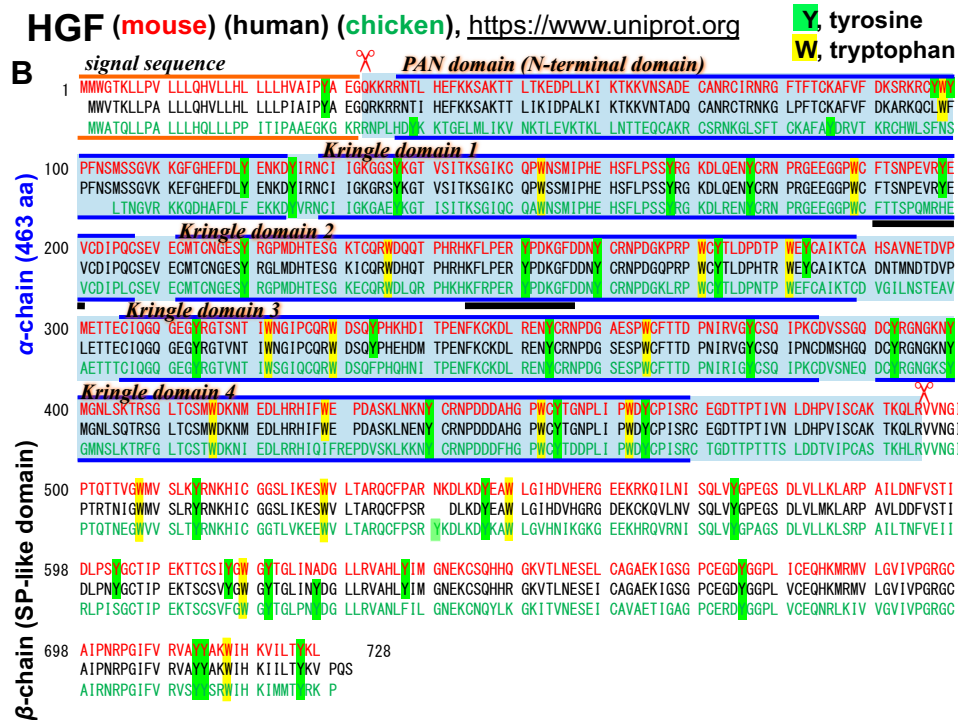

**Fig. S2 A,B, Elgaabari et al.**  
(Supplemental to Fig. 3 A)

Supplement: Supplementary file 2 — Figure S2 [file ACEL-23-e14041-s005.zip › Fig.S2AB.pdf]

Supplemental Materials

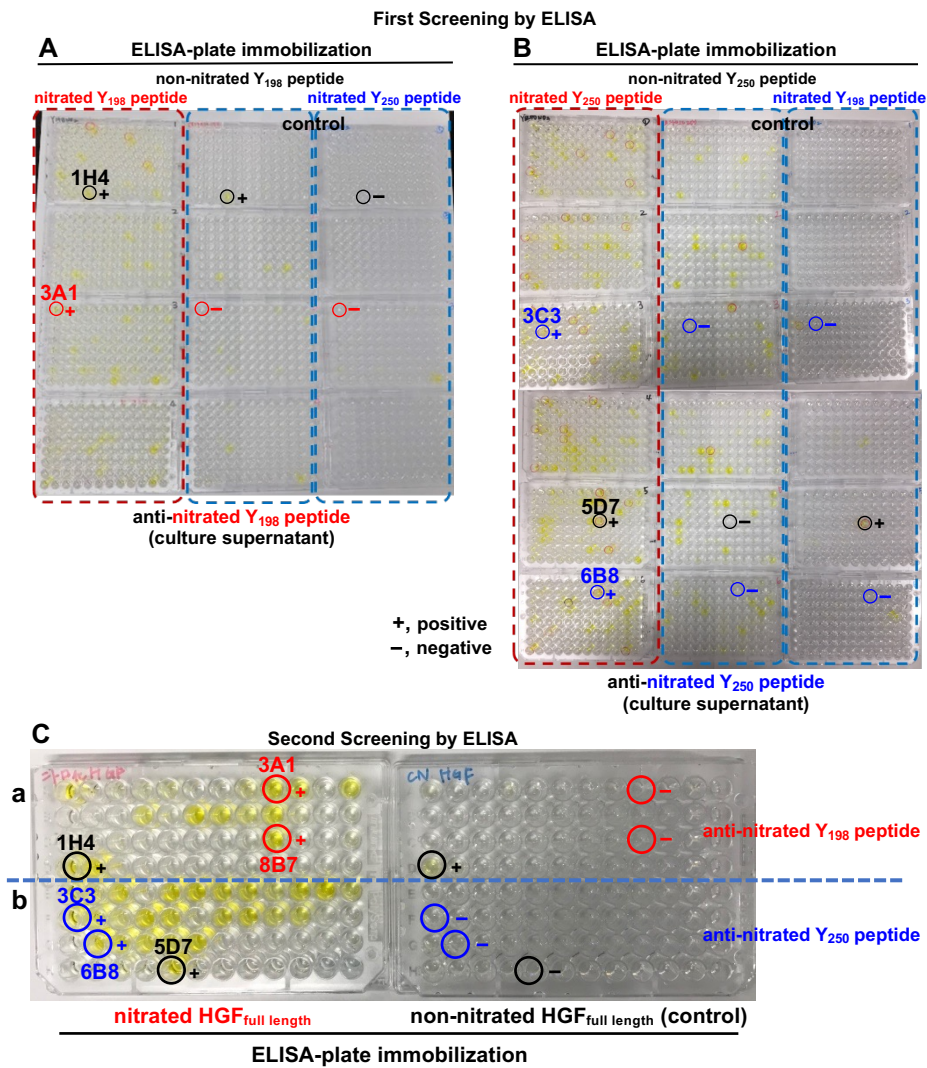

Fig. S4, Elgaabari *et al.*

Supplement: Supplementary file 4 — Figure S4 [file ACEL-23-e14041-s001.pdf]

## Supplemental Materials

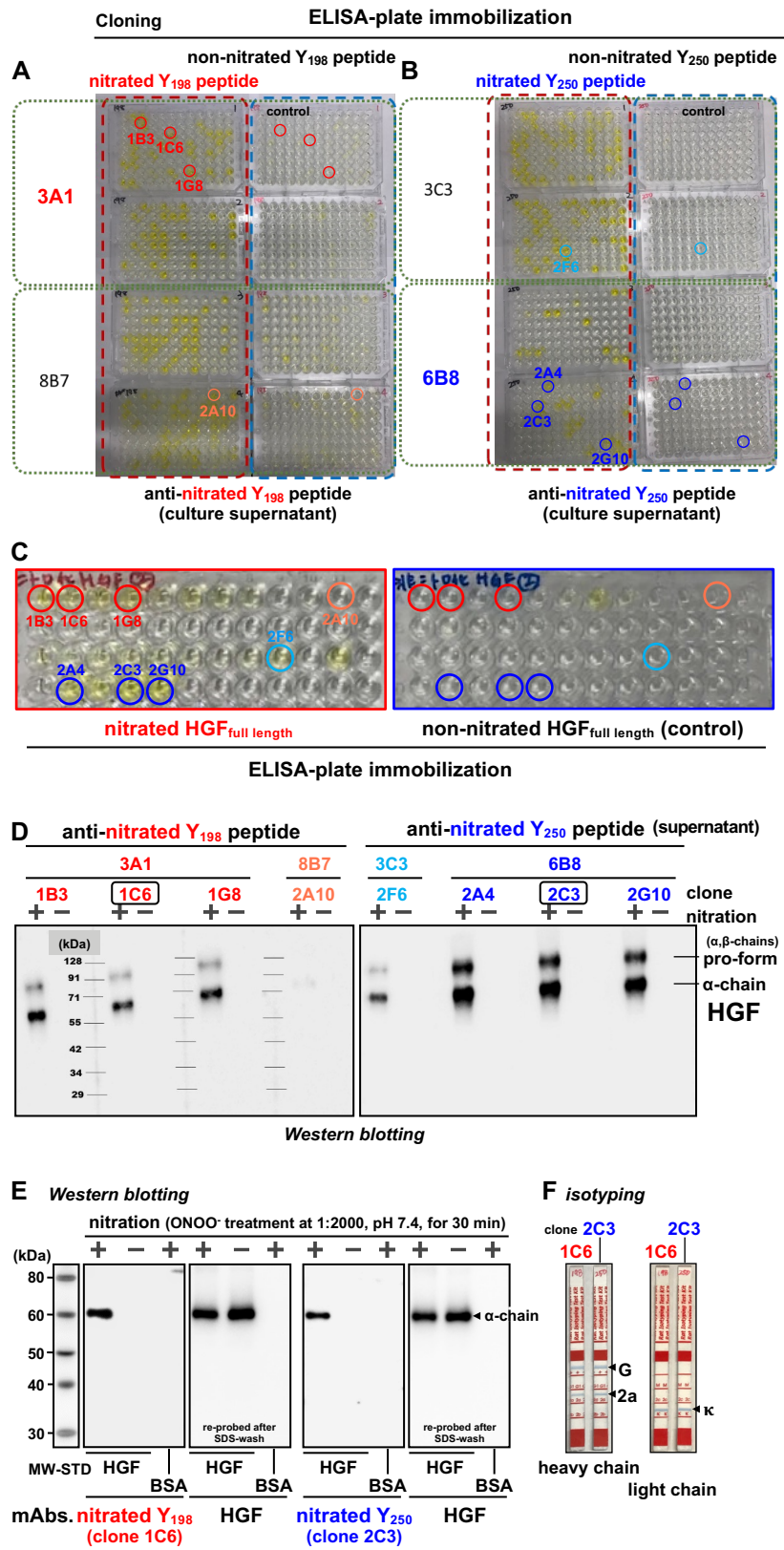

**Fig. S5, Elgaabari *et al.***

Supplement: Supplementary file 5 — Figure S5 [file ACEL-23-e14041-s012.pdf]

## Supplemental Materials

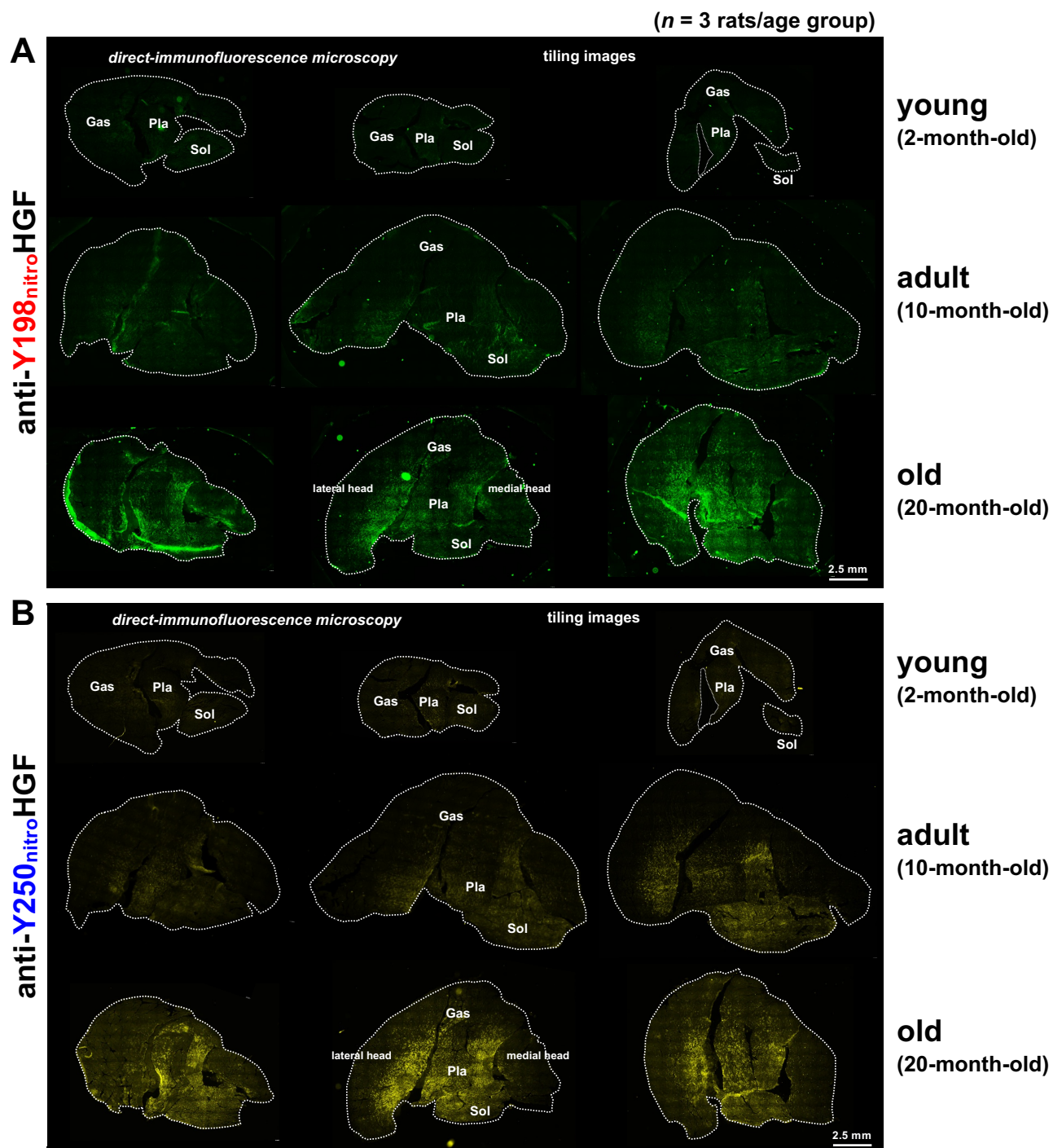

**Fig. S6, Elgaabari *et al.***  
(Supplemental to Fig. 4 A,B)

Supplement: Supplementary file 6 — Figure S6 [file ACEL-23-e14041-s013.pdf]

Supplemental Materials

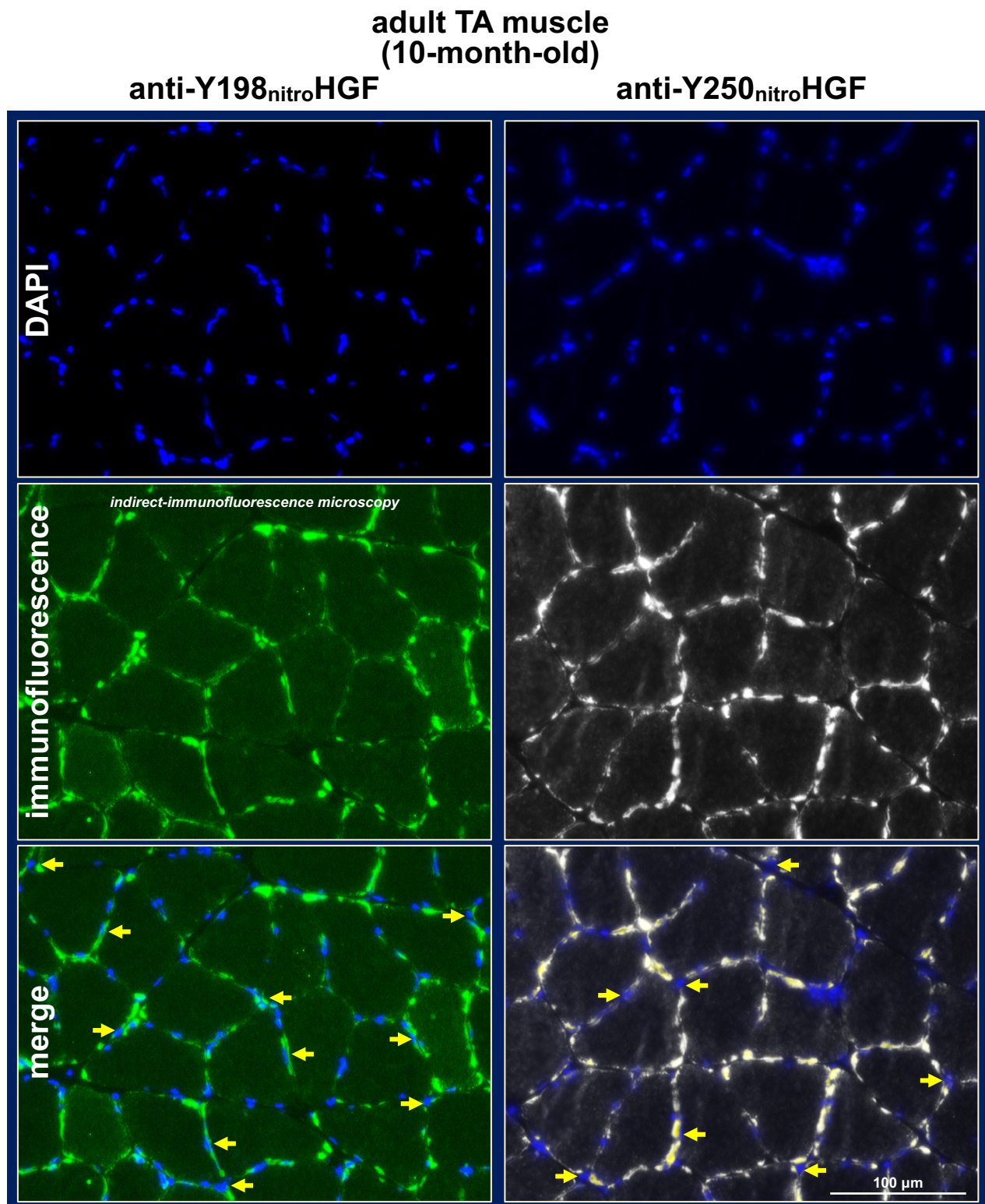

Fig. S7, Elgaabari *et al.*  
(Supplemental to Figs. 4-6)

Supplement: Supplementary file 7 — Figure S7 [file ACEL-23-e14041-s004.pdf]

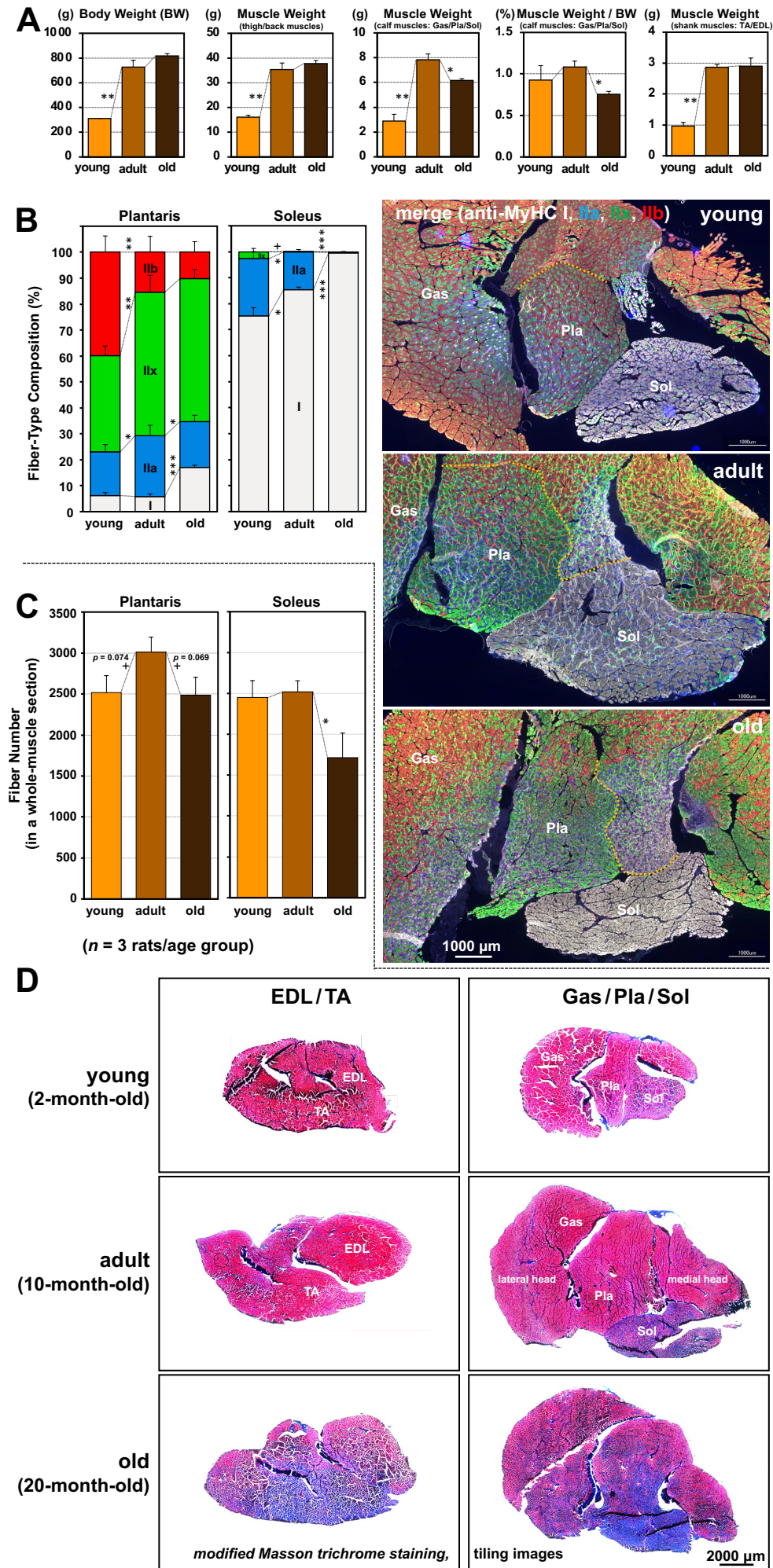

Fig. S8, Elgaabari et al.

Supplement: Supplementary file 8 — Figure S8 [file ACEL-23-e14041-s010.pdf]

## Supplemental Materials

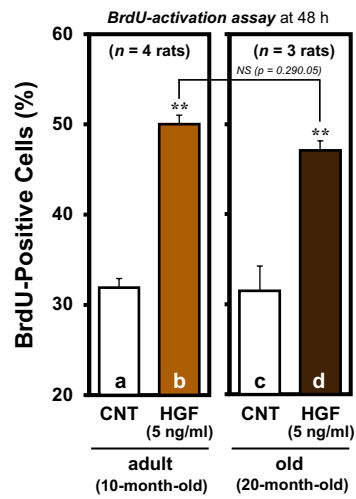

**Fig. S9, Elgaabari *et al.***

Supplement: Supplementary file 9 — Figure S9 [file ACEL-23-e14041-s006.pdf]

## Supplemental Materials

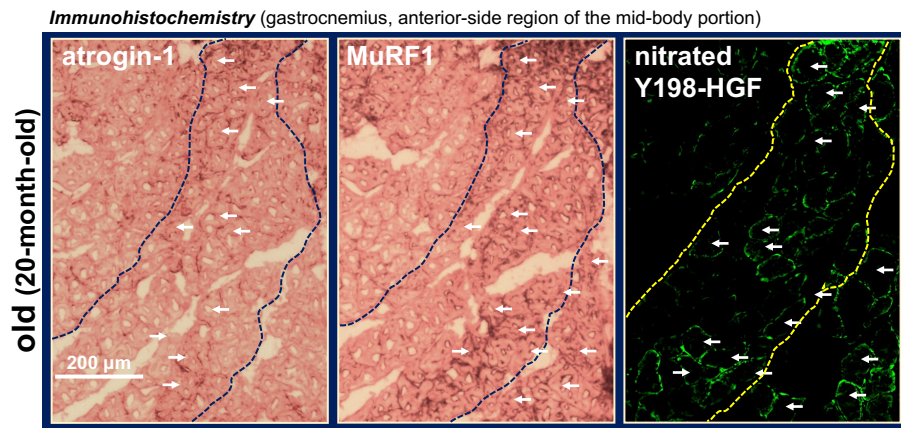

**Fig. S10, Elgaabari *et al.***

Supplement: Supplementary file 10 — Figure S10 [file ACEL-23-e14041-s009.pdf]
