# Supplementary material for: Age‐related nitration/dysfunction of myogenic stem cell activator HGF
Source: Aging Cell. 2023 Nov 20;23(2):e14041. doi: 10.1111/acel.14041 (PMC10861216; doi:10.1111/acel.14041)
Supplement: Supplementary file 2 — Figure S2 [file ACEL-23-e14041-s005.zip › Fig.S2C-E.pdf]

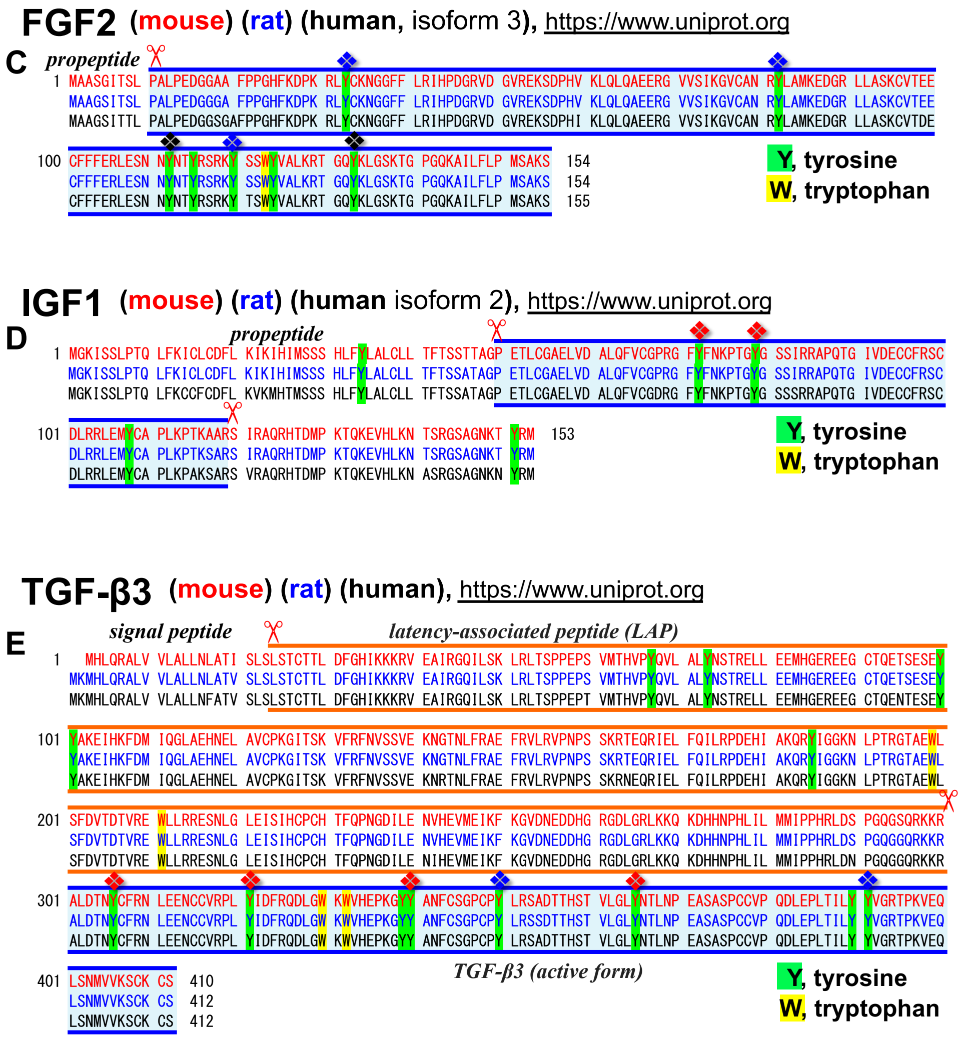

Fig. S2 C-E, Elgaabari *et al.*

(Supplemental to Fig. 3 B-D)
