# Supplementary material for: Age‐related nitration/dysfunction of myogenic stem cell activator HGF
Source: Aging Cell. 2023 Nov 20;23(2):e14041. doi: 10.1111/acel.14041 (PMC10861216; doi:10.1111/acel.14041)
Supplement: Supplementary file 3 — Figure S3 [file ACEL-23-e14041-s011.pdf]

Supplemental Materials

human HGF (NK2 segment)

A **Displayed based on:**
PDB ID: 3SP8 (PAN+K1+K2) x 2
Recacha *et al*. (2012)

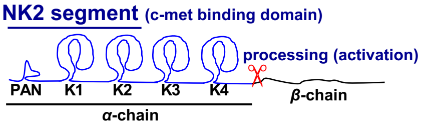

https://www.uniprot.org/uniprot/P14210

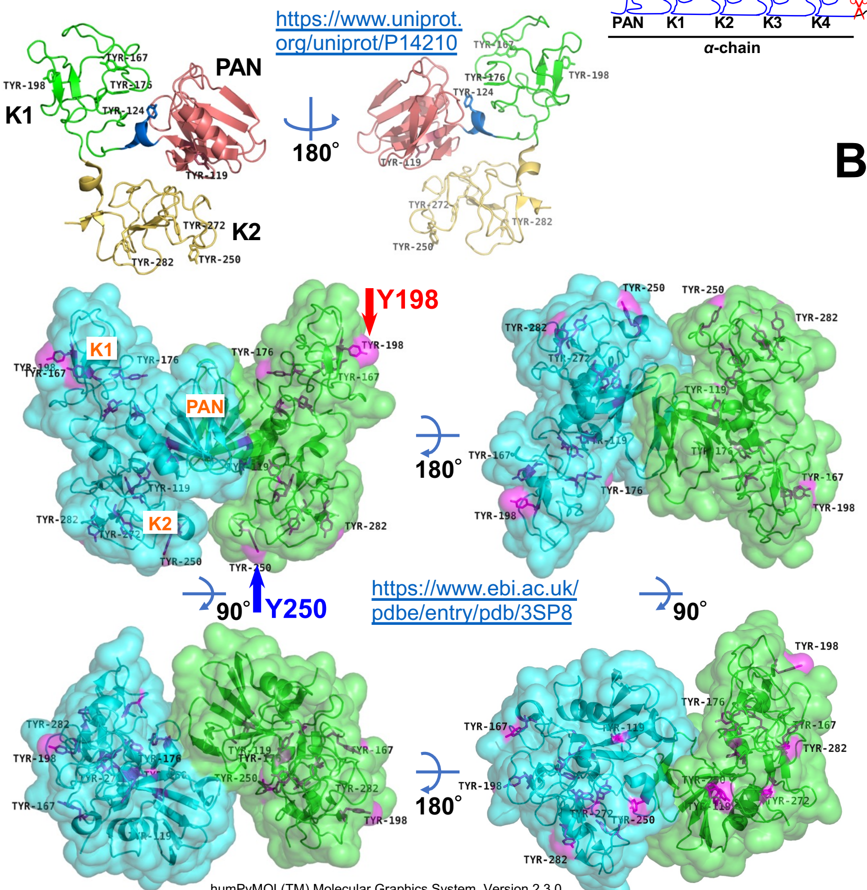

humPyMOL(TM) Molecular Graphics System, Version 2.3.0.
Copyright (c) Schrodinger, LLC.,
Created by Warren L. DeLano, Ph.D.

B **Displayed based on:**
PDB ID: 3HN4 (PAN+K1+K2)
Tolbert *et al*. (2010)

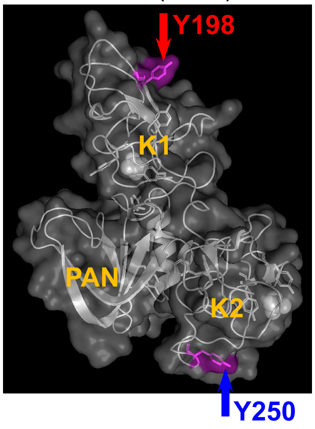

Displayed based on:
PDB ID: 7MO7
EMDB ID: EMD-23919
(HGF+c-MET1+c-MET2)
Uchikawa *et al*. (2021)

C

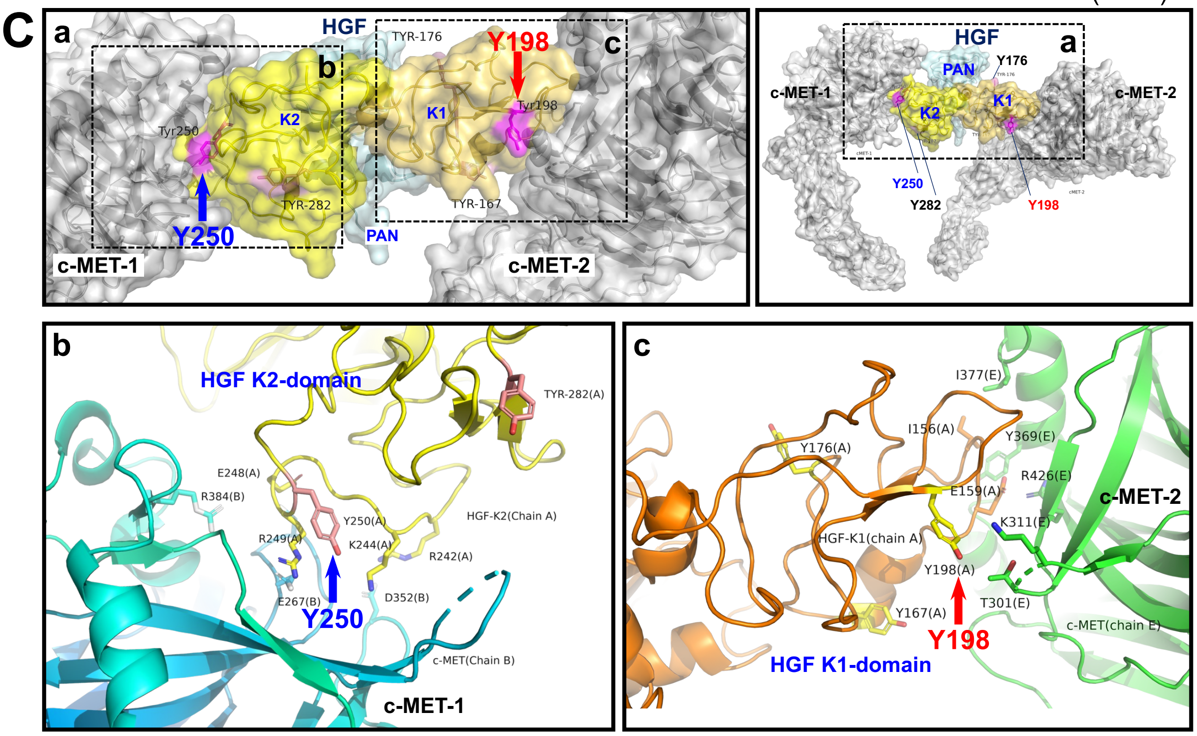

Fig. S3, Elgaabari *et al.*
(Supplemental to Fig. 3 A)
