# Supplementary material for: Age‐related nitration/dysfunction of myogenic stem cell activator HGF
Source: Aging Cell. 2023 Nov 20;23(2):e14041. doi: 10.1111/acel.14041 (PMC10861216; doi:10.1111/acel.14041)
Supplement: Supplementary file 11 — Table S1 [file ACEL-23-e14041-s008.docx]

**Table S1. Accessible surface areas of tyrosine residues in major growth factors.**

| **Protein (human)** | **PDB ID** ^a)^ | **Tyrosine (residue #)** | **ASA (Å^2^)** ^b)^ | | | |
| --- | --- | --- | --- | --- | --- | --- |
|  |  |  | **All** | **Side chain (SC)** | **Cε atoms (% Cε_1_, _2_ /SC)** | |
|  |  |  |  |  | **Cε_1_　 Cε_2_** | |
| HGF (NK2 segment) | 3HN4 ^c)^ | 119 | 12.2 | 12.2 | 0.0 (0.0) | 0.1 (0.8) |
|  |  | 124 | 22.2 | 11.8 | 1.1 (9.3) | 0.0 (0.0) |
|  |  | 136 | 4.0 | 2.2 | 0.0 (0.0) | 0.0 (0.0) |
|  |  | 167 | 40.9 | 38.3 | 14.0 (36.6) | 2.1 (5.5) |
|  |  | 176 | 45.6 | 45.6 | 7.9 (17.6) | 12.0 (26.3) |
|  |  | 198 | 92.8 | 76.7 | 9.1 (11.9) | 18.1 (23.6) |
|  |  | 219 | 8.5 | 3.2 | 0.8 (25.0) | 0.0 (0.0) |
|  |  | 250 | 78.4 | 74.1 | 27.8 (37.5) | 0.0 (0.0) |
|  |  | 259 | 17.0 | 17.0 | 1.9 (11.2) | 6.5 (38.2) |
|  |  | 272 | 16.3 | 16.0 | 0.0 (0.0) | 1.9 (11.9) |
|  |  | 282 | 63.6 | 63.2 | 21.9 (34.7) | 0.6 (0.9) |
| FGF2 | 1BAS ^d)^ | 25 | 72.6 | 72.6 | 11.4 (15.7) | 16.6 (22.9) |
|  |  | 74 | 52.4 | 52.4 | 7.5 (14.3) | 8.4 (16.0) |
|  |  | 104 | 47.8 | 47.8 | 9.7 (20.3) | 0.6 (1.3) |
|  |  | 107 | 0.3 | 0.3 | 0.0 (0.0) | 0.0 (0.0) |
|  |  | 112 | 55.4 | 50.1 | 18.3 (36.5) | 0.0 (0.0) |
|  |  | 116 | 27.8 | 27.4 | 1.9 (6.9) | 1.3 (4.7) |
|  |  | 125 | 52.8 | 45.2 | 2.3 (5.1) | 17.8 (39.4) |
| IGF1 | 1WQJ ^e)^ | 24 | 142.8 | 142.1 | 13.6 (9.6) | 32.1 (22.6) |
|  |  | 31 | 181.6 | 170.3 | 25.3 (14.9) | 32.0 (18.8) |
|  |  | 60 | 6.5 | 5.0 | 3.2 (64.0) | 0.0 (0.0) |
| TGF-β3 (active form) | 1TGJ ^f)^ | 6 | 144.3 | 116.1 | 20.7 (17.8) | 2.2 (1.9) |
|  |  | 21 | 130.9 | 108.9 | 15.7 (14.4) | 11.6 (10.7) |
|  |  | 39 | 23.9 | 19.3 | 0.0 (0.0) | 0.0 (0.0) |
|  |  | 40 | 129.4 | 123.6 | 10.0 (8.1) | 24.2 (19.6) |
|  |  | 50 | 106.8 | 84.4 | 0.7 (0.8) | 18.5 (21.9) |
|  |  | 65 | 105.1 | 100.2 | 23.9 (23.9) | 6.4 (6.4) |
|  |  | 90 | 24.9 | 24.9 | 0.0 (0.0) | 7.6 (30.5) |
|  |  | 91 | 71.0 | 57.7 | 0.2 (0.3) | 11.9 (20.6) |

^a)^Research Collaboratory for Structural Bioinformatics (RCSB) Protein Data Bank ID

^b)^Accessible Surface Area

^c)^<https://www.rcsb.org/structure/3HN4> (Primary publication DOI: [10.1073/pnas.1005183107](http://dx.doi.org/10.1073/pnas.1005183107), Tolbert *et al*. 2010)

^d)^<https://www.rcsb.org/structure/1BAS> (Primary publication DOI: [10.1126/science.1702556](http://dx.doi.org/10.1126/science.1702556), Zhu *et al*. 1991)

^e)^[https://www.rcsb.org/structure/1WQJ](https://www.rcsb.org/structure/1WQJ%20) (Primary publication DOI: [10.1016/j.str.2004.11.009](http://dx.doi.org/10.1016/j.str.2004.11.009), Siwanowicz *et al*. 2005)

^f)^[https://www.rcsb.org/structure/1TGJ](https://www.rcsb.org/structure/1TGJ%20) (Primary publication DOI: [10.1002/pro.5560050705](http://dx.doi.org/10.1002/pro.5560050705), Mittl *et al*. 1996)

Tyrosine residues having SC-ASA values over 30 Å^2^ were assigned to “exposed” and highlighted in yellow here (see Table 2 in Lins *et al*. 2003, DOI: 10.1110/ps.0304803) and indicated by arrows in Fig. 3 (black, SC-ASA 30-50; blue, 50-100; red, over 100).
