# Supplementary material for: Age‐related nitration/dysfunction of myogenic stem cell activator HGF
Source: Aging Cell. 2023 Nov 20;23(2):e14041. doi: 10.1111/acel.14041 (PMC10861216; doi:10.1111/acel.14041)
Supplement: Supplementary file 15 — Data S4 [file ACEL-23-e14041-s015.docx]

**SUPPORTING INFORMATION**

*Aging Cell*

Research Article

**Age-related nitration/dysfunction of myogenic stem cell activator HGF**

Alaa Elgaabari^1,2,a^, Nana Imatomi^1,a^, Hirochika Kido^1,a^, Takashi Nakashima^3^, Shoko Okuda^1^, Yoshitaka Manabe^1^, Shoko Sawano^1^, Wataru Mizunoya^1^, Ryuki Kaneko^1^, Sakiho Tanaka^1^, Takahiro Maeno^1^, Yuji Matsuyoshi^1^, Miyumi Seki^1^, So Kuwakado^4^, Kahona Zushi^1^,

Nasibeh Daneshvar^5^, Mako Nakamura^1^, Takahiro Suzuki^1^, Kenji Sunagawa^6^,

Judy E. Anderson^5^, Ronald E. Allen^7^, Ryuichi Tatsumi^1,^*

^1^Department of Animal and Marine Bioresource Sciences, Graduate School of Agriculture, Kyushu University, West Zone 5, Motooka 744, Nishi-ku, Fukuoka 819-0395, Japan

^2^Department of Physiology, Faculty of Veterinary Medicine, Kafrelsheikh University, El-Geish street, Kafrelsheikh 33516, Egypt

^3^Department of Bioscience and Biotechnology, Graduate School of Agriculture, Kyushu University, West Zone 5, Motooka 744, Nishi-ku, Fukuoka 819-0395, Japan

^4^Department of Orthopaedic Surgery, Faculty of Medical Sciences, Kyushu University, Maidashi 3-1-1, Higashi-ku, Fukuoka 812-8582, Japan

^5^Department of Biological Sciences, Faculty of Science, University of Manitoba, Winnipeg, MB R3T 2N2, Canada

^6^Department of Cardiovascular Medicine, Graduate School of Medicine, Kyushu University, Maidashi 3-1-1, Higashi-ku, Fukuoka 812-8582, Japan

^7^The School of Animal and Comparative Biomedical Sciences, University of Arizona, Tucson, Arizona 85721, USA

^a^These authors contributed equally.

*Correspondence: Ryuichi Tatsumi (Email: [rtatsumi@agr.kyushu-u.ac.jp](mailto:rtatsumi@agr.kyushu-u.ac.jp))

**Contents:**

1. Methodology (the entire Materials and Methods section)
2. Supplementary Table 1 (Table S1)
3. Supplementary Figures 1-10 (Figs. S1-S10) and Legends, attached separately
4. Original un-cropped Western blotting images (for Figs. 2G and 4D)
5. Source data (for Figures 1, 2, S1, S8, and S9), available upon request

**METHODOLOGY (the entire Materials and Methods section)**

**1. Animal care and use**

All experiments involving animals were conducted in strict accordance with the recommendations in the Guidelines for Proper Conduct of Animal Experiments published by the Science Council of Japan and ethics approvals from the Kyushu University Institutional Review Board (approval No. A20-014, A22-082, A28-092, and A30-143).

**2. Satellite cell isolation and primary culture**

Satellite cells were isolated from muscle in the upper hind limb and back of adult male Sprague-Dawley rats (9 to 10-month-old) according to Allen et al. (1997) with a slight modification (Tatsumi et al., 2006b). Briefly, muscles were collected, trimmed of connective tissue and fat, minced with sterile scissors, then digested with 1.25 mg/ml of protease type XIV (P5147 of Sigma-Aldrich, St. Louis, MO, USA) for 1 h at 37˚C. Cells were separated from muscle tissue debris by differential centrifugation and filtration (nylon cell strainers, 100-µm and 40-µm mesh size) prior to a final centrifugation step at 1500 x g for 3 min, then suspended in Dulbecco’s modiﬁed Eagle’s medium (DMEM; 31600-034 of Invitrogen, Grand Island, NY, USA) containing 10% (V/V) normal horse serum (HS; 16050-122), 1% antibiotic-antimycotic mixture (15240-062), and 0.5% gentamicin (15710-064) (DMEM-10% HS) and plated on poly-L-lysine (P9155, Sigma-Aldrich) and fibronectin (F1141)-coated plates. Cultures were maintained in a humidified atmosphere of 5% CO_2_ at 37˚C for 24 h and then incubated for the next 24-h period in DMEM-10% HS additionally containing recombinant HGF (2207-HG/CF, R&D Systems, Minneapolis, MN, USA) that was pre-treated with peroxynitrite (as described below).

In stretch experiments (Figure 1), cells were plated on BioFlex amino-culture plates (BFAMIN, Flexcell International, McKeesport, PA, USA) for 24 h and then subjected to mechanical stretch for as short as 1 h in culture in a vacuum-operated cyclic strain-providing instrument (FlexerCell FX-2000 System, Flexcell International) equipped with a BioFlex baseplate (see Figure 1A for the experimental design); cultures were maintained in DMEM-10% HS for the next 23-h period without stretch. The percentage of stretch and the length of the stretch interval were previously optimized for their effect on satellite cell activation to arrive at the final conditions of 25% stretch at 12-s intervals (see a low-frequency-with-hold (LFH) pattern is depicted in Figure 1B first row; originally described in Tatsumi et al. (2001, 2002). Other stretch-patterns with the same 25% stretch were designed and applied to the cultures in the same manner: LF (same frequency of stretch as LFH, 5 cycles/min, without a 5-sec stretch-hold phase) and HF (higher stretch-frequency than LFH and LF, 8.6 cycles/min) (see Figure 1B second and third rows, respectively). The positive control cultures received 2.5 ng/ml recombinant HGF (294-HG, R&D Systems) for 24 h from 24 to 48-h post-plating instead of stretch, as previously reported (Tatsumi et al., 1998).

In experiments in which conditioned media from stretched cultures were assayed for their activation activity and HGF release from the extracellular tethering (Figure 1D, E), cultures were washed with serum-free DMEM at 24-h post-plating and then exposed to one of the LFH/LF/HF-stretch environments for 1 h in serum-free DMEM (pH 7.2). Conditioned medium from each culture was collected, centrifuged at 1,300 x g for 4 min, filtered through a 0.22-µm pore-sized sterile filter, and prepared for enhanced-chemiluminescence (ECL)-Western blotting and satellite cell activation assay (see Figure 1A for the experimental design). In the case of immunoneutralization experiments, anti-HGF neutralizing polyclonal antibody raised in goat (AB-294-NA, R&D Systems; 2 µg/ml of the final concentration in culture media) was added to selected treatment-conditioned media for 2 h at 4˚C before the activation assay; purified goat anti-mouse IgG was used as the control antibody (Cappel Research, Durham, NC, USA). Horse serum and an antibiotic-antimycotic mixture were added to each treatment medium to prepare DMEM-10% HS immediately prior to feeding unstretched cultures, which were then maintained for the 24-h period from 24 to 48-h post-plating and assayed for satellite cell activation (Figure 1E).

**3. In vitro activation assay**

Cultures were pulse-labeled with 10 µM BrdU (B5002, Sigma-Aldrich) in DMEM-10% HS for the final 2 h of 48-h in culture (*i.e.*, 48-h post-, the fixation time-point), followed by immunocytochemistry for detection of BrdU, using G3G4 anti-BrdU monoclonal antibody (mAb; 1:100 dilution in 0.1% bovine serum albumin (BSA) in phosphate-buffered saline (PBS); obtained from Developmental Studies Hybridoma Bank, Iowa City, IA, USA) and horseradish peroxidase (HRP)-conjugated anti-mouse IgG Ab (A4416 from Sigma-Aldrich; 1:500 dilution) according to Tatsumi et al. (1998). The mean percentage of BrdU-labeled cells for three cultures per treatment was used as an indicator of activation (entry into the cell cycle) and the subsequent proliferation activity of plated satellite cells.

In addition, companion satellite-cell cultures, prepared at the same time, were immunostained for the presence of desmin at 30 h after plating, using a D3 anti-desmin mAb (obtained from Developmental Studies Hybridoma Bank) in order to determine the percentage of myogenic cells present (Allen et al., 1995; Tatsumi et al., 1998); cultures with less than 95% 3,3'-diaminobenzidine (DAB)-positive cells were not used for experiments.

**4. Peroxynitrite treatment of growth factors**

Recombinant mouse HGF (2207-HG/CF, carrier protein-free; a disulfide-linked heterodimer of α- and β-chains is the major form in the product purchased from R&D Systems) was evaluated for susceptibility to tyrosine nitration by incubating with peroxynitrite under physiological conditions and by subsequent Western blotting to detect nitro-tyrosines (3-NTs). Briefly, peroxynitrite (P332, manufactured by Dojindo Lab., Kumamoto, Japan; 0.1 M stock solution in 0.3 M NaOH-NaCl) was diluted to less than 1/100 with ice-cold water, just prior to quick exposure to HGF in PBS by vortexing at molar ratios (HGF:peroxynitrite) of 1:100, 1:200, 1:500, 1:1000, 1:2000, and 1:4000 at pH 7.1-7.6, 37˚C. HGF with solvent alone (without adding peroxynitrite) was assigned as the control (1:0; see Figure 2A for the experimental design). Recombinant mouse FGF2 (3139-FB/CF, R&D Systems), human IGF1 (95119, FUJIFILM Irvine Scientific, Santa Ana, CA, USA), and human TGF-β3 (8420-B3/CF, R&D Systems) were also treated with peroxynitrite in the same manner for 30 min at pH 7.4, 37˚C. Additionally, HGF was incubated for 30 min at pH 7.4, 25˚C with SIN-1 (S264, Dojindo Lab.; 3-(4-morpholinyl)sydnonimine), which spontaneously generates NO and O_2_^-^ radicals to produce peroxynitrite continuously for longer periods under physiological conditions.

**5. ECL-Western blotting**

Conditioned media from stretched cultures were subjected to sodium dodecylsulfate-10% polyacrylamide gel electrophoresis (SDS-10% PAGE) under reducing conditions in Laemmli’s buffer system; sample volume was normalized by cell density on the culture dishes at 24-h post-plating (stretch-starting time-point) (Tatsumi et al., 2001, 2002) in order to compare the HGF releasability in cells cultured under LFH-, LF-, and HF-stretch paradigms at a constant number of cultured satellite cells (2500 cells/well). Separated proteins were transferred to nitrocellulose membranes. Blots were blocked with 5% (w/v) non-fat powdered milk in 0.1% (v/v) polyethylene sorbitan monolaurate (Tween 20) in Tris-buffered saline (TTBS) for 30 min, prior to incubation with anti-HGF polyclonal antibody (AB-294-NA, R&D Systems; 1:500 dilution in CanGetSignal solution 1 (NKB-101, Toyobo, Osaka, Japan); overnight at 4˚C) and HRP-labeled donkey anti-goat IgG antibody (705-036-147, affinity-purified F(ab’)2 fragment from Jackson ImmunoResearch Lab., West Grove, PA, USA; 1:5000 dilution in CanGetSignal solution 2 for 1.5 h at room temperature), followed by enhanced chemiluminescence (ECL)-detection onto Kodak BioMax XAR films according to the manufacture’s recommendation (Amersham Pharmacia Biotech, Piscataway, NJ, USA), as described previously (Tatsumi et al., 1998).

Tyrosine nitration was visualized by Western blotting of peroxynitrite-treated HGF. Briefly, the blots were incubated overnight at 4˚C with HRP-conjugated anti-nitrotyrosine mouse mAb (clone 39B6, sc-32757 HRP from Santa Cruz Biotechnology, Dallas, TX, USA; 1:2500 dilution in CanGetSignal solution 1), followed by ECL-detection on a FUSION SOLO.7S.EDGE imaging system (Vilber Lourmat, Marne-la-Vallée, France). Subsequently, the blots proceeded through a second round of immunodetection by agitating (or without agitation; see Figure 2B second row) for 45 min at 50˚C in stripping buffer (2% SDS, 0.8% β-mercaptoethanol (βME), and 62.5 mM Tris-HCl, pH 6.8) (Elgaabari et al., 2022) followed by re-probing with a set of polyclonal anti-HGF (1:500 dilution in CanGetSignal solution 1) and HRP-conjugated anti-goat IgG antibodies (1:5000 dilution in CanGetSignal solution 2), or with HRP-labeled anti-HGF *N*-terminal domain mouse mAb (clone H-10, sc-374422 HRP from Santa Cruz Biotechnology, 1:2500 dilution in CanGetSignal solution 1) to monitor HGF amount on the blots as loading controls. Immunoblotting analysis of the nitration potential of recombinant FGF2, IGF1, and TGF-β3 was conducted in the same manner using HRP-conjugated anti-nitrotyrosine antibody and individual primary-detection antibodies (HRP-unconjugated): anti-FGF2 (05-118, MERCK Millipore, [Burlington, MA](https://en.wikipedia.org/wiki/Burlington,_Massachusetts), USA; 1:10000 dilution), anti-IGF1 (AF-291-NA, R&D Systems; 1:5000), or anti-TGF-β3 antibodies (MAB243, R&D Systems; 1:1000). The membranes were then washed with TTBS and incubated with the corresponding HRP-conjugated secondary antibodies, followed by ECL-detection on a FUSION SOLO.7S.EDGE imaging system. This approach enabled specific, direct detection of nitrotyrosine residues in each growth factor, followed by detection of the growth factor itself, by direct (HGF) and/or indirect (FGF2, IGF1, TGF-β3, and HGF) immunodetection. Reprobing the blot and re-staining for the growth factor illustrated the size (MW) of any nitrated peptide.

**6. c-Met binding assay (Sandwich ELISA-like assay)**

ELISA plates (E.I.A./R.I.A. 96-well microplates, Corning Life Sciences, NY, USA) were coated overnight at 25˚C with HGFR/c-MET Fc chimera (7065-ME, carrier protein-free; R&D systems) by adding 100 µl of 1 µg protein/ml in PBS to each well, which was then blocked for 2 h at 25˚C with 1% BSA, 5% sucrose, and 0.05% sodium azide in PBS (150 μl/well). Plates were washed with TPBS and incubated for 2 h at 37˚C with 50 µl of peroxynitrite-treated HGF (at molar ratios of 1:0, 1:100, 1:2000, and 1:4000, at pH 7.4). Plates were subsequently washed with TPBS, immediately fixed with cold 3.7% (v/v) paraformaldehyde in PBS for 5 min at 4˚C and re‑treated with the blocking solution overnight at 4˚C. The binding of peroxynitrite-treated HGF to c-met was detected with 50 µl of biotinylated anti-HGF polyclonal antibody (BAF294, R&D systems; 1:500 dilution in the 0.1% BSA-TTBS for 3 h at 25˚C), TACS streptavidin-HRP conjugate (4800-30-06, a BioTechne brand from R&D systems; 1:1600 dilution for 20 min), and 3,3’,5,5’-tetramethylbenzidine (TMB) substrate solution (DY999, R&D systems), followed by optical density measurements at wavelengths of 450 and 540 nm according to Tatsumi et al. (2002) with minor modifications.

Synthetic HGF-peptide pairs that include both nitrated and non-nitrated Y198 and Y250, FTSNPEVR_nitro/non-nitro_Y_198_EV and KFLPER_nitro/non-nitro_Y_250_PDKGFD described later, were conjugated with BSA and then evaluated for their c-met binding activity, in the same way except a) with omission of BSA from all incubation steps and b) using HRP-labeled anti-BSA mAb (clone 2A3E6; sc-32816 HRP from Santa Cruz Biotechnology) as the detection antibody.

**7. Accessible surface area calculation for tyrosine residues**

Primary structures of HGF, FGF2, IGF1, and TGF-β3 were obtained from The Universal Protein Resource (UniProt; https://www.uniprot.org) that is a collaboration between the [European Bioinformatics Institute (EMBL-EBI)](https://www.ebi.ac.uk/), the [SIB Swiss Institute of Bioinformatics](https://www.sib.swiss/), and the [Protein Information Resource (PIR](https://proteininformationresource.org/)) to provide a comprehensive resource for protein sequence and annotation data (see Figure 3A-D upper columns and Figure S2).

High-resolution crystal structures of the human growth factors were from Research Collaboratory for Structural Bioinformatics Protein Data Bank (RCSB PDB); ID: 3HN4 and 3SP8 were used for the NK2 segment (*N*-terminal domain + K1 domain + K2 domain) of HGF α-chain (Tolbert et al., 2010; Recacha et al., 2012), 1BAS was for FGF2 (Zhu et al., 1991), 1WQJ was for IGF (Siwanowicz et al., 2005), and 1TGJ was for TGF-β3 (Mittl et al., 1996). The accessible surface area (ASA) values for all atoms, side-chain (SC) atoms, and the Cε atoms (% Cε_1, 2_/SC) of tyrosine residues in growth factors concerned were calculated using a program AREAIMOL of the CCP4 package (1994) (with a probe radius of 1.4 Å to provide an index for exposure of tyrosine residues, especially of their side chains, from the protein surface (see Table S1). The Cryo-EM structure of a HGF molecule complexed with the receptor c-met (RCSB PDB ID: 7MO7, with 4.8 Å resolution) (Uchikawa et al., 2021) was re-drawn focusing on tyrosine residues by the program PyMOL Molecular Graphics System (Version 2.3.0; copyright: Schrödinger, LLC., created by Dr. Warren L. DeLano) (see Figure S3C).

**8. Production of anti-nitrated HGF monoclonal antibodies**

Monoclonal antibody production was performed in a 2018-2019 period by a rat lymph node method established by Sado et al. (1995), in which enlarged medial iliac lymph nodes are used as a source of B cells that are fused to myeloma cells to generate hybridomas; this results in highly efficient and rapid antibody production. Building upon the primary structure of HGF α-chain and ASA values of tyrosine residue (Y) side-chains, we selected two sequences that include Y198 and Y250, FTSNPEVRY_198_EV and KFLPERY_250_PDKGFD, completely conserved in mouse, rat, cat, dog, and human (see Figures 3A and S2A,B), for custom synthesis by Sigma-Aldrich in the forms containing nitrated Y198 and Y250. Peptides with non-nitrated Y198 and Y250 were also synthesized as controls. A cysteine residue (C) was added to the *N*-terminus of nitrated and non-nitrated peptides to enable conjugation with a carrier protein of BSA (A4503, Sigma-Aldrich) or keyhole limpet hemocyanin (KLH; 151233, a MP Biomedicals brand of FUJIFILM Wako Pure Chemical, Osaka, Japan). Carrier proteins (20 mg/ml) were column-desalted, incubated with 3-maleimidobenzoic acid *N*-hydroxysuccinimide ester (MBS; M2786, Sigma-Aldrich; 15 mg/ml at final concentration) for 30 min, and then incubated with the nitrated Y198, Y250 peptides (5 mg/ml) at pH 7.0 with stirring for 3 h. The conjugated peptide solutions were stored at -20˚C until use.

Hind footpads of 10-week-old male WKY/Izm rats (Japan SLC, Hamamatsu, Japan) received an injection of KLH-conjugated nitrated Y198 and nitrated Y250 peptides (antigens; 100 μl solution) emulsified with Freund’s complete adjuvant (263810, Difco Lab., NJ, USA). After 2 wks, B cells from the lymph nodes of immunized rats were fused with mouse myeloma SP2/0-Ag14 cells (provided by the RIKEN BRC, Tsukuba, Japan) at a ratio of 5:1 in 50% polyethylene glycol (PEG1500, Roche, Basel, Switzerland); the obtained hybridoma cells were seeded onto 96-well plates and cultured in HAT selection medium, hybridoma SFM medium (12300067, Invitrogen, Waltham, MA, USA) additionally containing 10% fetal bovine serum (FBS), 1 ng/ml recombinant human interleukin 6 (IL-6; 206-IL, R&D Systems), 100 μM hypoxanthine (H9377, Sigma-Aldrich), 0.4 μM aminopterin (A3411, Sigma-Aldrich), and 16 μM thymidine (205-08091, FUJIFILM Wako Pure Chemical).

At 6-days post-plating, conditioned media from hybridoma cultures were first-screened by ELISA against BSA-conjugated nitrated/non-titrated Y198 and Y250 peptides that were immobilized to 96-well flexible microplates by incubating the individual peptide solutions (2 µg/ml) overnight at 4˚C, followed by treatment with the blocking solution (1% BSA in PBS) prior to incubation with hybridoma supernatants for 1 h at room temperature. Immunoreactivity was visualized by HRP-conjugated anti-rat IgG antibody (A5795, Sigma-Aldrich; 1:20000 dilution in 1% BSA-PBS, for 30 min at room temperature) and TMB substrate. From the first ELISA screening, we selected 36 hybridomas positive for nitrated Y198 antigen and negative for non-nitrated Y198 and nitrated Y250 peptides (Figure S4A), while 30 hybridomas were positive for nitrated Y250 peptide but neither non-nitrated Y250 peptide or nitrated Y198 peptide (Figure S4B). The second ELISA-screening was performed by assessing the immuno-specificity to nitrated HGF (recombinant full-length HGF treated with peroxynitrite at 1:2000 molar ratio at pH 7.2) and the growth/proliferation activity of hybridomas, by which two hybridomas 3A1 and 8B7 were obtained for the nitrated Y198 peptide and two others, 3C3 and 6B8, were obtained for the nitrated Y250 peptide (Figure S4C).

These hybridomas were single-cloned by limiting dilution cultures; 13 clones from 3A1 and 8B7 and 17 clones from 3C3 and 6B8 were examined for the immuno-specificity on ELISA plates pre-coated with individual nitrated or non-nitrated Y198 or Y250 peptides (Figure S5A,B) or with nitrated/non-nitrated HGF (Figure S5C). Three 3A1-derived clones (1B3, 1C6, 1G8) and three 6B8-derived clones (2A4, 2C3, 2G10) were selected and further evaluated for their immuno-reactivities by Western blotting of nitrated/non-nitrated HGF (Figure S5D,E) to establish hybridoma clones 1C6 (from 3A1) and 2C3 (from 6B8) that specifically recognize nitrated Y198-HGF and Y250-HGF proteins, respectively, under both reducing and non-reducing conditions.

Finally, clones 1C6 and 2C3 were reconfirmed for clonality and immuno-specificity by a series of re-screening steps consisting of a second limiting dilution culture, ELISA, and Western blotting (data not shown), and were designated 3A11C6 and 6B82C3 as the first hybridoma clones producing mAbs against nitrated Y198-HGF and nitrated Y250-HGF, respectively. Immunoglobulin isotypes of clones 3A11C6 and 6B82C3 were both determined to be rat IgG2a(κ), subclass G2a for heavy chains and kappa for light chains by Rat Monoclonal Antibody Isotyping Test Kit (RMT1, Bio-Rad Lab., Berkeley, CA, USA) (Figure S5F). Immunoglobulins in 3A11C6 and 6B82C3 culture supernatants were concentrated by HiTrap Protein G HP columns (GE Healthcare, Little Chalfont, UK) and labeled with fluorescent probes using a Fluorescein Labeling Kit-NH2 (LK01, Dojindo Lab.) and a HiLyte Fluor 647 Labeling Kit-NH2 (LK15, Dojindo Lab.), respectively, for immunofluorescence detection of nitrated HGF.

**9. Immunofluorescence microscopy and histochemistry**

Male Sprague-Dawley (S.D.) rats were housed at 22 ± 2˚C and 55 ± 10% humidity on a 12-h light/dark cycle (lights on at 8 a.m.) with free access to regular food (CRF-1, Oriental Yeast, Tokyo, Japan) and deionized water. To examine whether ECM-bound HGF undergoes nitration during aging, lower hind-limb muscles were collected from three age groups, young (2-month-old), adult (10-month-old), and old (20-month-old) rats; muscles were oriented in tissue OCT compound in cryo-molds for cross-section and frozen in isopentane cooled with liquid nitrogen (n = 3 rats per group). Serial cryo-sections (mid-belly portions, 13-μm thickness) were prepared using a Leica CM1950 cryostat (Nussloch, Germany), and fixed with hot PBS and steam for 5 min (Sawano et al., 2016). After cooling to room temperature, sections were blocked with sterile donkey-serum solution (containing 2% normal serum, 1% BSA, 0.1% cold fish skin gelatin, 0.05% Tween 20, 0.01% avidin, 0.05% sodium azide, and 100 mM glycine in PBS, pH 7.2) for 1 h at 25˚C prior to incubation overnight at 4˚C with Fluorescein-labeled anti-nitrated Y198-HGF (clone 3A11C6), HiLyte Fluor 647-labeled anti-nitrated Y250-HGF (clone 6B82C3), or Alexa Fluor 594-labeled anti-HGF α-chain antibody (clone H-10; sc-374422 AF594 from Santa Cruz Biotechnology; 1:100 dilution in the sterile solution containing 1% BSA, 0.1% cold fish skin gelatin, 0.05% Tween 20, 0.01% biotin, and 0.05% sodium azide in PBS). Sections were mounted in VECTASHIELD Antifade Mounting Medium (H1000-10, Vector Lab., Burlingame, CA, USA) and observed under a Leica DMI6000B-AFC (Wetzlar, Germany) or KEYENCE BZ-X700 (Osaka, Japan) fluorescence microscope, each equipped with digital cameras and Tile-Scan programs (see Figures 4A-C, 5, 6, S6, S7, S10).

A serial section of each specimen was used to visualize myofiber types by quadruple, direct immunostaining of fast/slow myosin expression according to a “stained glass-like staining” procedure developed by co-authors, Dr. Sawano and Dr. Mizunoya (Sawano et al., 2016). In brief, single cryo-sections were incubated overnight at 4˚C in the mixture of primary mAbs raised in-house against myosin heavy chain (MyHC) type I (slow) and types IIa, IIx, and IIb (fast) (pre-labeled with Molecular Probes Alexa Fluor 647, 350, Fluorescein, and HyLyte Fluor 594, respectively, at 1:100 dilution in a sterile solution containing 1% BSA, 0.1% cold fish skin gelatin, 0.5% Triton X-100, 0.05% Tween 20, 0.01% biotin, and 0.05% sodium azide in PBS). Images were captured by a KEYENCE BZ-X700 microscope equipped with a Tile-Scan program and analyzed for fiber-type composition and total fiber numbers in cross-sections of whole calf and shank muscle groups from each of the three age groups (Figures 5, 6, and S8B,C).

Additional sets of cryo-muscle sections were stained with modified Masson’s trichrome staining reagents (Muto Pure Chemicals, Tokyo, Japan) to visualize age-related muscular fibrosis. Briefly, cryo-sections were hydrated and immersed in the first mordant for 25 min, followed by10-min water-wash. Specimens were then placed in Weigert’s iron hematoxylin solution, rinsed with running water for 10 min, dipped in the second mordant for 30 sec, and immersed in 0.75% Orange G solution for 1 min, Masson’s stain solution B for 25 min, 2.5% phosphotungstic acid for 20 min, and aniline blue for 15 min in this order, passing through 1% acetic acid between each step. Finally, slides were dehydrated with ethanol, permeated with xylene, and sealed with Mount-Quick (DAI-DM-01, Daido Sangyo, Toda, Japan) for KEYENCE BZ-X700 microscopic observation (Figure S8D).

**10. Extraction of extracellular HGF**

Extraction of extracellular matrix-associated HGF was conducted according to Naldini et al. (1992); cultured satellite cells from three aged-groups were rinsed with warm DMEM at 24-h post-plating and subsequently incubated twice with sterile 1.0 M NaCl in DMEM at 37˚C for 5 min each. NaCl washes were saved, centrifuged to remove cellular debris, dialyzed against a solution containing 5 mM EDTA and 5 mM Tris-HCl, pH 8.0 for 2 h, and solubilized in Laemmli’s SDS-βME sample buffer as described previously (Tatsumi et al., 2001; Tatsumi & Allen, 2004). Samples were concentrated by centrifugal ultra-filtration devices (Amicon Ultra, 10-kDa MWCO) and subjected to ECL-Western blotting using anti-nitrated Y198-HGF mAb (clone 3A11C6) and HRP-conjugated anti-rat IgG secondary antibody (ab6734, Abcam, Cambridge, MA, USA) to monitor the nitration levels of extracellular HGF normalized to cell count on the plate (Figure 4D).

**11. Statistical analyses**

Student’s *t-*tests were employed for statistical analysis of experimental results using Microsoft Excel X for Windows (Figures 1C,E, 2F,H,J, S1, S8A-C, and S9). Data are represented as mean ± standard error of the mean (SEM). The level of significance was set to *p* < 0.05 throughout this study and statistically significant differences between two groups at *p* < 0.05, *p* < 0.01, and *p* < 0.001 are indicated throughout by (*), (**), and (***), respectively. The results are representative examples of more than two or three independent experiments.

**LEGENDS TO SUPPLEMENTARY FIGURES**

**FIGURE S1. Dysfunction of HGF in conditioned media from high-frequent (HF) stretch cultures.**

Activation activity of conditioned media from 1-h LFH/HF stretch cultures of satellite cells that were prepared from 9-10-month-old adult S.D. rats (supplemental to Figure 1 panels D-F). Assayed at 48-h post-plating, at 24-h after feeding conditioned media from unstretched control culture (*bar a*), LFH-stretch (*bar b*), HF-stretch (*bar c*). *Bar d*, 1:1 mixture of LFH- and HF-conditioned media; *bar e*, freshly prepared control medium; *bar f*, positive control with 2.5 ng/ml HGF. Bars represent mean ± SEM; significant differences at *p <* 0.05 and *p <* 0.01 are indicated by (*) and (**), respectively.

**FIGURE S2. Comparative display of whole primary structures of major growth factors (Supplemental to Figure 3).**

**(A, B)** Primary structures of human (black), cat (blue), and dog HGF (red) **(A)** and of mouse (red), human (black), and chicken HGF (green) **(B)** were displayed in the same manner as Figure 3A. Y198/Y250-containing sequences, FTSNPEVRY_198_EV and KFLPERY_250_PDKGFD underlined in black, are completely conserved in mammals examined (Y198 is missing in chicken), indicating that our anti-nitrated Y198-HGF and anti-nitrated Y250-HGF mAbs raised in-house are expected to be immuno-reactive to mammalian HGF.

**(C-E)** Primary structures of FGF2, IGF1, and TGF-β3 of mouse (red), rat (blue), and human (black). Original data are from UniProt (<https://www.uniprot.org>). Tyrosine (Y) and tryptophan (W) residues are highlighted by green and yellow, respectively; tyrosine residues having SC-ASA values 30-50 Å^2^, 50-100 Å^2^, and over 100 Å^2^ are indicated by black, blue, and red rhomboids, respectively, in the same manner as Figure 3 (see Table S1 for more details).

**FIGURE S3. Three-dimensional structure for the NK2 segment of HGF, (represented in ribbon/space-filling diagram with focusing on Y198 and Y250).**

**(A)** High-resolution crystal structure of NK2 segment (containing c-met binding domains) of human HGF, displayed based on RCSB PDB ID 3SP8 (Tolbert et al., 2010) (a dimer of NK2 segment (PAN+K1+K2 domains)) with pink-highlighting all tyrosine residues including Y198 and Y250 indicated by red and blue arrows, respectively.

**(B)** A monomer of human NK2 segment. Displayed according to PDB ID 3HN4^57^.

**(C)** A complex of human NK2 segment sandwiched by the receptor c-met molecules (c-MET-1 and c-MET-2, grey-colored) is depicted, based on PDB ID 7MO7 (Uchikawa et al., 2021) (originally displaying a whole HGF molecule bound to two c-met molecules) and focused on residues Y198 and Y250 along with Y176 and Y282. The lower row is a set of enlarged views of boxed areas b and c shown in upper row. Note that both Y198 and Y250 localize in c-met binding sites. The 3D-structures were drawn by the program PyMOL Molecular Graphics System (Version 2.3.0).

**FIGURE S4. Generation of anti-nitroY198-HGF and Y250-HGF monoclonal antibodies (screening).**

**(A, B)** Monoclonal antibody production was performed by a rat lymph node method established by Sado et al*.* (1995). Two sequences that include Y198 and Y250, FTSNPEVRY_198_EV and KFLPERY_250_PDKGFD were synthesized in the forms containing nitrated Y198 and Y250. Peptides with non-nitrated Y198 and Y250 were also synthesized as controls. A cysteine residue was added to the *N*-terminal of peptides to enable conjugation with a carrier protein of BSA or KLH. Hind footpads of male WKY/Izm rats received an injection of KLH-conjugated nitrated Y198 and nitrated Y250 peptides (antigens). B cells from the lymph nodes were fused with myeloma SP2/0-Ag14 cells; the obtained hybridoma cells were seeded onto 96-well plates and cultured in HAT selection medium. Conditioned media from hybridoma cultures were first-screened by ELISA against BSA-conjugated nitrated/non-titrated Y198 and Y250 peptides immobilized to 96-well microplates. Immunoreactivity was visualized by HRP-conjugated anti-rat IgG antibody and TMB substrate. By the first ELISA screening, 36 hybridomas positive for nitrated Y198 antigen and negative for non-nitrated Y198 and nitrated Y250 peptides were selected (**A**), while 30 hybridomas positive for nitrated Y250 peptide but not either non-nitrated Y250 peptide or nitrated Y198 peptide (**B**).

**(C)** The second ELISA-screening was performed by assessing the immuno-specificity to nitrated HGF (recombinant full-length HGF treated with peroxynitrite) and the growing/proliferation activity of hybridomas; hybridomas 3A1 and 8B7 for the nitrated Y198 peptide (*upper-half a*) and 3C3 and 6B8 for the nitrated Y250 peptide were obtained (*lower-half b*).

**FIGURE S5. Generation of anti-nitroY198-HGF and Y250-HGF monoclonal antibodies (cloning).**

Single-cloning of hybridomas by limiting dilution cultures. Selected 13 clones from 3A1 and 8B7 and 17 clones from 3C3 and 6B8 were examined for the immuno-specificity to individual nitrated/non-nitrated Y198 peptides **(A)** and nitrated/non-nitrated Y250 peptides (**B**) and to nitrated/non-nitrated HGF **(C)**. 3A1-derived clones (1B3, 1C6, 1G8, red-circled) and 6B8-derived clones (2A4, 2C3, 2G10, blue-circled) were selected and further evaluated by Western blotting of nitrated/non-nitrated HGF **(D, E)** to establish hybridoma clones 1C6 and 2C3 that specifically recognize nitrated Y198-HGF and Y250-HGF proteins, respectively, under both reducing and non-reducing conditions. After the second limiting dilution culture to reconfirm the clonality and immuno-specificity (data not shown), clones 1C6 and 2C3 were designated 3A11C6 and 6B82C3 as the hybridoma clones producing mAbs against nitrated Y198-HGF and nitrated Y250-HGF, respectively. Immunoglobulin isotypes of clones 3A11C6 and 6B82C3 were both determined to be rat IgG2a(κ) as revealed by the appearance of the corresponding light blue-bands on a rat mAb isotyping test strips (**F**; indicated by arrowheads).

**FIGURE S6. Extracellular HGF undergoes nitration during aging (Supplemental to Figure 4A,B).**

Direct-immunofluorescence micrographs of muscle from three age-groups (*n* = 3 rats per group; tiling images at low magnification), stained with Fluorescein-labeled anti-nitrated Y198-HGF (clone 3A11C6; fluorescent false-green) **(A)** or HiLyte Fluor 647-labeled anti-nitrated Y250-HGF (clone 6B82C3; fluorescent false-yellow) **(B).** Gas, gastrocnemius; Pla, plantaris; Sol, soleus muscle. See the Figure 4 legend for more details.

**FIGURE S7. HGF nitration status in muscle from adult (10-month-old) rat (Supplemental to Figures 4-6).**

Tibialis anterior (TA) muscle cross-sections were stained with Fluorescein-labeled anti-nitrated Y198-HGF (clone 3A11C6; left column) and HiLyte Fluor 647-labeled anti-nitrated Y250-HGF (clone 6B82C3; right column) with counter-staining with DAPI. Representative high-magnification micrographs from muscle of adult rat (10-month-old), which corresponds to the early-age time-point when HGF nitration appears to progress and accumulate faster than in the previous 2-10-month-old period observed in this study (see Figure S8 for aging status of the 10-month-old adult age-group). Note that, at this stage, the nitration of HGF was seen in clusters around myofibers (HGF is sequestered in the ECM) with particular prominence near nuclei between or beside fibers (indicated by yellow arrows).

**FIGURE S8. Aging status of three aged-groups of rats used in this study (Supplemental to Figures 4-6 and S7).**

**(A)** Body and muscle (back, thigh, calf, shank muscle) weights of three age-groups of male S.D. rats, young (2-month-old), adult (10-month-old), and old (20-month-old) (*n* = 3 each group). **(B)** Fiber-type composition of plantaris (Pla) and soleus (Sol) muscles, measured by quadruple direct-immunofluorescence staining of fast/slow myosin expression; see right column for representative images of calf muscle group composed of gastrocnemius (Gas), Pla, and Sol muscles (re-produced from Figure 5A at higher magnification).  **(C)** Fiber numbers in whole-muscle sections of plantaris and soleus. **(D)** Development of connective tissue (stained blue) visualized by modified Masson trichrome staining of EDL/TA (left column) and Gas/Pla/Sol muscle groups (right; tiling images). Bars represent mean ± SEM; significant differences between groups at *p <* 0.05, *p <* 0.01, and *p <* 0.001 are indicated beside lines connecting two bars, by (*), (**), and (***), respectively; +, *p <* 0.10 (panels A-C).

**FIGURE S9. Comparison of HGF-induced activation activity of cultured satellite cells from adult and old rats (Supplemental to Figures 4-6).**

Primary cultures of satellite cells from back and upper hind-limb muscles of adult (10-month-old) and old rats (20-month-old; male S.D.) were maintained in the presence (*solid bars b and d*) or absence (CNT; *open bars a and c*) of 5 ng/ml recombinant active HGF (a hetero-dimer of α- and β-chains), for 24 h from 24 to 48-h post-plating in DMEM‑10% HS; activation activity was evaluated by BrdU-pulse labeling from 46 to 48-h in culture followed by immunolocalization, as shown in Figure 2F (*n* = 3-4 rats per group). Bars represent mean ± SEM; significant differences at *p <* 0.01 are indicated by (**). *NS*, not significant at *p <* 0.05.

**FIGURE S10. Immunodetection of ubiquitin ligase expression in nitrated HGF-positive fibers (Supplemental to Figures 4-6).**

Cyo-sections of calf muscle (mid-belly portion) in the old rat (20-month-old) were immunostained with anti-atrogin-1 and anti-MuRF1 mAbs (clone F-9, sc-166806 and clone F-11, sc-398608 from Santa Cruz Biotech., respectively) and ImmPRESS Excel Amplified Micropolymer HRP Staining reagents (MP-7602 from Vector Lab.). Another serial section was stained with fluorescent probe-conjugated anti-nitrated Y198-HGF mAb as shown in Figures 4-6. Nitrated ECM-bound HGF-rich area in gastrocnemius muscle (anterior-side region of the mid-body portion) was evaluated for the colocalization between myofiber levels of nitrated HGF with expression of E3 ubiquitin ligases, atrogin-1 and MuRF1 (well-known markers of muscle atrophy). Dotted lines were drawn on the micrographs to adjust the alignment of the three images. Representative atrogin-1/MuRF1-positive fibers are indicated by white arrows to help understanding the notable proportions of the ubiquitin ligase-positive fibers in nitrated HGF-positive fibers. Note that this figure was added to the Supplemental Information during the revision of the paper, acknowledging that due to long-term storage and lack of available materials, samples with freezing artifacts (ice crystal formation and "edge effect") were used.
